# Supplementary material for: Interactions of substrates and phosphinyl containing inhibitors with bacterial and human zinc proteases
Source: PLoS One. 2025 Aug 1;20(8):e0329362. doi: 10.1371/journal.pone.0329362 (PMC12316308; doi:10.1371/journal.pone.0329362)
Supplement: S1 File — (PDF) [file pone.0329362.s001.pdf]

Supporting information

Interactions of substrates and phosphinyl containing inhibitors with bacterial and human zinc proteases

Fatema Amatur Rahman[1], Imin Wushur[1], Ida K. Ø. Hansen[2], Tor Haug[2], Klara Stensvåg[2], Bibek Chaulagain[1], Tra-Mi Nguyen[1], Olayiwola A. Adekoya[3], Nabin Malla[1], Jan-Olof Winberg[1], Ingebrigt Sylte[1,4*]

[1] Pharmacology and Toxicology Research Group, Department of Medical Biology, Faculty of Health Sciences, UiT The Arctic University of Norway, NO-9037 Tromsø, Norway
[2] Norwegian College of Fishery Science, Faculty of Biosciences, Fisheries and Economics, UiT The Arctic University of Norway, NO-9037 Tromsø, Norway
[3] Centre for Pharmacy, Department of Clinical Science, Faculty of Medicine, University of Bergen, Norway
[4] Center for Research and Education, University Hospital of North Norway (UNN), Tromsø, Norway.

* Corresponding author: ingebrigt.sylte@uit.no (IS)

S1 Table. MALDI-TOF MS analysis. MS data observed at time zero after incubation of 50 µM Mca-Arg-Pro-Pro-Gly-Phe-Ser-Ala-Phe-Lys(Dnp)-OH (ES005) with 10 nM thermolysin (TLN).

**Substrate/fragment**	**Formula**	**Calculated m/z $[M+H]^+$**	**Observed m/z $[M+H]^+$**
Substrate (ES005)	C66H81N15O19	1388.59	1388.62
Loss of O	C66H81N15O18	1372.59	1372.61
Loss of NO	C66H81N14O18	1358.59	1358.60
Loss of O2	C66H81N15O17	1356.60	1356.60
Loss of NO2	C66H81N14O17	1342.60	1342.60

S1 Fig

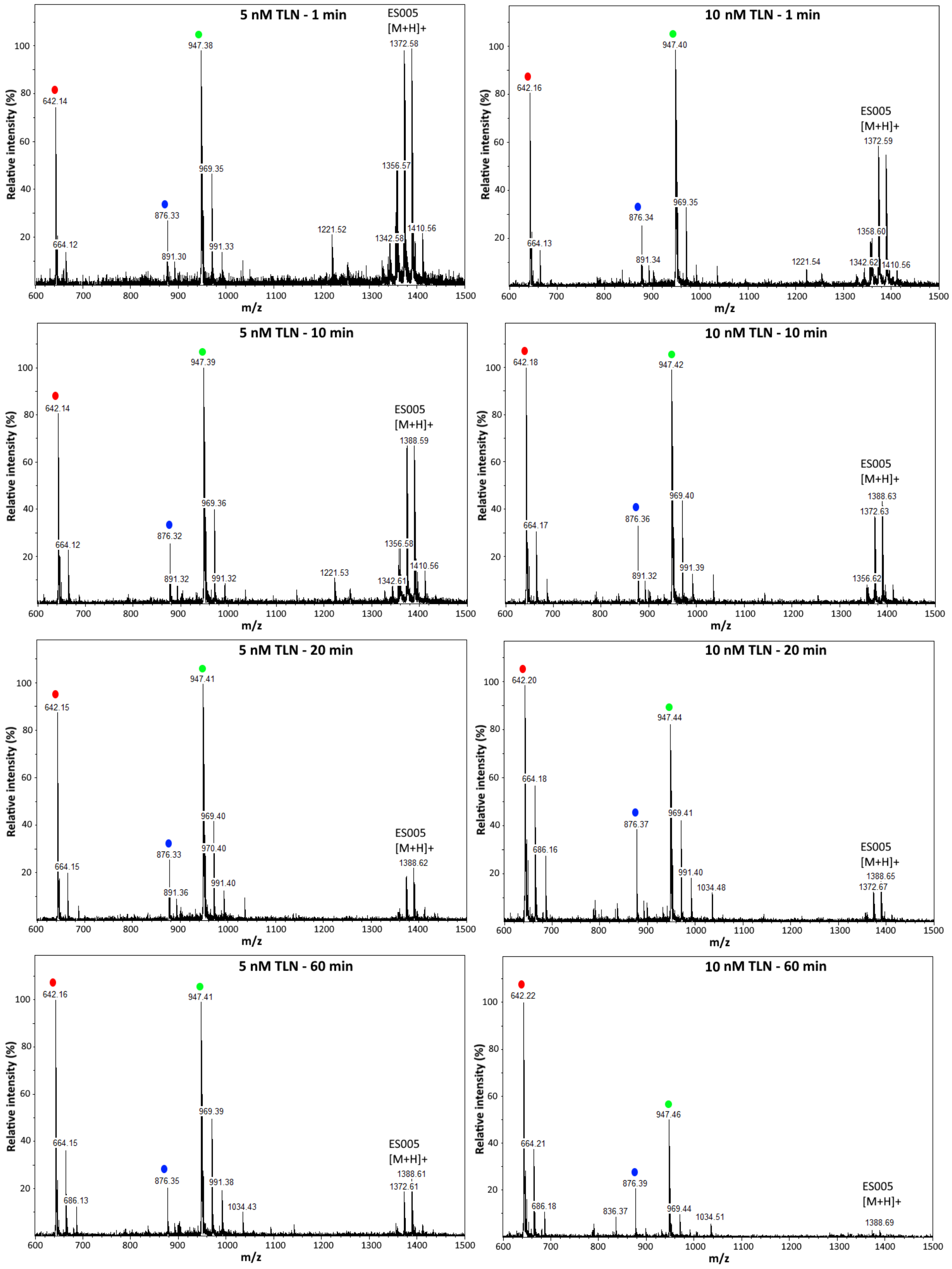

S1 Fig. MALDI-TOF MS analysis. MS spectra at different time points after incubation of 50 µM Mca-Arg-Pro-Pro-Gly-Phe-Ser-Ala-Phe-Lys(Dnp)-OH (ES005) with 5 or 10 nM thermolysin (TLN). The m/z values marked in red, blue and green represent N-terminal cleavage products after Gly-Phe, Ser-Ala, and Ala-Phe cleavage, respectively.

S2 Fig

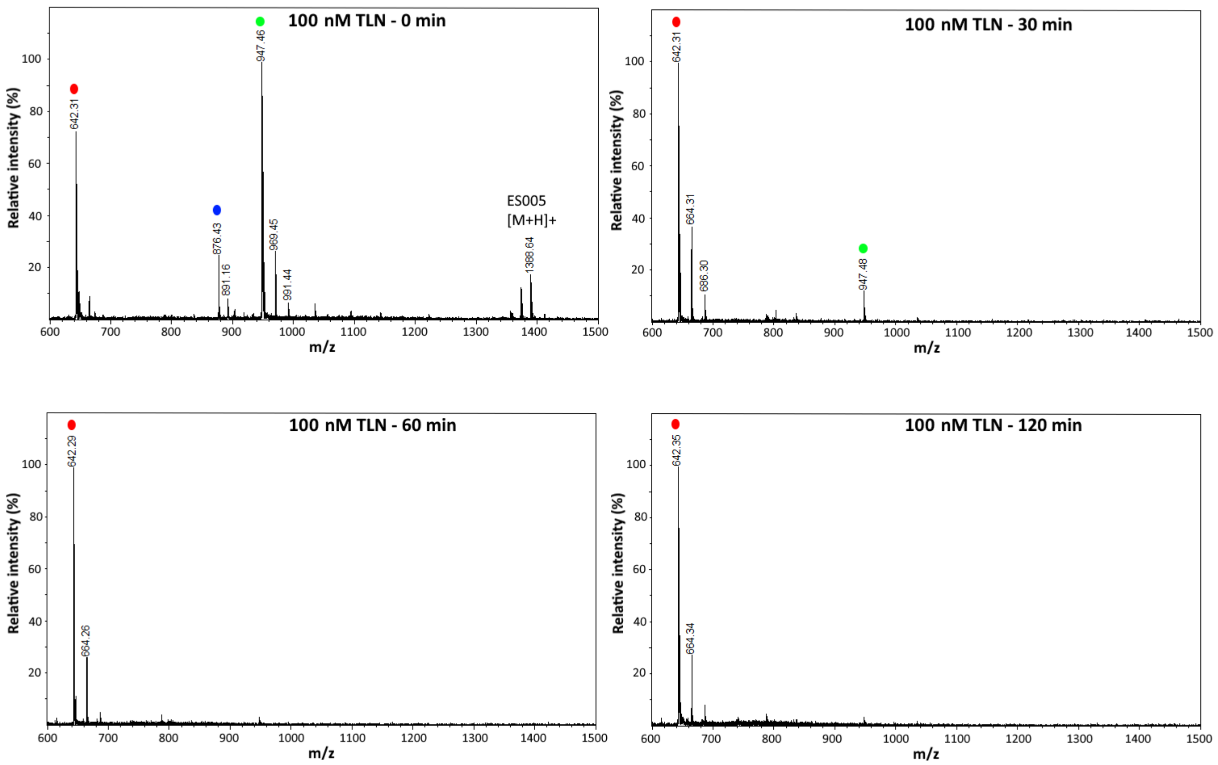

S2 Fig. MALDI-TOF MS analysis. MS spectra at different time points after incubation of 50 µM (McaRPPGFSAFPK(Dnp)-OH) ES005 with 100 nM thermolysin (TLN). The m/z values marked in red, blue and green, represents N-terminal cleavage products after Gly-Phe, Ser-Ala and Ala-Phe cleavage, respectively.

S3 Fig

MMP-9/ES001 complex

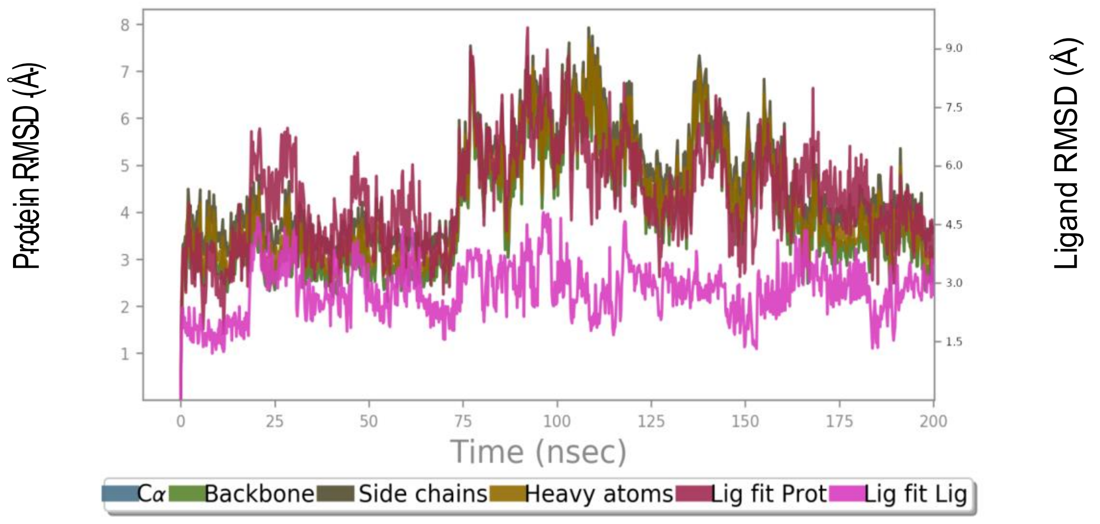

MMP-14/ES001 complex

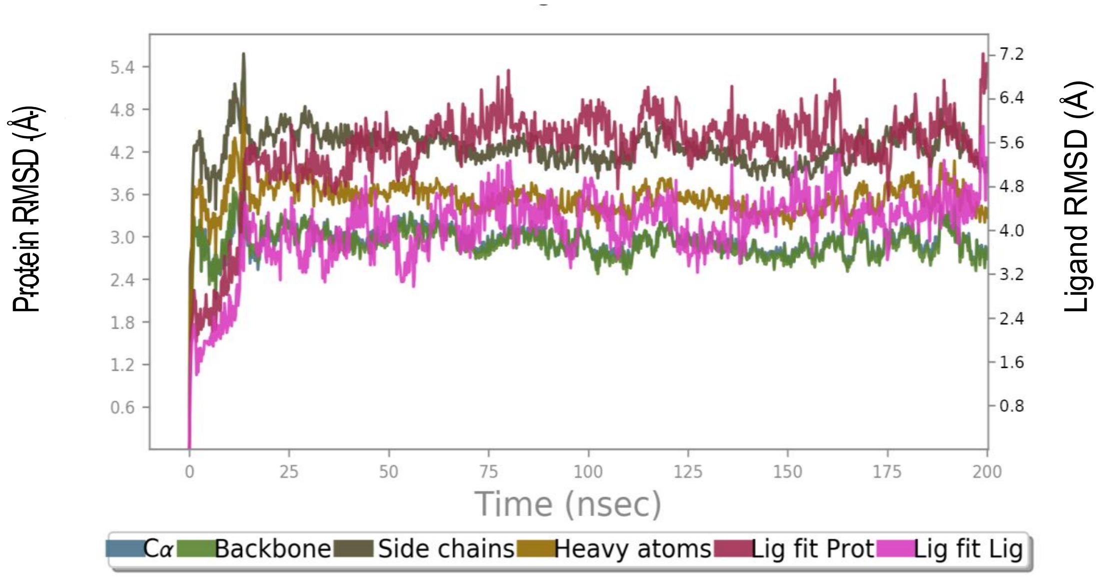

S3 Fig. Root means square deviations (RMSDs) during MDs. RMSDs of enzyme and ligand (Mca-Pro-Leu-Gly-Leu(Dpa)-Ala-Arg-NH_2, ES001) from the starting enzyme – ES001 complexes during 200 ns of molecular dynamics (MD) simulations. Above: ES001 with MMP-9. Below: ES001 with MMP-14.

S4 Fig

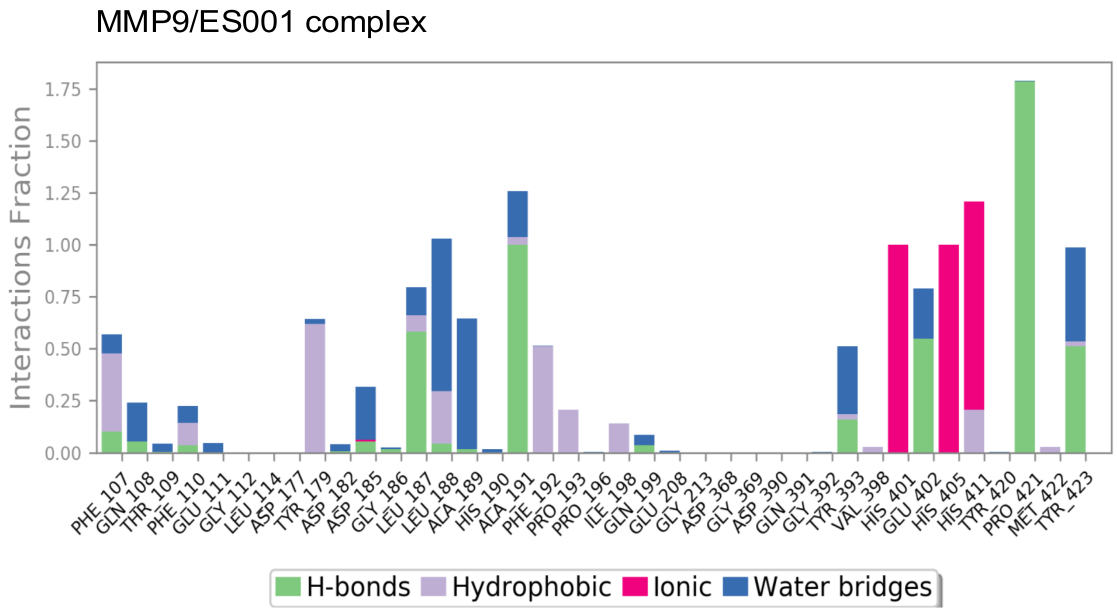

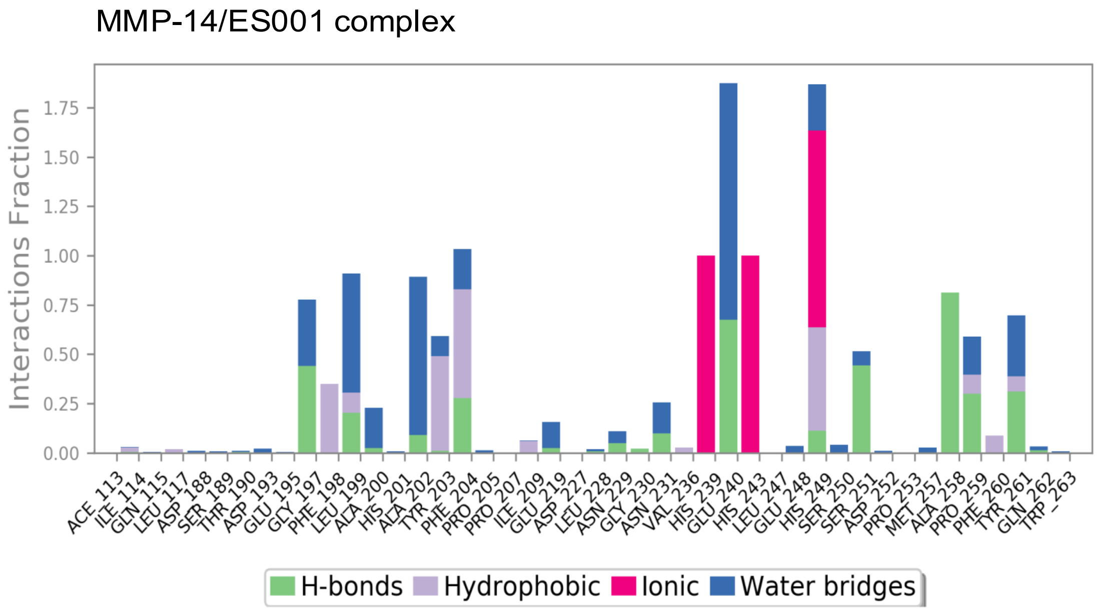

S4 Fig. ES001-enzyme interactions. Interaction fractions of amino acids of MMP-9 (above) and MMP-14 (below) with ES001 during 200 ns of MD simulation. Interaction fractions higher than one for the same subtype of interaction indicate that the amino acid makes multiple contacts with the substrate.

S5 Fig

TLN/ES005 - GF complex

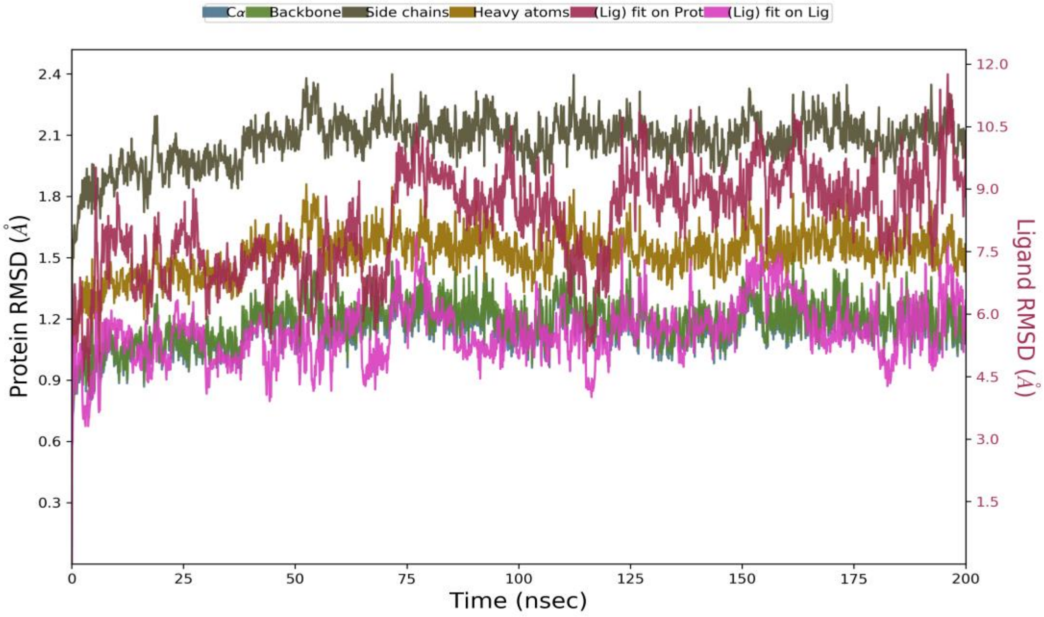

TLN/ES005 - AF complex

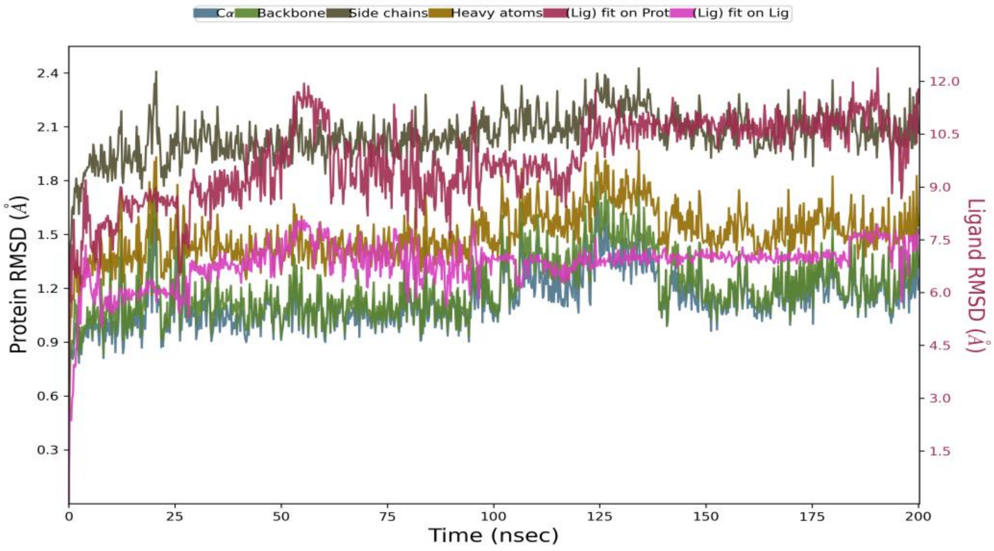

S5 Fig, continued

TLN/ES005 – SA complex

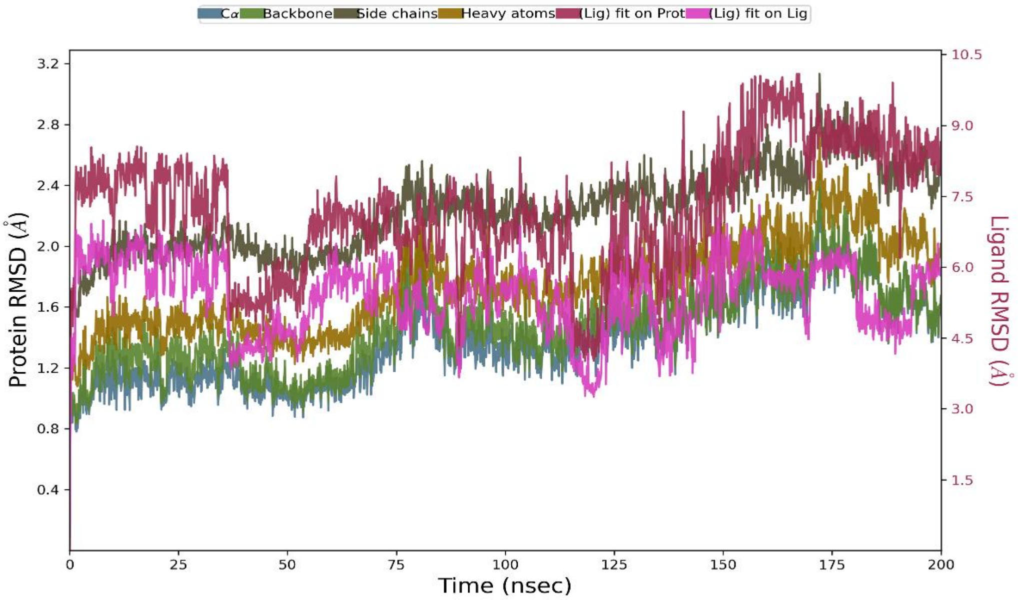

S5 Fig. Root means square deviations (RMSDs) during MDs. RMSDs of thermolysin (TLN) and ligand (McaRPPGFSAF(Dnp)-OH, ES005) from starting TLN – ES005 complexes during 200 ns of MD simulations.

S6 Fig

TLN/ES005 - GF complex

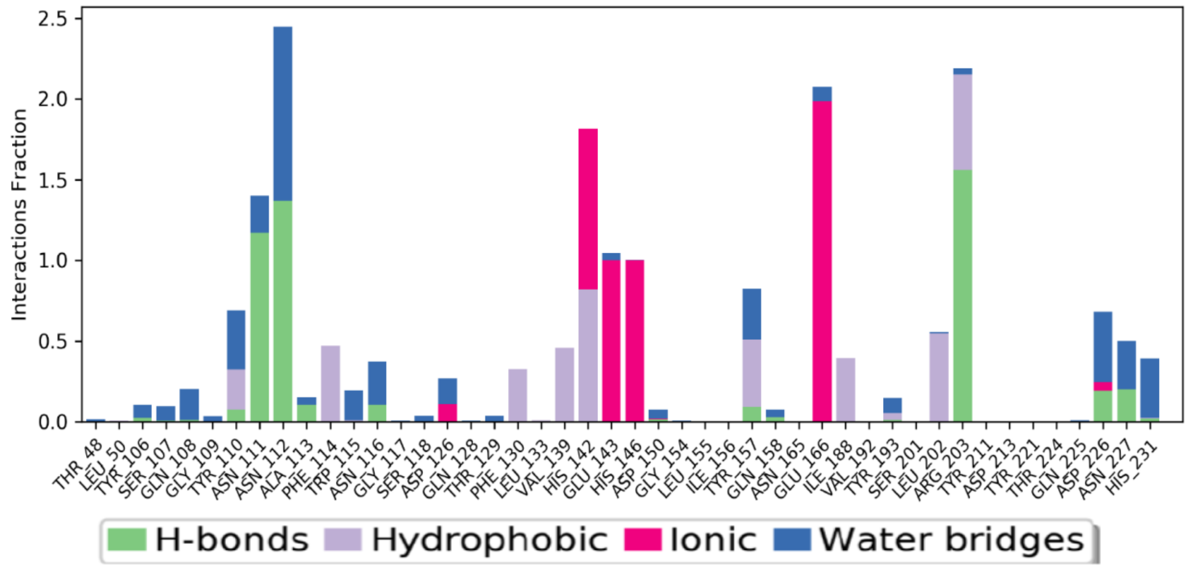

TLN/ES005 - AF complex

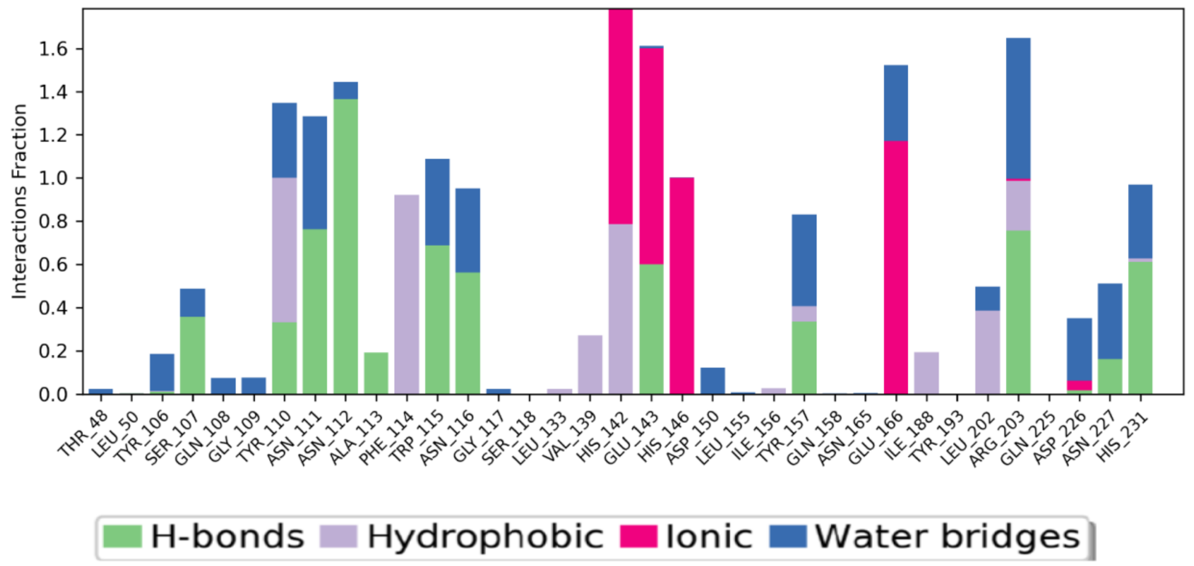

S6 Fig, continued

TLN/ES005 – SA complex

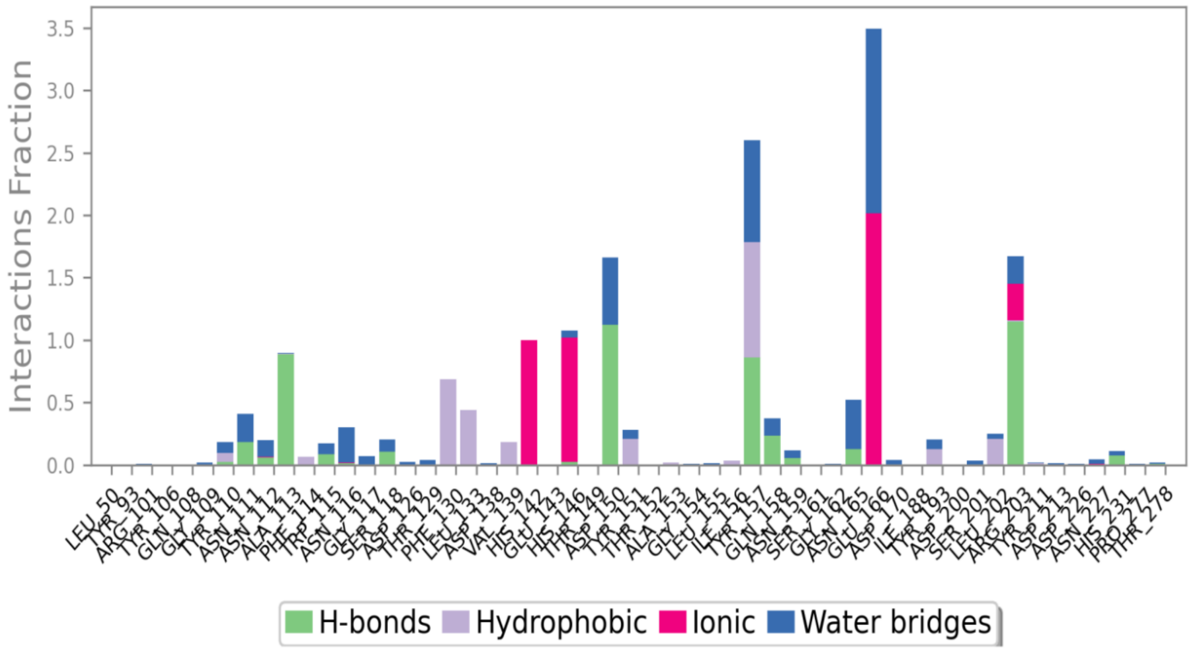

S6 Fig. ES005-TLN interactions. Interaction fractions of amino acids in TLN with the substrate McaRPPGFSAFK(Dnp)-OH (ES005) during 200 ns of MD simulations with Gly-Phe, Ala-Phe and Ser-Ala as P_1 and P_1' residues. respectively. Interaction fractions higher than one for the same subtype of interaction indicate that the amino acid makes multiple contacts with the substrate.

S7 Fig

```
PLN   ---AEAG----GPGGNQKIGKYTYGSDYGPLIVNDRCEMDDGNVITVDMNSSTDDSKTTP 53
TLN   ITGTSTVGVGRGVLGDQKNINTTYS-TYY--YLQDN--TRGNGIFTYDAK---------- 45
ALN   --EAAATGTGKGVLGDTKDININSI-DGGF-SLEDL--THQGKLSAYNFN---------- 44
         : :     *  *: *  : .         ::*      . : : : :

PLN   FRFACPTNT-----YKQVNGAYSPLNDAHFFGGVVFKLYRDWFGTSPLTHK---LYMKVH 105
TLN   YRTTLPGSLWADADNQFFASYDAPAVDAHYYAGVTYDYYKNVHNRLSYDGNNAAIRSSVH 105
ALN   -DQTGQATLITNEDENFVKDDQRAGVDANYYAKQTYDYYKNTFGRESYDNHGSPIVSLTH 103
         :   .       : . .      **:::.  .:. *:: ..      :   :   .*

PLN   YG-----RSVENAYWDGTAMLFGDGATMFYP--LVSLDVAAHEVSHGFTEQNSGLIYRGQ 158
TLN   YS-----QGYNNAFWNGSQMVYGDGDGQTFIPLSGGIDVVAHELTHAVTDYTAGLIYQNE 160
ALN   VNHYGGQDNRNNAAWIGDKMIYGDGDGRTFTNLSGANDVVAHELTHGVTQETANLEYKDQ 163
       .      . :** * *  *::***    :     . **.***::*..*: .:.* *:.:

PLN   SGGMNEAFSDMAGEAAEFYMRGKNDFLIGYDIK---KGSGALRYMDQPSRDGRSIDNASQ 215
TLN   SGAINEAISDIFGTLVEFYANKNPDWEIGEDVYTPGISGDSLRSMSDPAKYGDPDHYSKR 220
ALN   SGALNESFSDVFGYFV-----DDEDFLMGEDVYTPGKEGDALRSMSNPEQFGQPSHMKDY 218
      **.:**::**: *  .      . *: :* *:      ..:** *.:* : *   .  .

PLN   YYN---GIDVHHSSGVYNRAFYLLANSP--------GWDTRKAFEVFVDANRYYWTATSN 264
TLN   YTGTQDNGGVHINSGIINKAAYLISQGGTHYGVSVVGIGRDKLGKIFYRALTQYLTPTSN 280
ALN   VYTEKDNGGVHTNSGIPNKAAYNVI----------QAIGKSKSEQIYYRALTEYLTSNSN 268
            . .** .**: *:* * :            . .  *  :::  *   * * .**

PLN   YNSGACGVIRSAQNR----NYSAADVTRAFSTVGVTCPSAL      301
TLN   FSQLRAAAVQSATDLYGSTSQEVASVKQAFDAVGVK-----      316
ALN   FKDCKDALYQAAKDLYDEQTAE--QVYEAWNEVGVE-----      302
      :..   .  ::* :     . .  .* .*:. ***
```

S7 Fig. Amino acid sequence alignments. Amino acid sequence alignments of the catalytic domain of pseudolysin (PLN), thermolysin (TLN) and aurolysin (ALN)**.** SWISS-PROT accession codes; PLN: P14756, TLN: P00800, ALN P81177. The alignments were generated using Clustal Omega at the online services of EMBL-EBI (http://www.ebi.ac.uk/services). The zinc binding motif and the catalytic glutamic acid are highlighted in bold.

S8 Fig

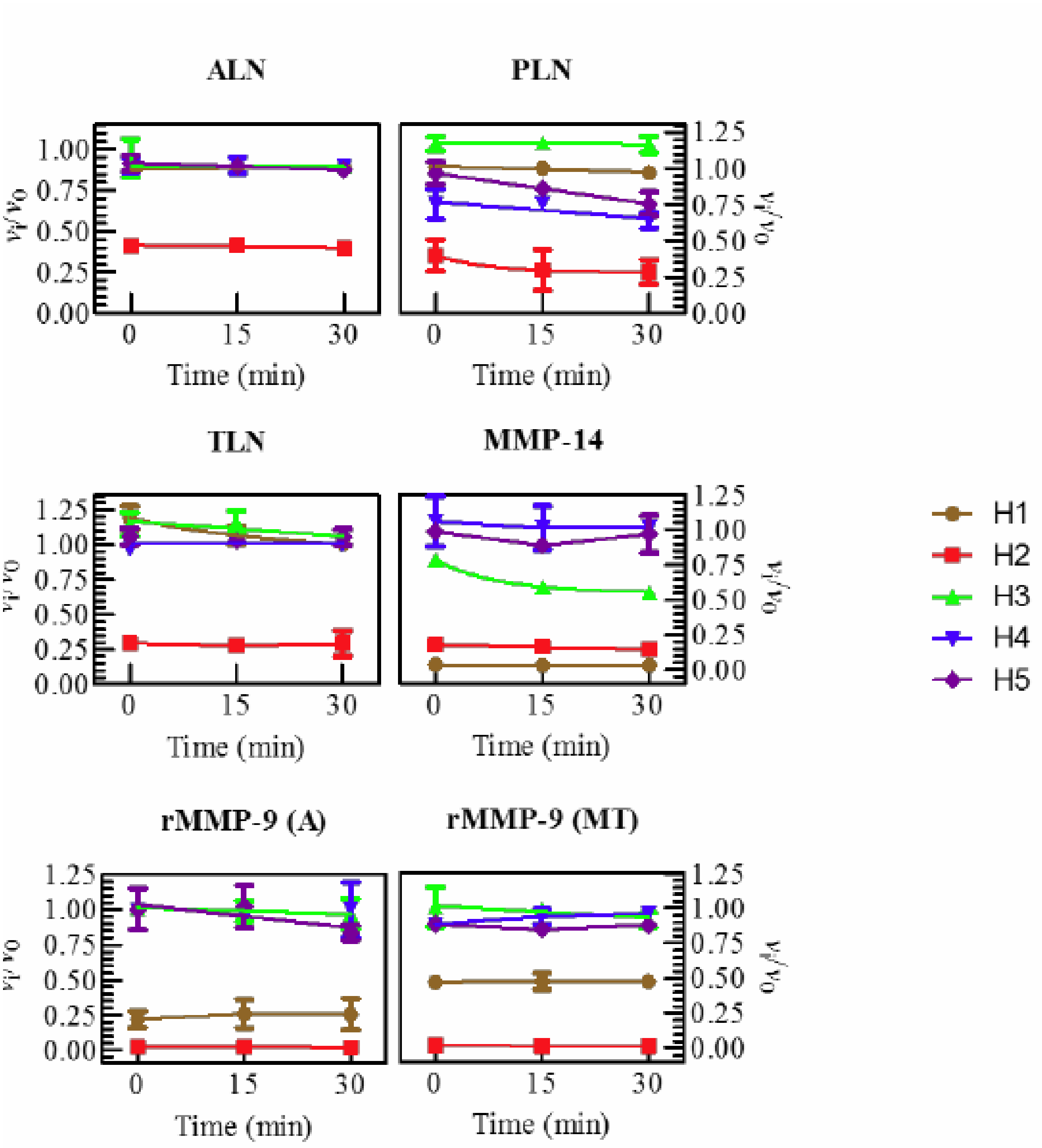

S8 Fig. Time dependent inhibition. Time dependent inhibitory effects of 100 µM of the compounds (H-1 - H-5) on the activity of aureolysin (ALN), pseudolysin (PLN), thermolysin (TLN), MMP-14, MMP-9 (A) and MMP-9 (MT). The experiments were performed as described in the material and methods section using a concentration of 4 µM of the substrate Mca-Pro-Leu-Gly(Dpa)-Ala-Arg-NH_2 (ES001) for MMP-9 (A), MMP-9 (T) and MMP-14, and of the substrate Mca-Arg-Pro-Pro-Gly-Phe-Ser-Ala-Phe-Lys(Dnp)-OH (ES005) for TLN, ALN and PLN. Concentrations of the enzymes were as follows: 0.05 nM MMP-9(A), 0.05 nM MMP-9(MT), 1.0 nM MMP-14, 1.4 nM ALN, 0.5 nM PLN and 0.21 nM TLN. The v_i/v_0 (mean ± s.d.) were based on 3-7 experiments.

S9 Fig

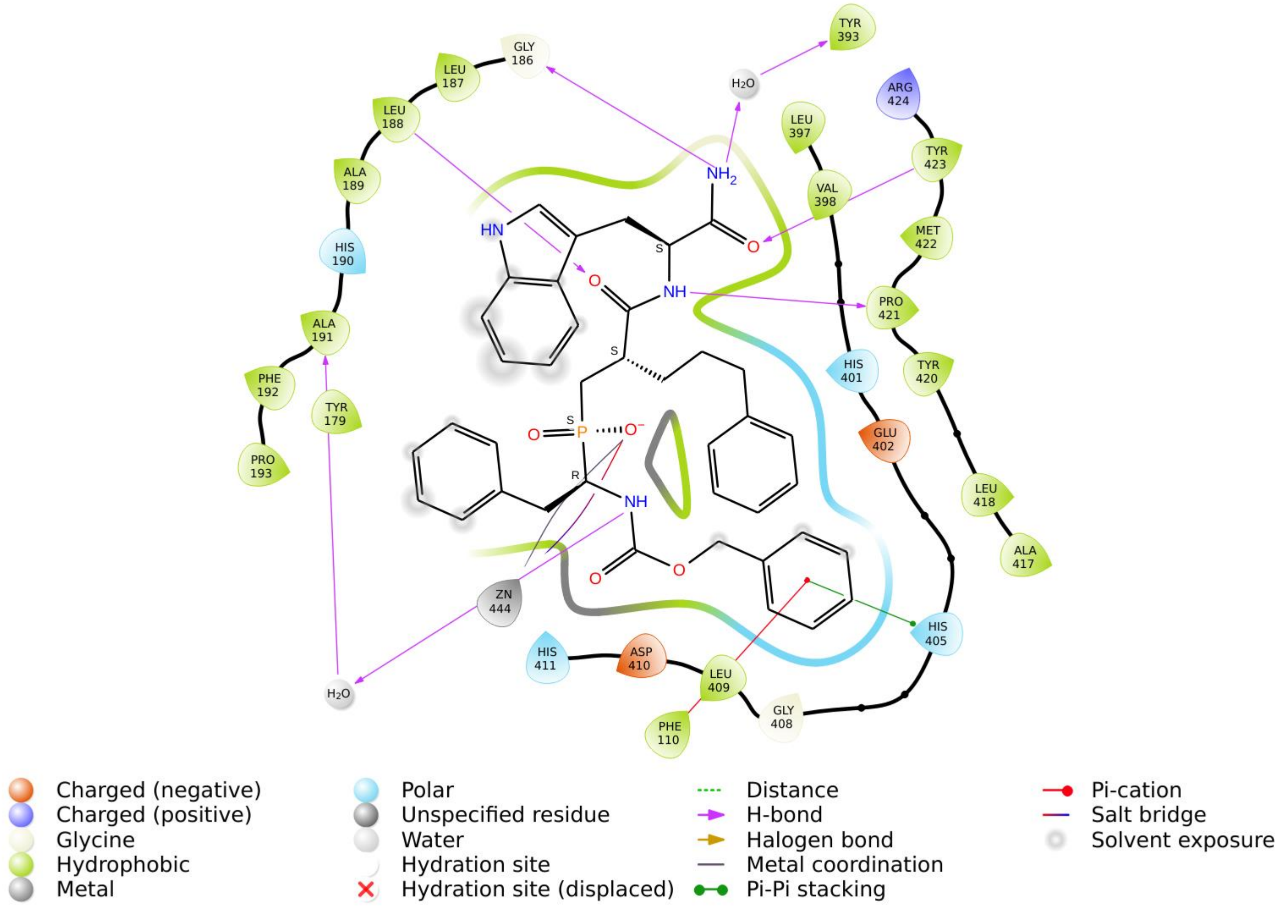

S9 Fig. **Induced fit docking of H-2 with MMP-9.** 2D illustrations of the interactions. Amino acids within 4 Å of H-2 are included.

S10 Fig

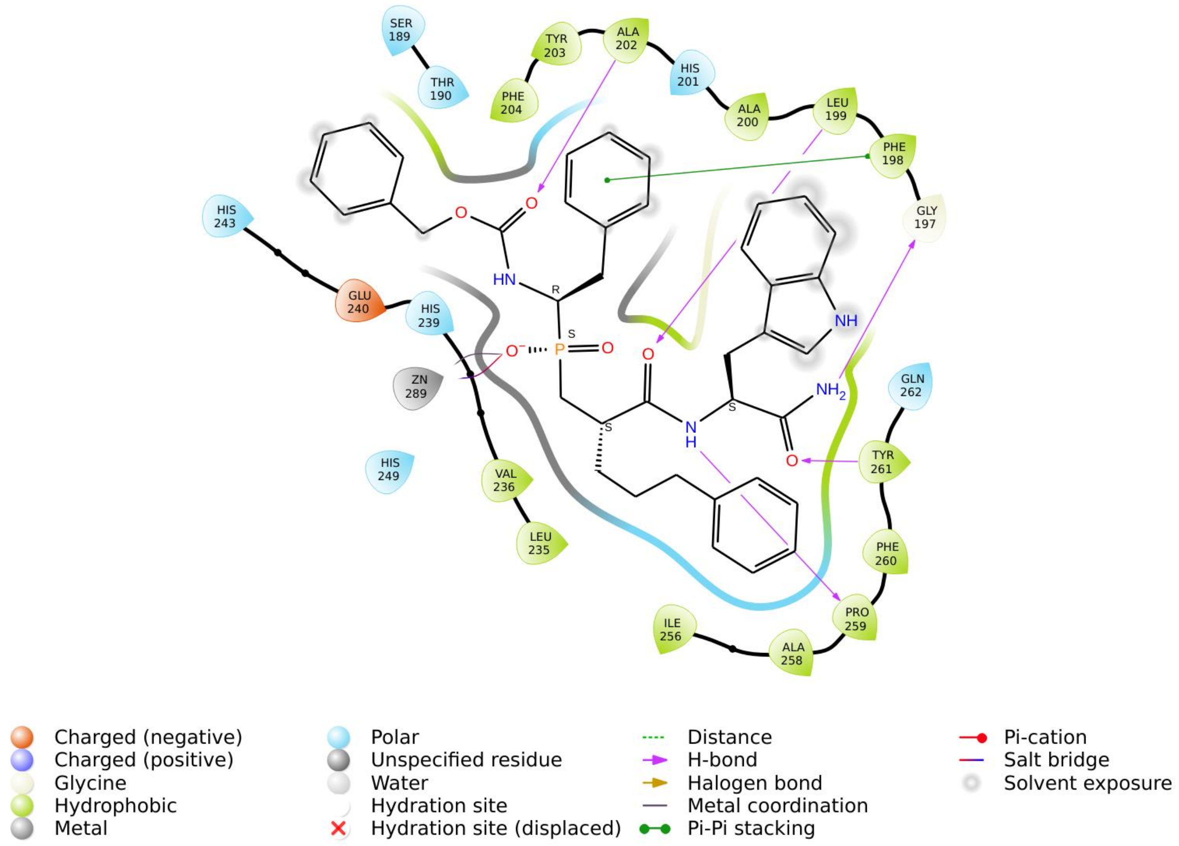

S10 Fig. **Induced fit docking of H-2 with MMP-14**. 2D illustrations of the interactions. Amino acids within 4 Å of H-2 are included.

S11 Fig

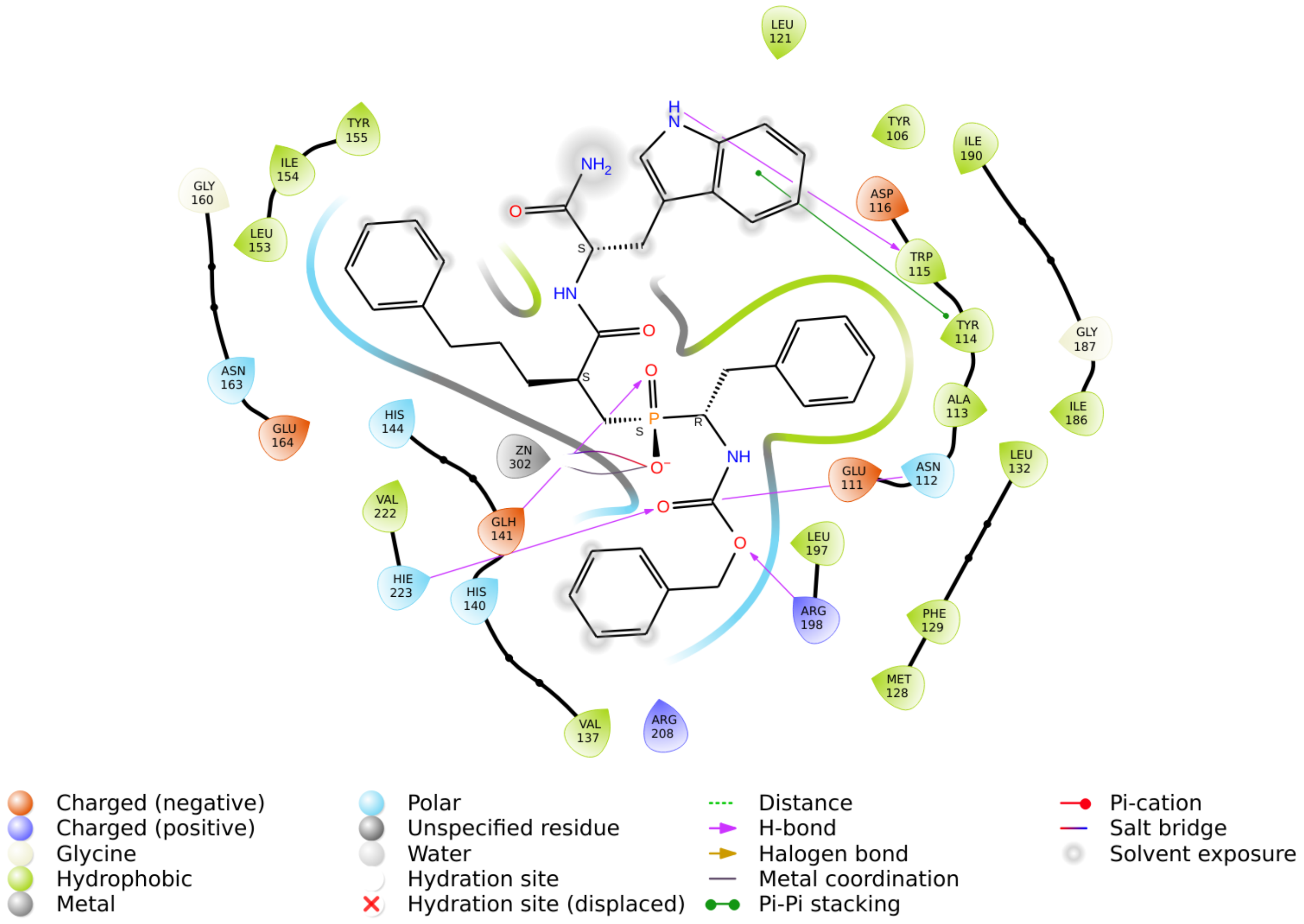

S11 Fig. **Induced fit docking of H-2 with PLN**. 2D illustrations of the interactions. Amino acids within 4 Å of H-2 are included.

S12 Fig

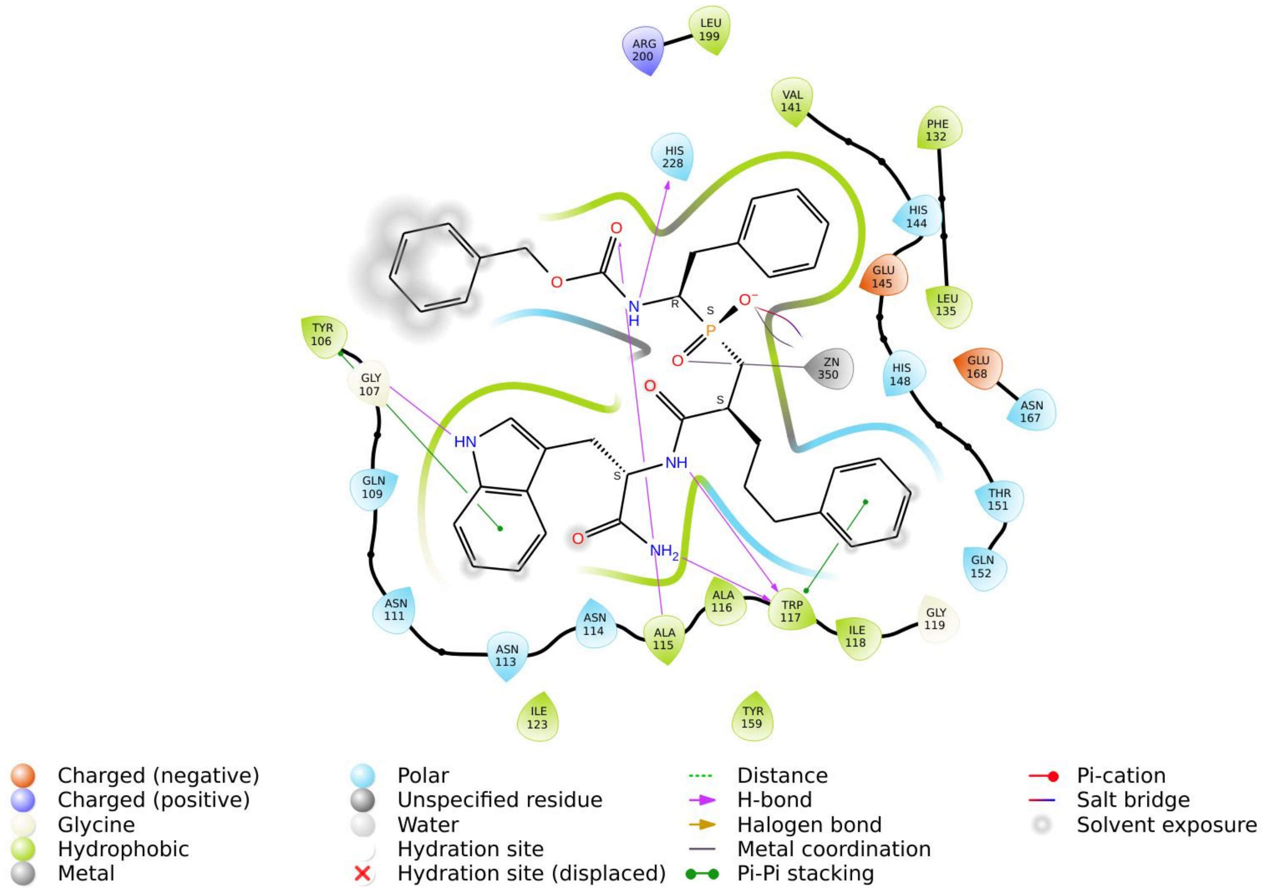

S12 Fig. Induced fit docking of H-2 with ALN. 2D illustrations of the interactions Amino acids within 4 Å of H-2 are included.

S13 Fig

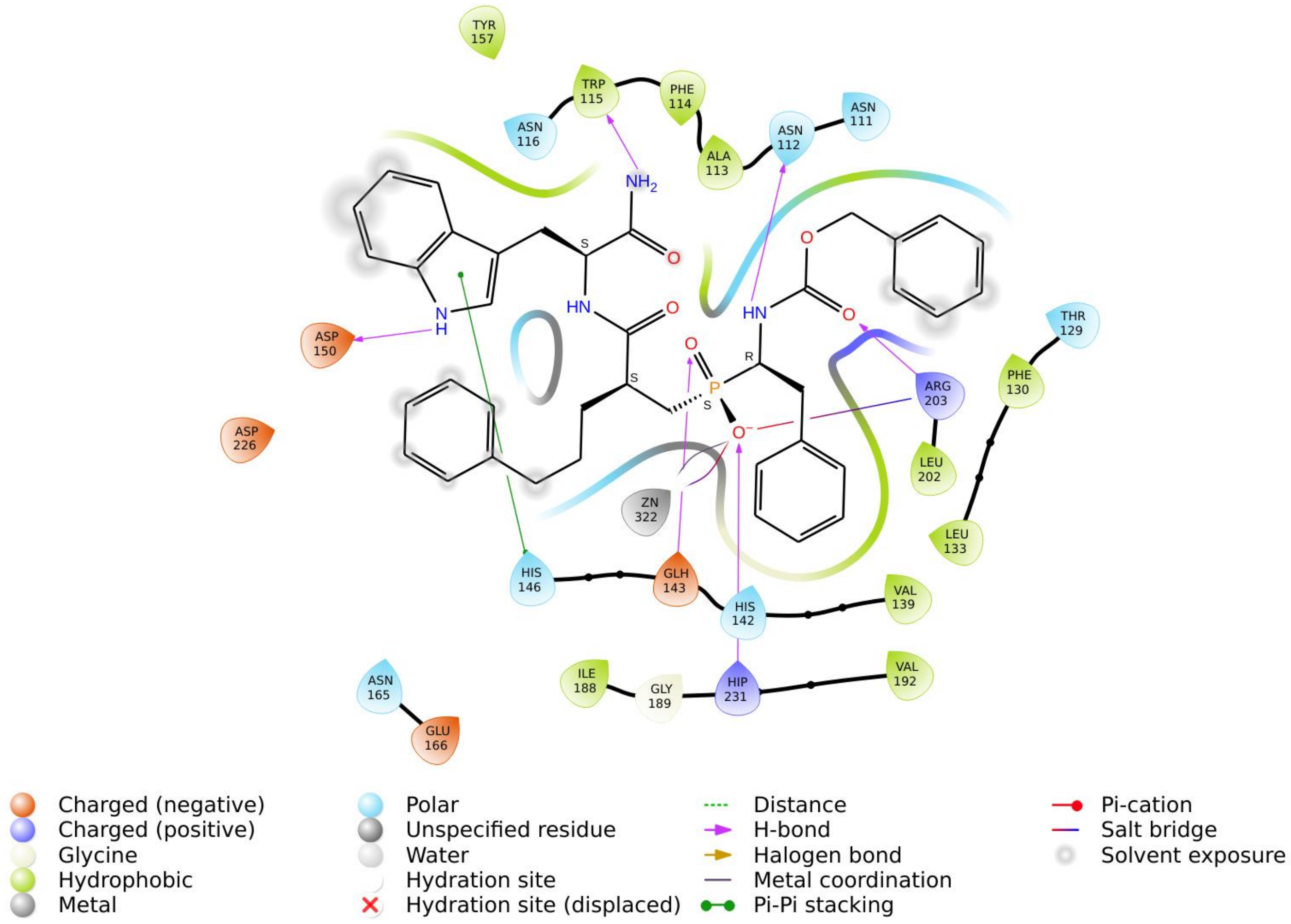

S13 Fig. **Induced fit docking of H-2 with TLN**. 2D illustrations of the interactions. Amino acids within 4 Å of H-2 are included.

S14 Fig

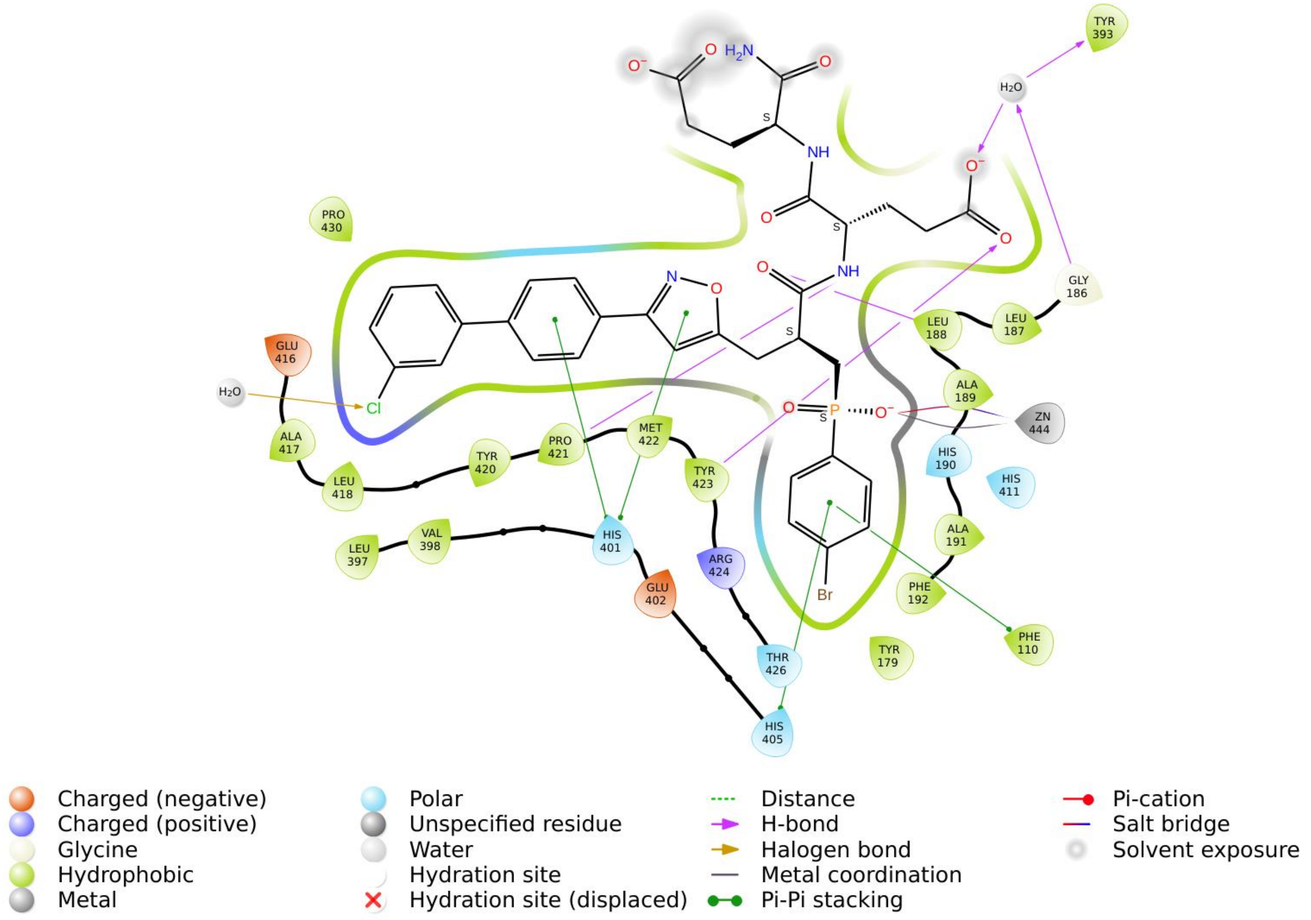

S14 Fig. Induced fit docking of H-1 with MMP-9. 2D illustrations of the interactions of the SSS-enantiomer of H-1 with amino acids in MMP-9. Amino acids within 4 Å of H-1 are included.

S15 Fig

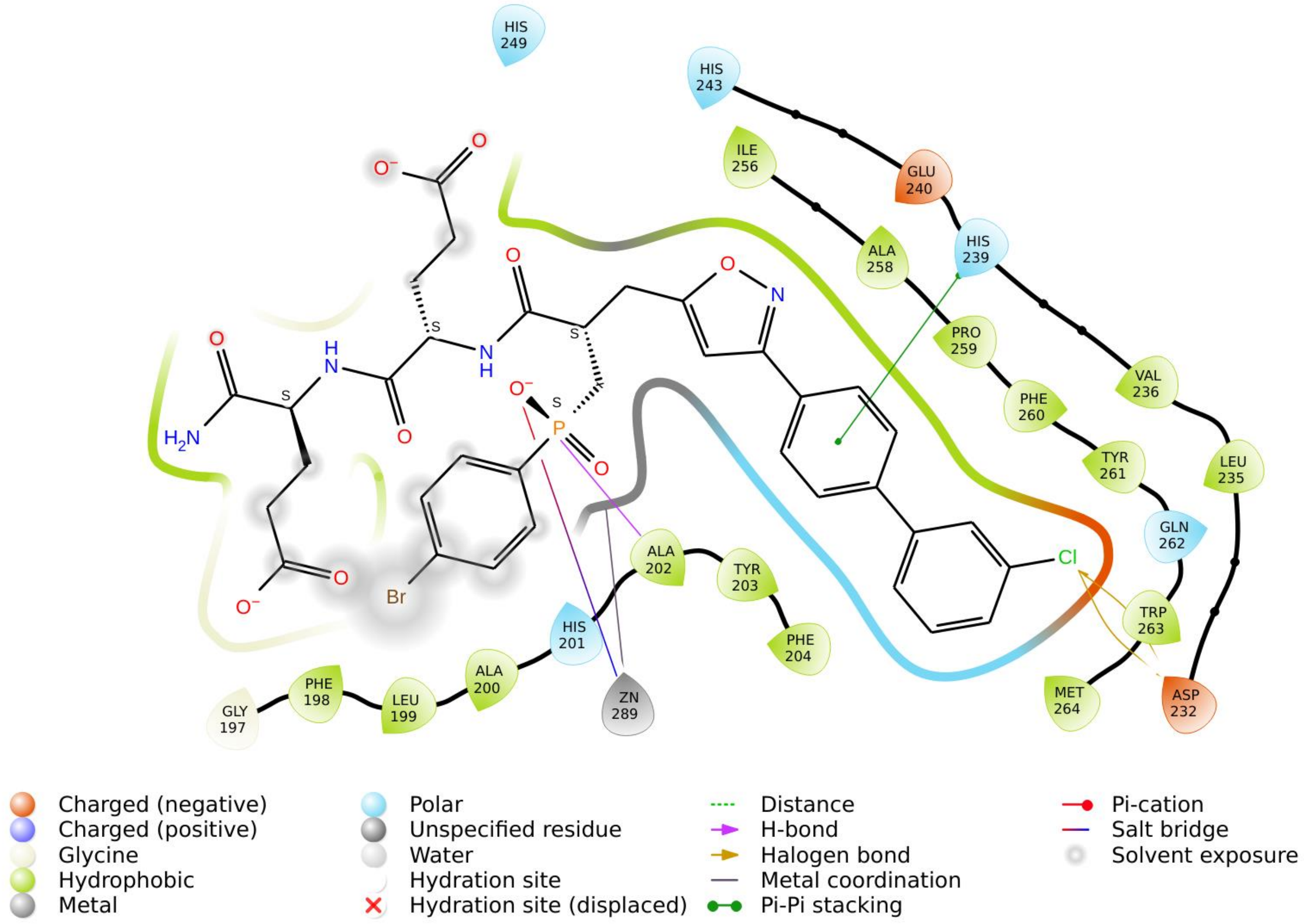

S15 Fig. **Induced fit docking of H-1 with MMP-14**. 2D illustrations of the interactions of the SSS-enantiomer of H-1 with amino acids in MMP-14. Amino acids within 4 Å of H-1 are included.
