# Supplementary figures and images for: Interactions of substrates and phosphinyl containing inhibitors with bacterial and human zinc proteases
Source: PLoS One. 2025 Aug 1;20(8):e0329362. doi: 10.1371/journal.pone.0329362 (PMC12316308; doi:10.1371/journal.pone.0329362)

## Slide 1
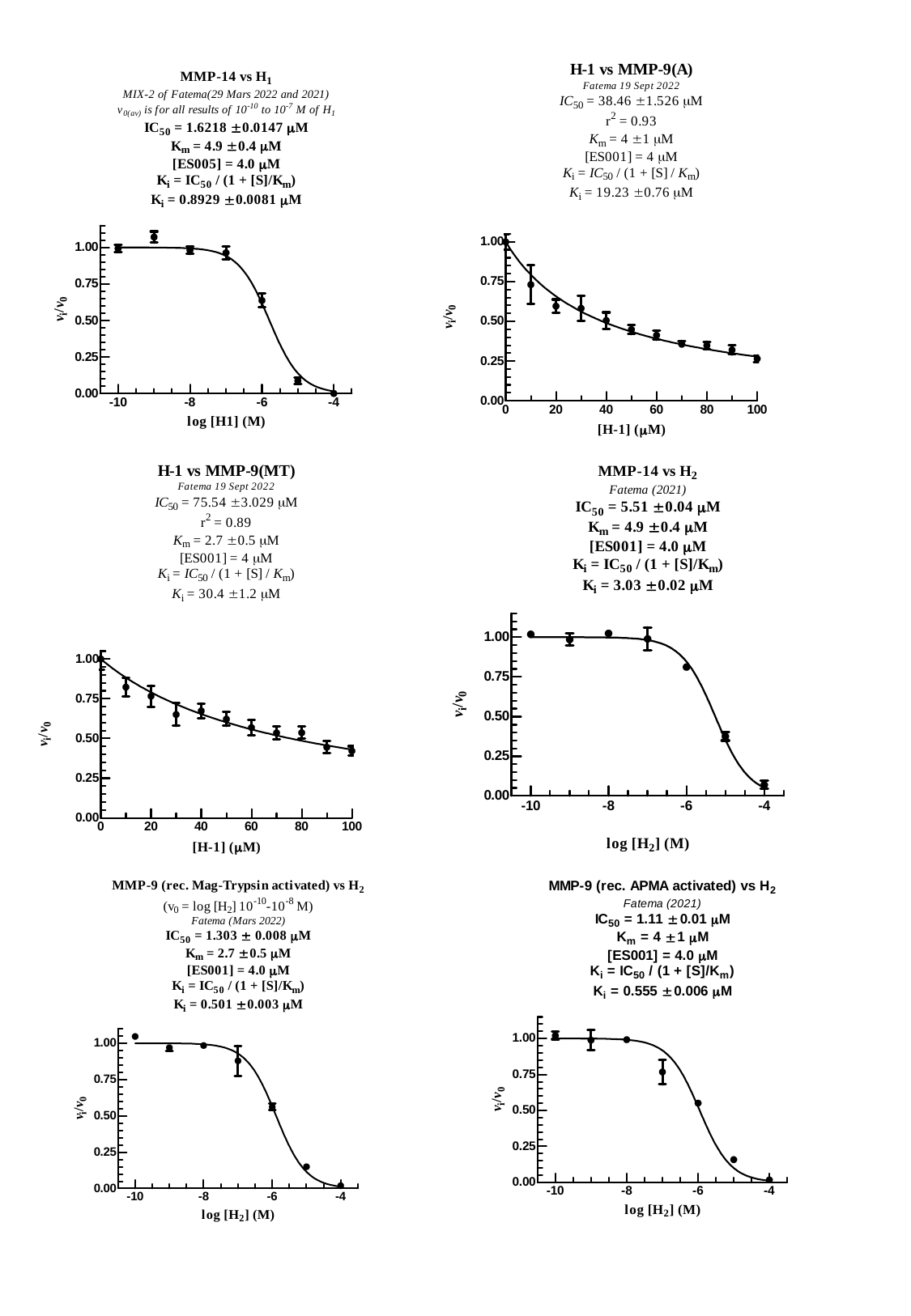

## Slide 2
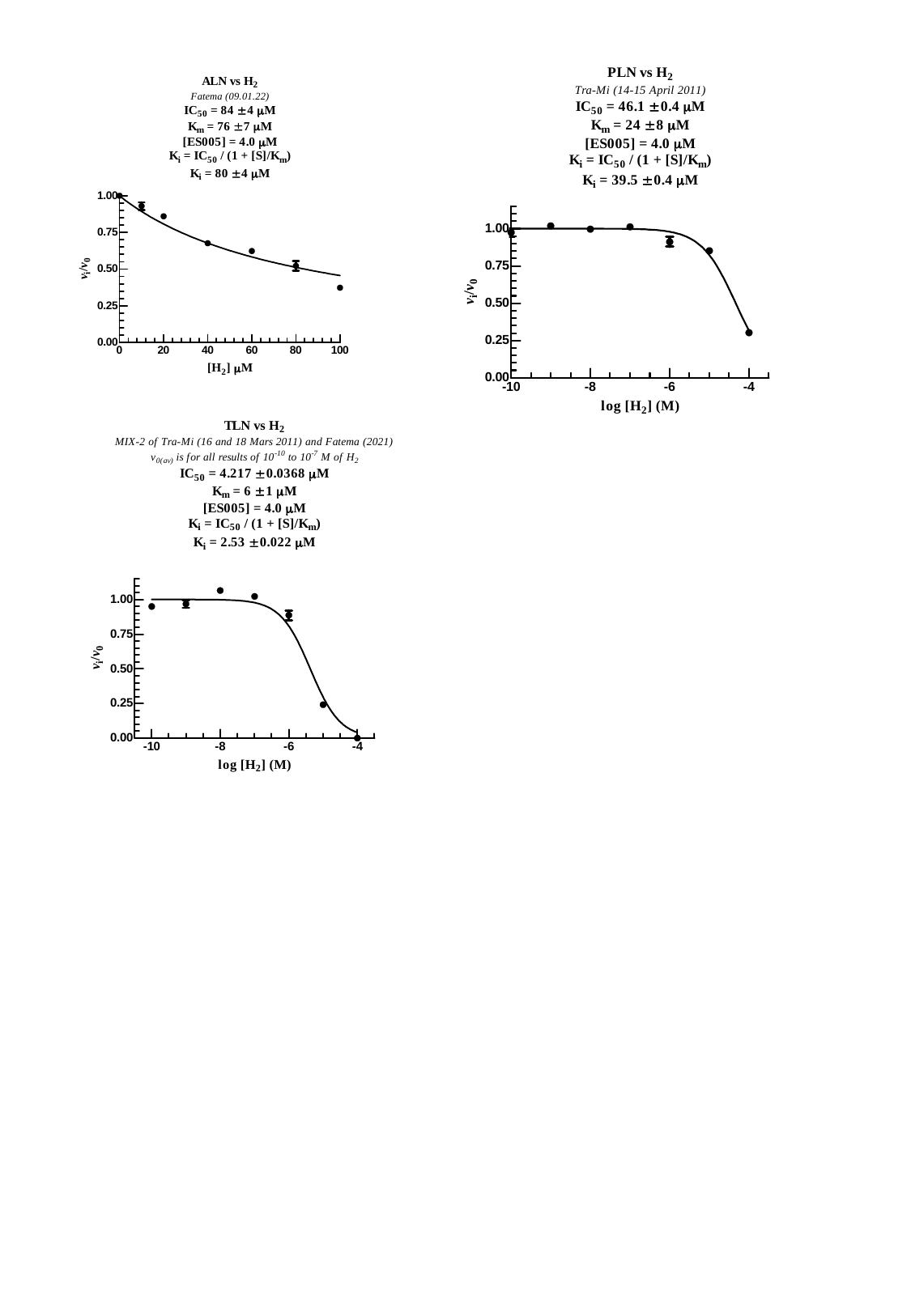

Supplement: S7 File — A PowerPoint file with enzyme kinetic data. (PPTX) [file pone.0329362.s007.pptx]

## Slide 1
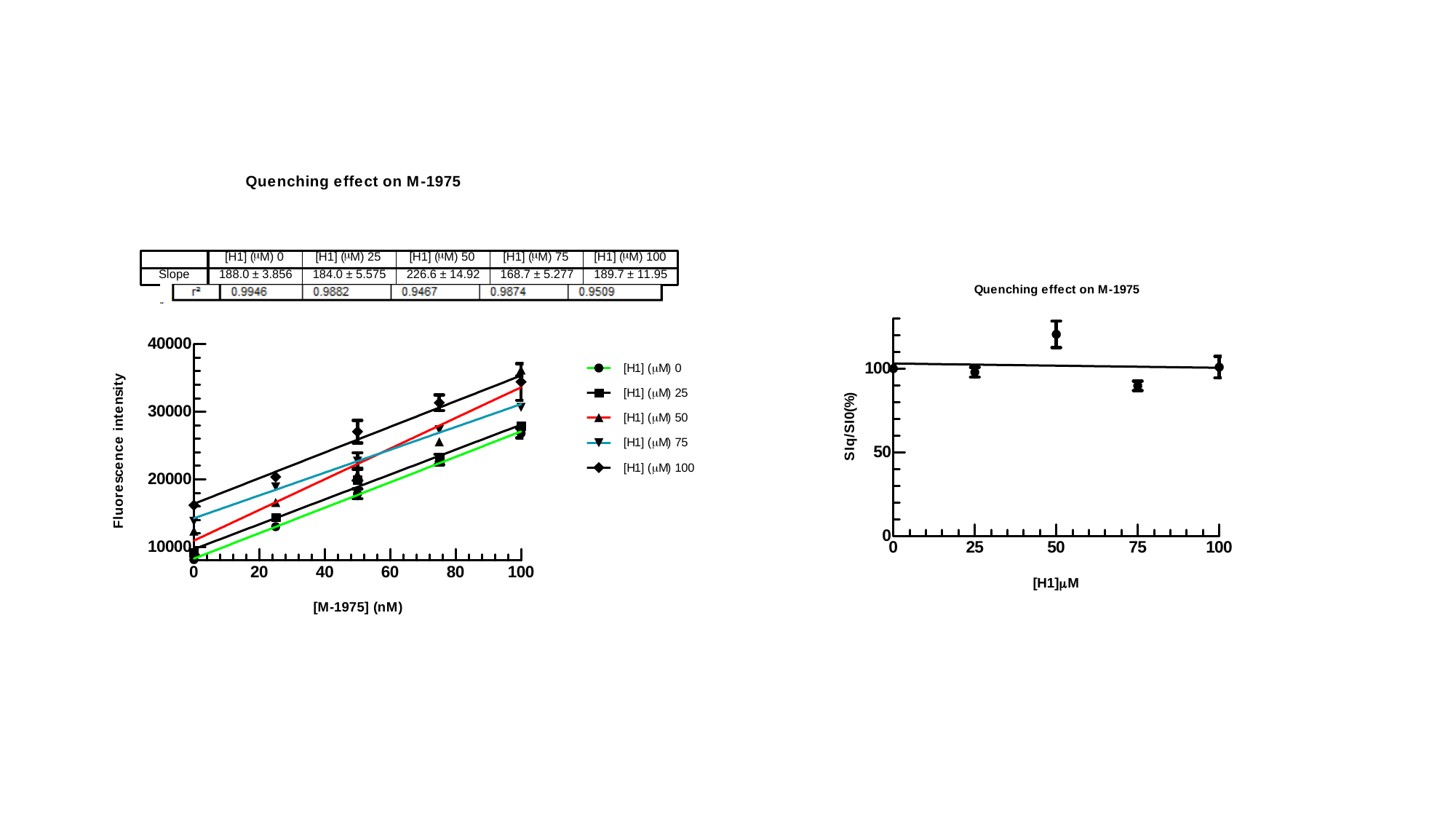

## Slide 2
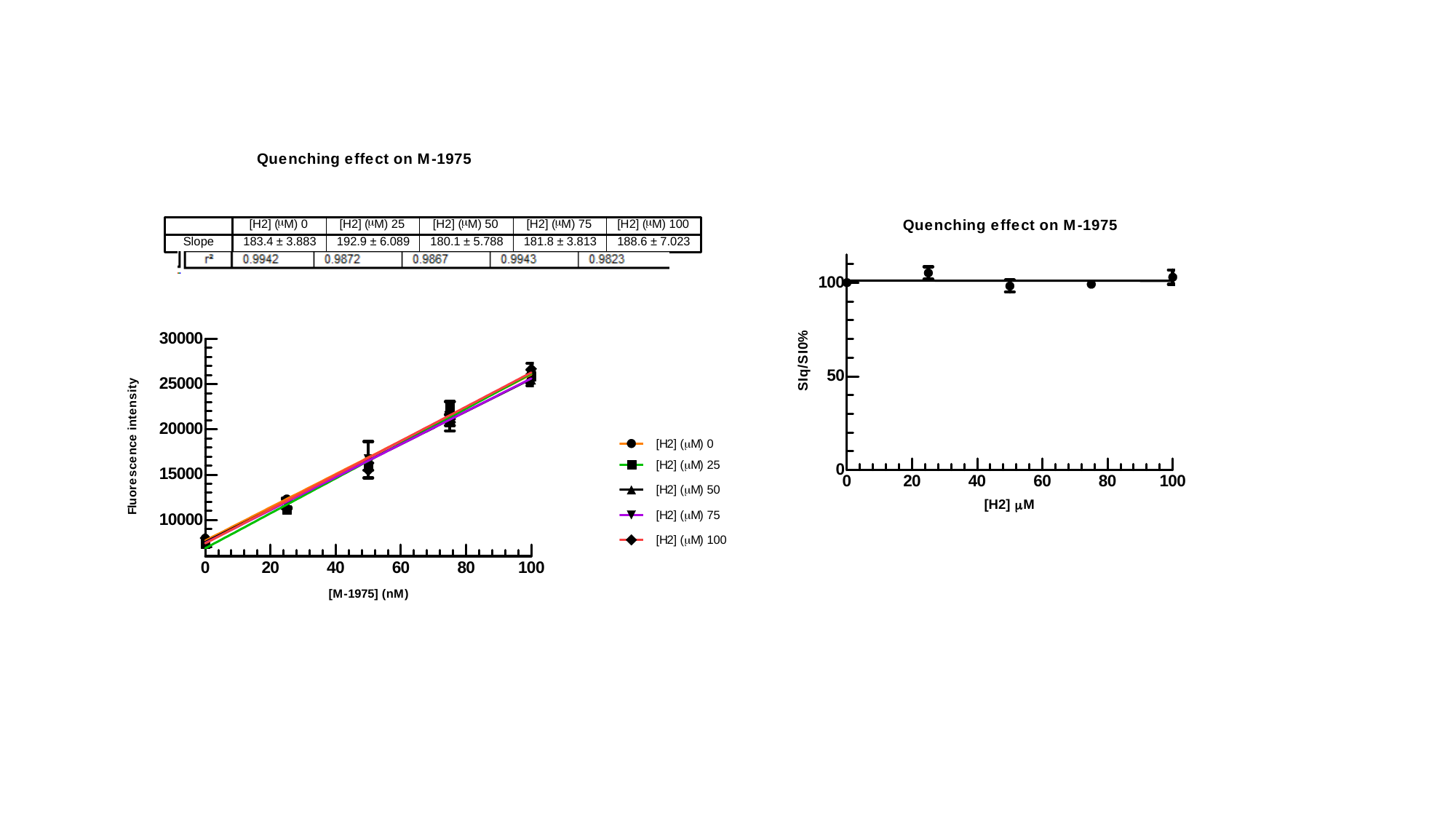

## Slide 3
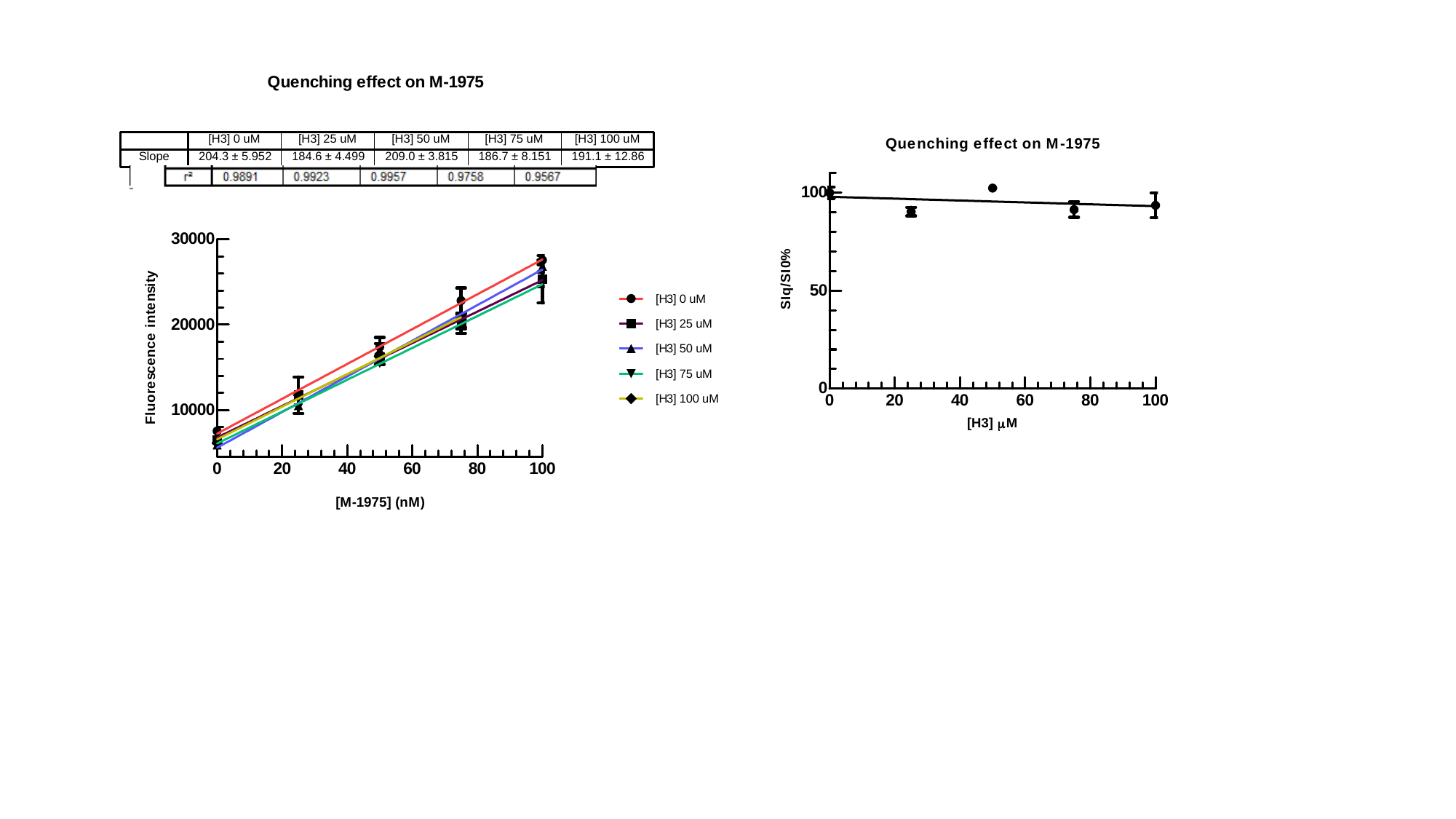

## Slide 4
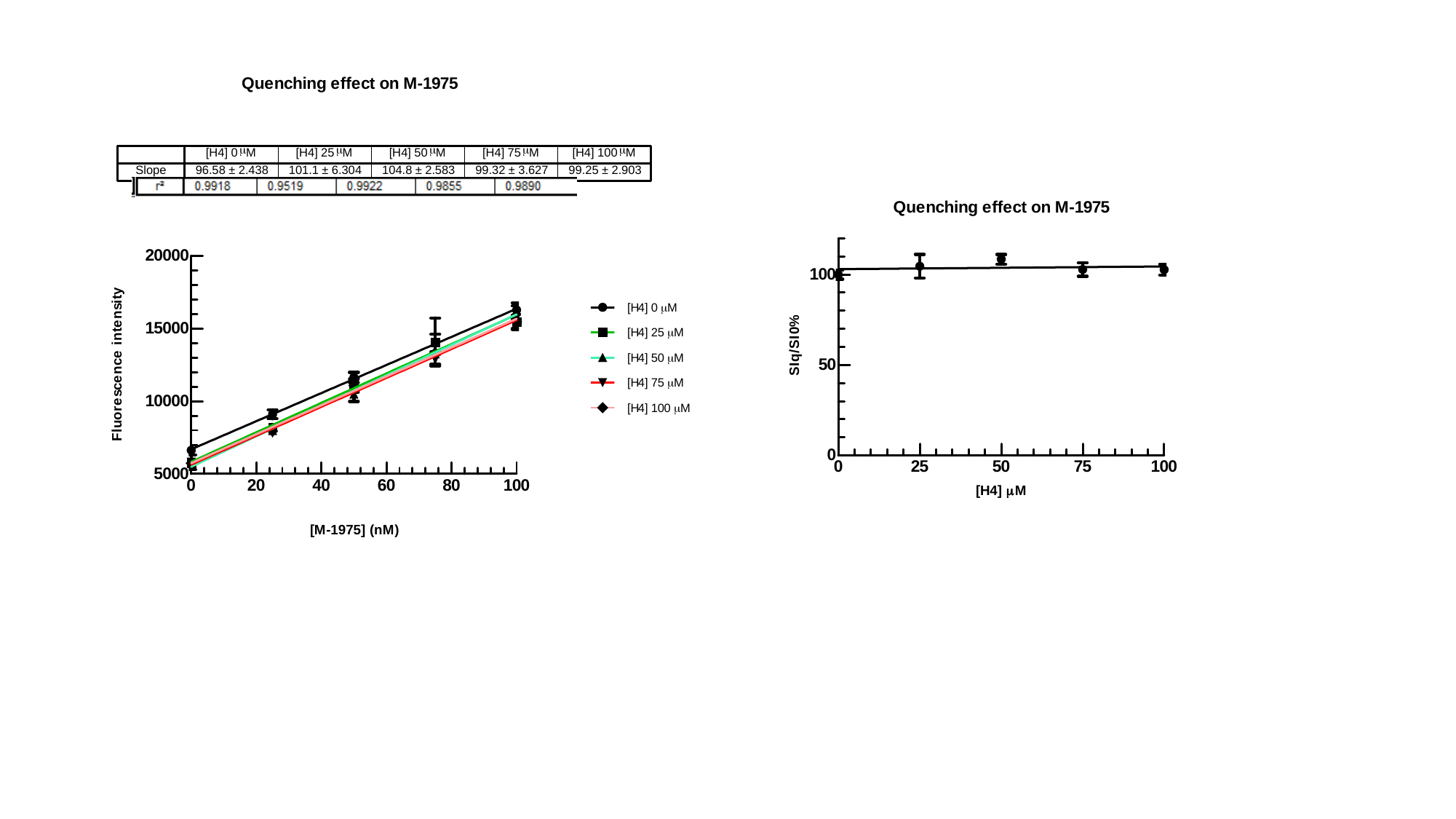

## Slide 5
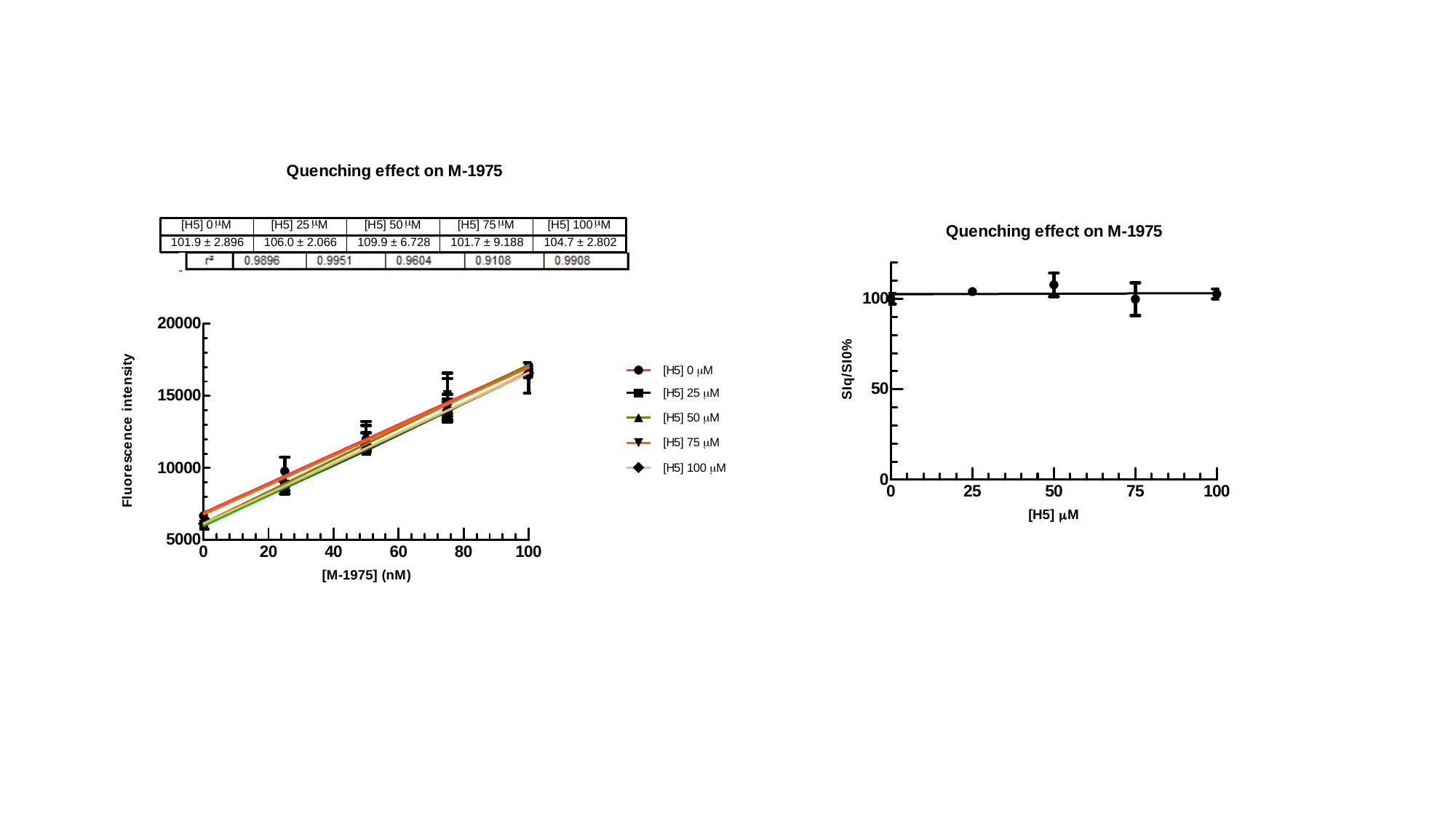

Supplement: S8 File — A PowerPoint file with enzyme kinetic data. (PPTX) [file pone.0329362.s008.pptx]
