# Supplementary material for: Interactions of substrates and phosphinyl containing inhibitors with bacterial and human zinc proteases
Source: PLoS One. 2025 Aug 1;20(8):e0329362. doi: 10.1371/journal.pone.0329362 (PMC12316308; doi:10.1371/journal.pone.0329362)
Supplement: Attachment — Submitted filename: Response to Reviewers.docx [file pone.0329362.s011.docx]

**Manuscript ID: PONE-D-25-23761**

**Changes according to requirements and comments from academic editor and reviewers**

Responses to the comments are shown in red.

**Journal Requirements**

**When submitting your revision, we need you to address these additional requirements:**

1. Please ensure that your manuscript meets PLOS ONE's style requirements, including those for file naming.

Response

We have used the template in the writing of the manuscript.

2. Please ensure that you refer to Figure 9, 10, 11 in your text as, if accepted, production will need this reference to link the reader to the figure.

Response

These figures were referred to on page 26 (as Figs 9-11) in the previous version, and the present version they are referred to on page 28 (Fig 10, Fig 11 and Fig 12 in the present version .

3. Please include a separate caption for each figure in your manuscript.

Response

Done

4. Please review your reference list to ensure that it is complete and correct. If you have cited papers that have been retracted, please include the rationale for doing so in the manuscript text, or remove these references and replace them with relevant current references. Any changes to the reference list should be mentioned in the rebuttal letter that accompanies your revised manuscript. If you need to cite a retracted article, indicate the article’s retracted status in the References list and also include a citation and full reference for the retraction notice.

Response

Compared with the previous version, five additional papers have been cited. In the reference list they have the following number: 51, 54, 55, 56 and 57. We have used the endnote style PLos in the writing of the manuscript.

**Additional Editorial Comments**

**Comments to the author**

1. Is the manuscript technically sound, and do the data support the conclusions?

The manuscript must describe a technically sound piece of scientific research with data that supports the conclusions. Experiments must have been conducted rigorously, with appropriate controls, replication, and sample sizes. The conclusions must be drawn appropriately based on the data presented.

Reviewer #1: Partly

Reviewer #2: Yes

Reviewer #3: Yes

Response

Methodical information suggested by reviewer 1 has been added. Please see the responses to reviewer 1.

4. Is the manuscript presented in an intelligible fashion and written in standard English?

PLOS ONE does not copyedit accepted manuscripts, so the language in submitted articles must be clear, correct, and unambiguous. Any typographical or grammatical errors should be corrected at revision, so please note any specific errors here.

Reviewer #1: Yes

Reviewer #2: No

Reviewer #3: Yes

Response

Typographical and grammatical errors have been corrected.

**5. Review Comments to the Author**

**Reviewer #1:**

1. The abstract effectively summarizes the experimental and computational results, but it lacks a clear statement of the study’s objectives and significance. For example, explicitly state why comparing bacterial vs. human zinc proteases with these inhibitors is important.

Response

The abstract has been rewritten, and the content is more clearly separated into background, methods, results and conclusions.

2. The introduction covers relevant background on metalloproteases and zinc-binding groups, However the scientific gap and the motivation for the study still need attention. For example, explain why it is important to study both bacterial (“thermolysin-like”) and human (MMP) zinc proteases together, and why phosphinyl inhibitors are of particular interest.

Response

The following is added/rewritten in the introduction: “*However, X-ray crystallography studies show that compounds with the phosphinyl group may act as transition state analogues, enabling the connecting scaffold to interact with both primed and unprimed subsites of the active site. Such binding modes may potentially enhance the selectivity compared with inhibitors targeting primed or unprimed subsites, only [14, 16, 31-34]*”.

The following is added/rewritten in the introduction: *“Inhibitors of bacterial virulence is suggested to be a promising strategy in the fight against bacterial resistance. However, inhibitors should not interfere strongly with human targets to be promising drug candidates. Human MMPs resemble the bacterial virulence factors of the M4 family in both structure and function.”*

3. The introduction also mention “novel substrate ES005” without much explanation. It would help to briefly note why ES005 was chosen and what is expected, addressing what particular group in this substrate are of interest. Authors can follow <https://pubs.rsc.org/en/content/articlelanding/2022/nj/d2nj02482a> for stating why paerticular substrate/compound is needed and what could be expected from this.

Response

An extra paragraph has been added in the beginning of the results and discussion section under the heading “Enzyme activity of the five MPs against ES001 and ES005” explaining why we have used ES001 and ES005 as substrates.

4. The methods are detailed, but the number of experimental replicates and statistical treatment should be specified. For example, for enzyme assays (Km and Ki determinations): how many independent experiments were performed and how error was estimated. Also for molecular modelling authors have not mentioned the grid size of the active site. Authors can follow <https://onlinelibrary.wiley.com/doi/full/10.1002/jmv.29594> to improve the material and method section for molecular modelling.

Response

**Enzyme assays**

The following sentence has been added in the Material and Methods section under the heading “Determination of *K_m_* values”: *“The K_m_ and s.d values were calculated with Graph Pad Prism 5 using the Michaelis-Mention equation.*”

Under the heading “Determination of *IC*_50_ and *K*_i_ values” in the Material and Methods section, the sentence on lines 189-190 in the previous version has been rewritten to: “*The IC_50_ values with corresponding s.d. were calculated with GraphPad Prism 5 using equation 1 or 2, depending on the concentration range of the phosphinic compounds”*.

The legends to Fig. 7 (Fig. 6 in the initial version) have been changed to:

“***Fig. 7. Inhibitory effects.*** *The inhibitory effects of 100 μM of the H-compounds on the enzymatic activity of MMP-14, MMP-9(MT), MMP-9(A), TLN, PLN and ALN. All compounds showed fast inhibitory binding against all enzymes, except for H-3 against MMP-14. For the fast inhibitory binding, the* *v_i_/v_0_ (mean ± s.d.) was based on the mean values of 0-30 min pre-incubation. For H-3 against MMP-14, the v_i_/v_0_ (mean ± s.d.) was based on the mean values of 15-30 min pre-incubation, i.e. where the time curve in the S8 Fig flattens out.”*

In the Material and Methods section under the heading “Determination of IC_50_ and K_i_ values”, it is already stated that all experiments were performed at least in triplicate. In the legends to supplementary figure S8 (time dependent inhibition) it is stated that the v_i_/v*_0_* results are based on 3-7 experiments.

**Molecular modelling**

The description of the induced fit docking has been rewritten. An atomic distance of 12 Å from the centre of the co-crystallized inhibitors was used to generate the 3D grid box for docking giving a grid box of 24 Å x 24 Å x 24 Å. A sentence in the material and methods section (Induced fit docking of H-1 and H-2) has been rewritten to: “*An atomic distance of 12 Å from the centre of the co-crystallized inhibitors was used to generate the 3D grid box for docking giving a grid box volume of 24 Å x 24 Å x 24 Å.*”

In addition, the following is added under the same heading: “*During induced fit docking, the ring conformations were sampled in an energy window of 2.5 kcal/mol. Non-planar conformations for amide bonds were penalized. The enzyme and the ligands Van der Waals scaling was set to 0.50. Residues within 5.0 Å of ligand poses were refined, and side chains were optimized.*”

5. The term “residual interaction fraction” is introduced in the MD discussion but not explained. Either define this metric (percentage of time a bond is present?). Readers unfamiliar with this phrasing may find it confusing.

Response

Protein interactions with the ligand can be monitored throughout an MD simulation. These interactions can be categorized by type and summarized. Protein-ligand interactions (or 'contacts') are categorized into four types: hydrogen bonds, hydrophobic, ionic and water bridges. One protein residue (amino acid) may have several contacts for the same type of interaction, but also several types of contacts with the ligand during MD. Therefore, it is possible with values over 1.0 for the residual interaction fraction.

An explanation of the residual interaction fraction is given in the results and discussion section under the heading “Interactions of ES001 with MMP-9 and MMP-14” in the second paragraph after Fig. 6.

6. The Conclusions summarize the findings well, but need to improve. For instance, explicitly mention how these results advance the understanding of inhibitor selectivity or could guide the design of new antibacterials. Right now the conclusion is mostly a recapitulation of results. Highlighting one or two key takeaways for future work would strengthen it.

Response

An extra paragraph has been added to the conclusion with suggestions for further design of inhibitors with higher selectivity.

Minor Comments

a. "we have not demonstrated that bacterial en zymes prefer ES005 over ES001 and that human MMPs favour ES001 over ES005” is somewhat confusing. If prior studies never directly compared the two substrates, rephrase more clearly.

Response

An extra paragraph has been added to the first part of the results and discussion section. Please also see response to comment 3.

b. Some sentences are lengthy and could be split or rephrased.

Response

In the adjusted version of the manuscript, sentences have been split and rephrased.

c. The conclusion states that H-3 to H-5 had Ki values in the “high range.” It would be clearer to say explicitly that “H-3, H-4, and H-5 did not show appreciable inhibition (Ki >100 μM) for any enzyme.

Response

We have not written anything like that in the conclusions. What is written in the Results and discussion section is the following: “Among the tested compounds, only H-2 showed strong inhibition across all five proteases, with the most potent effects observed against MMP-9 and MMP-14 (Fig 6). In contrast, H-1 exhibited strong inhibition exclusively against the two human proteases, while H-3, H-4, and H-5 showed weak or no inhibition of the enzymes.”

**Reviewer #2:**

1. What is the rationale behind selecting ES001 and ES005 over other available substrates?

Response

An extra paragraph has been added to the beginning of the results and discussion section under the heading “Enzyme activity of the five MPs against ES001 and ES005” explaining why we have used ES001 and ES005 as substrates.

2. For the MD simulations, please report the pressure used for the equilibrium.

Response

The treatment of pressure during equilibrium has been added to the methods section under the heading “Molecular dynamics simulations”

3. It would help the reader to visualize the data better if S2 Fig is part of the main text of the manuscript.

Response

S2 Fig has been moved to the main text. Fig. 4 in the present version of the manuscript.

4.Some supplemental figure numbers referred to in the main text are incorrect. Please check the numbers.

Response

The referred figure numbers have been checked and updated.

5. Please provide the PDB ID of the MMP-9 mutant used in the MD simulations (page 17).

Response

The PDB ID has been inserted (4JIJ) as suggested by the reviewer. The PDB ID is still also given in the methods section and in the legends to Fig. 2.

6. The last two sentences of the sub-pocket amino acids paragraph was hard to follow. Please consider adding a comparison of the data from Rahman et al. 2021 to the data reported in Table 3.

Response

The last part of this section has been rewritten and includes some comparison of the table in the present study with the table in the previous study.

7. Please label Fig 7A highlighting the key residues and the sub-pockets. For Fig 7A and 7B, a surface image of the protein complex with labeled sub-pockets would help to visualize and understand the results.

Response

The figure has been correct according to the comment. Fig. 8 in the present version of the manuscript. The figure now consists of three panels. The extra panel is showing the different subpockets.

**Reviewer #3:**

1. The authors used induced fit docking for H-1 and H-2, no comparison with crystallographic data (where available) or experimental validation (e.g., SAR) is provided to confirm pose accuracy.

Response

Comparison with X-ray crystallographic data has been done. In the section “Inhibitory effects and binding modes of the phosphinic compounds” the docked complexes the RSS enantiomer of H-2 in MMP-9 and MMP-14 were compared with the X-ray structure of the SSS enantiomer with MMP-11 (PDB id: 1HV5). In addition, the docked complexes of the RSS and SSS enantiomers of H-1 into both MMP-9 and MMP-14 were compared with the X-ray structure of the SSS enantiomer of H-1 with MMP-12 (PDB id: 5CZM). In the results and discussion section, we also try to explain the obtained experimental values using docking. However, only 5 compounds were tested and only one of them inhibited the bacterial zinc MPs.

2.Please label measurements for the key interactions (bong length).

Response

We decided not to include atomic bond lengths in our illustrations. The presence of different interactions during MDs with the substrates are shown in Figs 5 and 6, and in Supporting Information. Figs 8-15 show the amino acids closest to H-1 and H-2 in the docked complexes, and 2D illustrations in the Supporting Information (S9 Fig - S15 Fig) show the non-bonded interactions of H-1 and H-2 with the enzymes. In addition, the coordination between zinc and amino acids in the enzymes are indicated in the docking illustrations. Therefore, the figures give a lot of information, including enzyme side chains, that already complicate the interpretation for the reader. Including bond lengths of key interactions in addition (e.g distances between phosphinyl oxygens and zinc), will complicate it even more.

3.Only five phosphinyl-containing inhibitors were tested. The conclusions regarding selectivity would be stronger with a broader panel of inhibitors (even with computational predictions).

Response

We fully agree that the selectivity conclusions would have been stronger if additional compounds were tested. However, we only had 5 phsophinyl containing compounds for testing in the present study, and one of them inhibited the virulence factors. Based on only the results in the present study we cannot draw any clear conclusions. Our conclusions regarding selectivity are therefore supplemented with experiences from our previous studies that are cited.

4. Authors should provide molecular weights and structural notations for clarity.

Response

Structural figures of the phosphinyl containg compounds (Fig. 1) and of the substrates (Fig. 2) are already included in the manuscript. In addition, the structure of H-1, H-2, ES001 and ES005 are also shown in complexes with the enzymes.

5. Authors are suggested to rewrite the abstract for more clarity e.g seprate out methods, results and conclusion.

Response

The abstract has been rewritten. Background, methods, results and conclusion are more clearly separated than in the previous version.

Additional changes:

1. Changes were also made according to the suggestions in the pdf file (PONE-D-25-23761_with reviewer comments) that was enclosed with the email from the academic editor.
2. The reference list was updated with five references. References number 51, 54, 55, 56 and 57 in the reference list.
