# Supplementary material for: Interactions of substrates and phosphinyl containing inhibitors with bacterial and human zinc proteases
Source: PLoS One. 2025 Aug 1;20(8):e0329362. doi: 10.1371/journal.pone.0329362 (PMC12316308; doi:10.1371/journal.pone.0329362)
Supplement: Attachment — Submitted filename: PONE-D-25-23761_with reviewer comments - 5-21-2025.pdf [file pone.0329362.s009.pdf]

PLOS ONE

Interactions of substrates and phosphinyl containing inhibitors with bacterial and human and zinc proteases

--Manuscript Draft--

**Manuscript Number:**	PONE-D-25-23761
**Article Type:**	Research Article
**Full Title:**	Interactions of substrates and phosphinyl containing inhibitors with bacterial and human and zinc proteases
**Short Title:**	Interactions of substrates and inhibitors with zinc proteases
**Corresponding Author:**	Ingebrigt Sylte<br>UiT The Arctic University of Norway: UiT Norges arktiske universitet<br>Tromsø, Troms NORWAY
**Keywords:**	zinc metalloproteases, bacterial virulence factors, Pseudomonas aeruginosa, Staphylococcus aureus, human matrix metalloproteases, substrates, MALDI-TOF MS, cleavage sites, molecular dynamics simulations, inhibitors, enzyme inhibition kinetics, induced fit
**Abstract:**	The bacterial virulence factors thermolysin (TLN), pseudolysin (PLN, LasB) and aureolysin (ALN) are zinc metalloproteases. In the present study, the molecular interactions and cleavage of TLN with the substrate Mca-Arg-Pro-Pro-Gly-Phe-Ser-Ala-Phe-Lys(Dnp)-OH (ES005) were studied by matrix-assisted laser desorption ionization time-of-flight mass spectrometry (MALDI-TOF MS) and molecular dynamics (MD) simulations. In addition, enzyme inhibition kinetic studies were used to test 5 compounds containing phosphinyl as the zinc binding group for their inhibition of TLN, PLN and ALN using ES005 as substrate, and of the human matrix metalloproteases MMP-9 and MMP-14 using Mca-Pro-Leu-Gly-Leu-Dpa-Ala-Arg-NH2 (ES001) as substrate. The MALDI-TOF MS results revealed that TLN cleaves ES005 at three sites. The most abundant cleavages were between Ala and Phe, and between Gly and Phe, while the third was between Ser and Ala. MD simulations with Ala or Gly in the S1 subpocket and Phe in S1' subpocket gave stable interactions between ES005 and TLN. However, the MD simulations with Ser in the S1 subpocket and Ala in the S1' subpocket showed larger movements of ES005 relative to the catalytic site than the others, which may explain that products from the Ser-Ala cleavage were less abundant than from Ala-Phe and Gly-Phe. Compound H-1 inhibited MMP-14 and two different forms of MMP-9 with inhibition constants ranging from 0.89 to 30 µM but did not inhibit the bacterial enzymes. Induced fit docking indicated that the aromatic group of H-1, that entered the S1' subpocket of the MMPs, is too big for the S1' subpocket of the bacterial virulence factors. Compound H-2 inhibited the MMPs with inhibition constants ranging from 0.53 µM (MMP-9) to 3.0 µM (MMP-14) and the bacterial enzymes with inhibition constants ranging from 2.5 µM (TLN) to 80 µM (ALN). Induced fit docking indicated that H-2 bound quite differently to the human and bacterial zinc proteases.
**Order of Authors:**	Ingebrigt Sylte
	Fatema Amatur Rahman, PhD
	Ida Kristine Østnes Hansen, PhD
	Imin Wushur, PhD
	Tor Haug, PhD
	Klara Stensvåg, PhD
	Bibek Chaulagain, Master of Biomedicine
	Tra-Mi Nguyen, Master of Pharmacy
	Olayiwola Adedotun Adekoya, PhD
	Nabin Malla, PhD
	Jan-Olof Winberg, PhD

Powered by Editorial Manager® and ProduXion Manager® from Aries Systems Corporation

Additional Information:	
**Question**	**Response**
**Financial Disclosure**<br><br>Enter a financial disclosure statement that describes the sources of funding for the work included in this submission. Review the submission guidelines for detailed requirements. View published research articles from *PLOS ONE* for specific examples.<br><br>This statement is required for submission and **will appear in the published article** if the submission is accepted. Please make sure it is accurate.<br><br>**Funded studies**<br>Enter a statement with the following details:<br>• Initials of the authors who received each award<br>• Grant numbers awarded to each author<br>• The full name of each funder<br>• URL of each funder website<br>• Did the sponsors or funders play any role in the study design, data collection and analysis, decision to publish, or preparation of the manuscript?<br><br>Did you receive funding for this work?	Yes
Please add funding details.<br>as follow-up to "**Financial Disclosure**<br><br>Enter a financial disclosure statement that describes the sources of funding for the work included in this submission. Review the submission guidelines for detailed requirements. View published research articles from *PLOS ONE* for specific examples.<br><br>This statement is required for submission and **will appear in the published article** if the submission is accepted. Please make sure it is accurate.	IS<br>grant no: 1514-20,<br>Funder: Health Authorities of Northern Norway (Helse Nord)<br>URL: https://www.helse-nord.no/<br>The grant financed the experimental studies.<br><br>FAR: PhD grant from UiT The Arctic University of Norway<br><br>TH, KS, JOW. IS: Running costs from UiT The Arctic University of Norway

**Funded studies**<br>Enter a statement with the following details:<br>• Initials of the authors who received each award<br>• Grant numbers awarded to each author<br>• The full name of each funder<br>• URL of each funder website<br>• Did the sponsors or funders play any role in the study design, data collection and analysis, decision to publish, or preparation of the manuscript?<br><br>Did you receive funding for this work?"	
Please select the country of your main research funder (please select carefully as in some cases this is used in fee calculation).<br>as follow-up to "**Financial Disclosure**<br><br>Enter a financial disclosure statement that describes the sources of funding for the work included in this submission. Review the submission guidelines for detailed requirements. View published research articles from *PLOS ONE* for specific examples.<br><br>This statement is required for submission and **will appear in the published article** if the submission is accepted. Please make sure it is accurate.<br><br>**Funded studies**<br>Enter a statement with the following details:<br>• Initials of the authors who received each award<br>• Grant numbers awarded to each author<br>• The full name of each funder<br>• URL of each funder website<br>• Did the sponsors or funders play any role in the study design, data collection and analysis, decision to publish, or preparation of the manuscript?<br><br>Did you receive funding for this work?"	NORWAY - NO
**Competing Interests**	The authors have declared that no competing interests exist

Use the instructions below to enter a competing interest statement for this submission. On behalf of all authors, disclose any competing interests that could be perceived to bias this work—acknowledging all financial support and any other relevant financial or non-financial competing interests.<br><br>This statement is required for submission and **will appear in the published article** if the submission is accepted. Please make sure it is accurate and that any funding sources listed in your Funding Information later in the submission form are also declared in your Financial Disclosure statement.<br><br>View published research articles from *PLOS ONE* for specific examples.<br><br>**NO authors have competing interests**<br><br>Enter: *The authors have declared that no competing interests exist.*<br><br>**Authors with competing interests**<br><br>Enter competing interest details beginning with this statement:<br><br>*I have read the journal's policy and the authors of this manuscript have the following competing interests: [insert competing interests here]*<br><br>* typeset	
**Ethics Statement**<br><br>Enter an ethics statement for this submission. This statement is required if the study involved:<br><br>• Human participants<br>• Human specimens or tissue<br>• Vertebrate animals or cephalopods<br>• Vertebrate embryos or tissues	N/A

Powered by Editorial Manager® and ProduXion Manager® from Aries Systems Corporation

• Field research<br><br>Write "N/A" if the submission does not require an ethics statement.<br><br>General guidance is provided below. Consult the submission guidelines for detailed instructions. **Make sure that all information entered here is included in the Methods section of the manuscript.**	

Powered by Editorial Manager® and ProduXion Manager® from Aries Systems Corporation

**Format for specific study types**<br><br>**Human Subject Research (involving human participants and/or tissue)**<br>• Give the name of the institutional review board or ethics committee that approved the study<br>• Include the approval number and/or a statement indicating approval of this research<br>• Indicate the form of consent obtained (written/oral) or the reason that consent was not obtained (e.g. the data were analyzed anonymously)<br><br>**Animal Research (involving vertebrate animals, embryos or tissues)**<br>• Provide the name of the Institutional Animal Care and Use Committee (IACUC) or other relevant ethics board that reviewed the study protocol, and indicate whether they approved this research or granted a formal waiver of ethical approval<br>• Include an approval number if one was obtained<br>• If the study involved *non-human primates*, add *additional details* about animal welfare and steps taken to ameliorate suffering<br>• If anesthesia, euthanasia, or any kind of animal sacrifice is part of the study, include briefly which substances and/or methods were applied<br><br>**Field Research**<br><br>Include the following details if this study involves the collection of plant, animal, or other materials from a natural setting:<br>• Field permit number<br>• Name of the institution or relevant body that granted permission	
**Data Availability**<br><br>Authors are required to make all data underlying the findings described fully available, without restriction, and from the time of publication. PLOS allows rare exceptions to address legal and ethical concerns. See the PLOS Data Policy and FAQ for detailed information.	Yes - all data are fully available without restriction

Powered by Editorial Manager® and ProduXion Manager® from Aries Systems Corporation

A Data Availability Statement describing where the data can be found is required at submission. Your answers to this question constitute the Data Availability Statement and **will be published in the article**, if accepted.<br><br>**Important:** Stating 'data available on request from the author' is not sufficient. If your data are only available upon request, select 'No' for the first question and explain your exceptional situation in the text box.<br><br>Do the authors confirm that all data underlying the findings described in their manuscript are fully available without restriction?	
**Describe where the data may be found in full sentences. If you are copying our sample text, replace any instances of XXX with the appropriate details.**<br><br>• If the data are **held or will be held in a public repository**, include URLs, accession numbers or DOIs. If this information will only be available after acceptance, indicate this by ticking the box below. For example: *All XXX files are available from the XXX database (accession number(s) XXX, XXX.).*<br>• If the data are all contained **within the manuscript and/or Supporting Information files**, enter the following: *All relevant data are within the manuscript and its Supporting Information files.*<br>• If neither of these applies but you are able to provide **details of access elsewhere**, with or without limitations, please do so. For example:<br><br>*Data cannot be shared publicly because of [XXX]. Data are available from the XXX Institutional Data Access / Ethics Committee (contact via XXX) for researchers who meet the criteria for access to confidential data.*<br><br>*The data underlying the results presented in the study are available from (include the name of the third party*	All relevant data are within the manuscript and its Supporting Information files

and contact information or URL).<br>• This text is appropriate if the data are owned by a third party and authors do not have permission to share the data.<br><br>* typeset	
Additional data availability information:	

Manuscript Click here to access/download;Manuscript;Rahman et al.-PlosOne-29.04.25.docx

Interactions of substrates and phosphinyl containing inhibitors with bacterial and human and zinc proteases

Fatema Amatur Rahman[1], Imin Wushur[1], Ida K. Ø. Hansen[2], Tor Haug[2], Klara Stensvåg[2], Bibek Chaulagain[1], Tra-Mi Nguyen[1], Olayiwola A. Adekoya[3], Nabin Malla[1], Jan-Olof Winberg[1], Ingebrigt Sylte[1,4*]

[1] Pharmacology and Toxicology Research Group, Department of Medical Biology, Faculty of Health Sciences, UiT The Arctic University of Norway, NO-9037 Tromsø, Norway

[2] Norwegian College of Fishery Science, Faculty of Biosciences, Fisheries and Economics, UiT The Arctic University of Norway, NO-9037 Tromsø, Norway

[3] Centre for Pharmacy, Department of Clinical Science, Faculty of Medicine, University of Bergen, Norway

[4] Centre for Research and Education, University Hospital of North Norway (UNN), Tromsø, Norway

* Corresponding author: ingebrigt.sylte@uit.no (IS)

Abstract

The bacterial virulence factors thermolysin (TLN), pseudolysin (PLN, LasB) and aureolysin (ALN) are zinc metalloproteases. In the present study, the molecular interactions and cleavage of TLN with the substrate Mca-Arg-Pro-Pro-Gly-Phe-Ser-Ala-Phe-Lys(Dnp)-OH (ES005) were studied by matrix-assisted laser desorption ionization time-of-flight mass spectrometry (MALDI-TOF MS) and molecular dynamics (MD) simulations. In addition, enzyme inhibition kinetic studies were used to test 5 compounds containing phosphinyl as the zinc binding group for their inhibition of TLN, PLN and ALN using ES005 as substrate, and of the human matrix metalloproteases MMP-9 and MMP-14 using Mca-Pro-Leu-Gly-Leu-Dpa-Ala-Arg-NH_2 (ES001) as substrate. The MALDI-TOF MS results revealed that TLN cleaves ES005 at three sites. The most abundant cleavages were between Ala and Phe, and between Gly and Phe, while the third was between Ser and Ala. MD simulations with Ala or Gly in the S_1 subpocket and Phe in S_1' subpocket gave stable interactions between ES005 and TLN. However, the MD simulations with Ser in the S_1 subpocket and Ala in the S_1' subpocket showed larger movements of ES005 relative to the catalytic site than the others, which may explain that products from the Ser-Ala cleavage were less abundant than from Ala-Phe and Gly-Phe. Compound H-1 inhibited MMP-14 and two different forms of MMP-9 with inhibition constants ranging from 0.89 to 30 μM but did not inhibit the bacterial enzymes. Induced fit docking indicated that the aromatic group of H-1, that entered the S_1' subpocket of the MMPs, is too big for the S_1' subpocket of the bacterial virulence factors. Compound H-2 inhibited the MMPs with inhibition constants ranging from 0.53 μM (MMP-9) to 3.0 μM (MMP-14) and the bacterial enzymes with inhibition constants ranging from 2.5 μM (TLN) to 80 μM (ALN). Induced fit docking indicated that H-2 bound quite differently to the human and bacterial zinc proteases.

Introduction

Metalloproteases (MPs) comprise a heterogenous family of proteases that use a metal ion to bind the substrate and polarize a water molecule to perform the hydrolytic cleavage. In the present paper we are focusing on the bacterial MPs thermolysin (TLN), aureolysin (ALN) and pseudolysin (LasB elastase, PLN) of the M4 family and the human matrix MPs (MMPs), MMP-9 and MMP-14, that belong to the M10 family of proteases [1]. All these enzymes utilize a zinc ion for hydrolytic cleavage. The zinc ion is coordinated with the polypeptide backbone through interactions with two histidines and a third amino acid, which is a histidine in the MMPs [2], and a glutamic acid in TLN, PLN and ALN [3]. Additionally, a general base is required, that accepts a proton from the polarized water molecule and transfers it to the nitrogen of the scissile bond. In both MMPs and bacterial M4 family enzymes, the general base is the glutamic acid in the conserved HExxH motif, while the histidines in this motif serve as the two zinc coordinating residues [3, 4].

Human MMPs constitute a family of zinc MPs with important functions in many physiological processes and are overexpressed in various diseases. MMPs can have a dual role, either promoting disease initiation and progression, or preventing it [2, 5-8]. They were once considered promising drug targets, and several compounds that to a certain extent selectively inhibited MMP activity were developed both by pharmaceutical companies and the academia [9-18]. However, clinical trials with MMP inhibitors were disappointing, resulting in a reduced research focus into MMPs as drug targets [19]. Based on recent progress in understanding their physiological role and their duality in different diseases, there has been a rebirth for the development of new MMP inhibitors as putative new therapeutics [19, 20].

PLN, ALN and TLN are secreted bacterial virulence factors [3]. The use of inhibitors against virulence factors is a promising new strategy in the treatment of bacterial infections,

either used alone or as an adjuvant to antibacterial drugs [21-23]. PLN is the principle extracellular virulence factor of the Gram-negative pathogen *Pseudomonas aeruginosa* [24]. This bacterium causes severe infections in the lungs, skin, eyes, soft tissues, urinary tract, and bloodstream, and is the etiologic agent of chronic airway infections in patients with chronic bronchitis, bronchiectasis, and cystic fibrosis [24, 25]. The pathogen was listed in the category "critical" on the World Health Organization's (WHO) priority list of bacterial pathogens for which research and development of new antibiotics are urgently needed [26]. ALN is secreted by the Gram-positive bacterium *Staphylococcus aureus*, one of the most harmful human pathogens, associated with methicillin resistance [27], that can cause serious infections if it enters the bloodstream or internal tissues. *S. aureus* is implicated in several different infections ranging from mild skin-related infections to more severe life-threatening and systemic infections such as bacteremia [28, 29]. TLN is a thermostable protease of industrial use secreted by the Gram-positive bacterium *Bacillus thermoproteolyticus* [3]. The enzyme is the prototype of the M4 family of proteases, also termed the thermolysin family.

Most compounds that inhibit zinc MPs contain a zinc binding group (ZBG) in addition to a scaffold that interacts with subsites of the active site cleft. Well studied ZBGs include hydroxamic acid (HONH-CO), carboxylate (CO_2^-), thiolate (S^-) and phosphinyl (PO_2^-) [30, 31]. Hydroxamic acid and thiolate are stronger ZBGs than carboxylate and phosphinyl [31, 32]. The scaffold of inhibitors with hydroxamic acid, carboxylate, or thiolate as the ZBG targets the primed or unprimed subsites. However, X-ray crystallography studies show that compounds with the phosphinyl group may act as transition state analogues, enabling the connecting scaffold to interact with both primed and unprimed subsites of the active site, potentially enhancing selectivity [14, 16, 31-34]. The contribution of different ZBGs to MMP binding was studied using a scaffold combined with various ZBGs (phosphinyl, carboxyl, or hydroxamic acid) [31]. Using phosphinyl as the ZBG, gave the compound RXP470 (compound H-1 in the

present study, Fig 1). The study showed that different ZBGs affected both affinity and selectivity, as well as the inhibitor's position at the binding site [31]. RXP470 interacted with both primed and unprimed sites, whereas replacing the phosphinyl group with carboxylic acid or hydroxamic acid as the ZBG, along with the removal of a *p*-bromophenyl group, resulted in inhibitors that interacted only with the primed regions of the active site [31]. Binding strengths of inhibitors containing phosphinyl, carboxylic acids or thiolates as ZBG are pH dependent in contrast to those with a hydroxamic acid group [31, 32].

Different substrates are available for experimental studies with zinc MPs. In the present study we used matrix-assisted laser desorption ionization time-of-flight mass spectrometry (MALDI-TOF MS) and identified that TLN cleaves the substrate Mca-Arg-Pro-Pro-Gly-Phe-Ser-Ala-Phe-Lys(Dnp)-OH (ES005) at three different sites. The cleavages sites were starting points for molecular dynamics (MD) simulations of ES005 with TLN. These MD simulations were compared with simulations of the substrate Mca-Pro-Lys-Gly-Leu-Dpa-Ala-Arg-NH_2 (ES001) with MMP-9 and MMP-14. The aim of these simulations was to study the molecular interactions between the substrates and enzymes, and to identify amino acids in the primed and unprimed binding sites of the enzymes. In order to identify structural determinants for selectivity between bacterial zinc MPs and human MMPs, we previously tested compounds with different ZBGs as inhibitors [3, 35-39], including compounds with nitrogen as the donor atom for zinc chelation [40]. In this study, we also tested RXP470 (H-1) and four other compounds (Fig 1) with phosphinyl as the ZBG as inhibitors of MMP-9, MMP-14, TLN, PLN, and ALN, using the same substrates as in the MD simulations. Additionally, phosphinyl compounds were studied through induced fit docking into the MPs.

Fig. 1. H-compounds. Molecular structures of the phosphinic compounds tested by enzyme inhibition kinetics.

Material and Methods

Materials

Dimethyl sulfoxide (DMSO), TRIS, calcium chloride ($CaCl_2 \cdot 2H_2O$), sodium hydrogen phosphate (Na_2HPO_4), and sodium acetate (CH_3COONa) were from Merck (Darmstadt, Germany). Ethylenediaminetetraacetic acid (EDTA) was from Fluka (Buchs, Switzerland). Acrylamide, Coomassie Brilliant Blue G-250 and Triton X-100 were from BDH (Poole, UK). Hepes, Brij-35, silver nitrate, alkaline phosphatase-conjugated antibodies, *p*-aminophenylmercuric acetate (APMA), gelatin and α-Cyano-4-hydroxycinnamic acid (HCCA) were from Sigma-Aldrich (St. Louis, MO, USA). Magnetic trypsin beads (Mag-Trypsin) were purchased from Takera (Gothenburg, Sweden). Gelatin-Sepharose, was from GE Healthcare (Uppsala, Sweden). DC Protein Assay and unlabelled molecular weight standards were from BioRad (Richmond, CA, USA). Sf9 and High Five™ insect cells and Magic Marker molecular weight standards were from Invitrogen (Carlsbad, CA, USA). Western Blotting Luminol reagent and HRP-conjugated donkey anti-goat secondary antibody were from Santa Cruz (Santa Cruz, CA, USA). HRP-conjugated goat anti-rabbit secondary antibody was from Southern Biotech (Birmingham, AL, USA). Fetal bovine serum was from Biochrom AG (Berlin, Germany). Galardin (Gm6001), human MT1-MMP/MMP-14 (catalytic domain), TLN and PLN were from Calbiochem (San Diego, CA, USA), while ALN was from BioCentrum Ltd (Kraków, Poland). ES001 and ES005 were from R&D Systems (Minneapolis, MN, USA). Mca-PL-OH was from Bachem AG (Basel, Switzerland). Trifluoroacetic acid (TFA) and acetonitrile (MeCN) of MS quality were from VWR (West Chester, PA, USA). The Peptide Calibration Standard II was from Bruker Daltonik GmbH (Bremen, Germany). Synthesis of these compounds have been described in previous papers [32-34, 41-44].

Expression, purification and activation of recombinant human proMMP-9

The expression of recombinant full length proMMP-9 (rpMMP-9) in Sf9 and High Five insect cells and the purification were performed as previously described [38, 45]. The amount of proMMP-9 was estimated spectrophotometrically at 280 nm using ε_{280nm} = 114.36 $M^{-1}cm^{-1}$ [46] for the full length enzyme. Activation of the recombinant proMMP-9 was performed with APMA (auto-activation) and Mag-Trypsin as previously described [38], while the amount of active MMP-9 was determined by active site titration using galardin, also described previously [38].

Enzyme inhibition studies of phosphinic compounds

Determination of reaction velocities. The substrate ES001 were used for APMA activated MMP-9 (MMP-9(A)), Mag-Trypsin activated MMP-9 (MMP-9(MT)) and MMP-14, while the substrate ES005 were used for ALN, PLN and TLN. The reaction velocities/initial rates (*v*) were determined at 37 °C, at an excitation wavelength of 320 nm and an emission wavelength of 405 nm with a slit width of 10 nm using either a Perkin Elmer LS 50 Luminescence spectrometer and the FL WinLab Software Package (Perkin Elmer) or a Clario Star micro plate reader (CLARIOstar® BMG LABTECH).

The phosphinic compounds were dissolved in 100% DMSO to a final concentration of 10 mM. In all assays, a fixed substrate concentration of 4.0 µM was used in a total volume of 100 µL 0.1 M Hepes pH 7.5, 10 mM $CaCl_2$, 0.005% Brij-35 and 1.0% DMSO. Fixed enzyme concentrations were as follows; 0.05 nM MMP-9(A), 0.05 nM MMP-9(MT), 1.0 nM MMP-14, 1.4 nM ALN, 0.5 nM PLN and 0.21 nM TLN. Time dependent inhibitory experiments, with and without 100 µM of phosphinic compounds, were performed as follows: Compounds were either pre-incubated with proteases for 15 and 30 minutes at room temperature before initiating

the enzymatic reaction by adding the substrate, or not pre-incubated, meaning the reaction was initiated by adding the protease to the mixture of substrate and compound. Control tests without the compound present were performed under the same conditions. Reactions were monitored for 30 minutes at 37 °C.

Determination of K_m values. K_m value was determined for ES001 with Mag-Trypsin activated recombinant MMP-9 (MMP-9(MT)). Substrate concentrations used were 1-10 µM in a total volume of 100 µL of 0.1 M Hepes pH 7.5 containing 10 mM $CaCl_2$, 0.005% Brij-35 and 1.0% DMSO. Substrate concentrations above 10 µM resulted in quenching as reported previously [37]. Initial rate experiments were performed as described above to determine enzyme reaction velocities using a LS 50 Luminescence Spectrometer and the FL WinLab Software Package (Perkin Elmer). The K_m values of ES001 for MMP-9(A) and MMP-14, as well as those of ES005 for TLN, PLN and ALN were previously determined under the same experimental conditions as in the present work [35]. During these experiments, reactions were monitored for only one minute, and initial reaction velocities were determined from the activity curves.

Determination of IC_{50} and K_i values. Determination of inhibitory constants (IC_{50}) of the phosphinic compounds were performed with concentrations ranging from 10^{-10} to 10^{-4} M in the assay or eight concentrations resulting in 10 - 90% inhibition. A fixed substrate concentration and the same enzyme and buffer conditions as described above were used. Enzymes, with or without inhibitors, were either pre-incubated for 30 minutes at room temperature or not pre-incubated, and initial rate assays were performed as described above. Assays were performed using a Clario Star microplate reader. The IC_{50} values were calculated with GraphPad Prism 5 using equation 1 or 2, depending on the concentration range of the phosphinic compounds.

$$\frac{v_i}{v_0} = \frac{1}{(1 + 10^{(pIC50 - pI)})} \quad (1)$$

$$\frac{v_i}{v_0} = \frac{1}{(1 + \frac{[I]}{IC_{50}})} \quad (2)$$

In equations 1 and 2, v_{i} represents the enzyme activity in the presence of the inhibitor, while v_0 denotes activity in its absence, pI = -log [Inhibitor] in M and pIC_{50} = - log IC_{50} in M. All experiments were performed at least in triplicate. Equation 3 describe the relationship between IC_{50} and K_{i} values for substrate competitive inhibitors, considering the fixed substrate concentration and the enzyme's K_{m} value for the substrate:

$$IC_{50} = K_{\mathrm{i}} (1 + [S] / K_{\mathrm{m}}) \qquad (3)$$

Quenching experiments. Quenching experiments were conducted as previously described [37], to assess the extent to which the compounds quenched the time dependent enzymatic increase in fluorescence of the processed substrate products. Briefly, fluorescence measurements (λ_{ex} = 320nm, λ_{em} = 405 nm, slit width = 10 nm) at various concentrations of the Mca-fluorescent product (0-100 nM McaPL-OH) of ES001 and ES005, were determined in absence and presence of various concentrations of the phosphinic compounds (0-100 µM). Primary and secondary plots were used to determine if these compounds quenched the McaPL-OH fluorescence.

MALDI-TOF Mass Spectrometry

MALDI-TOF MS spectra were recorded for samples containing different enzyme and substrate concentrations at various time points using an Autoflex Speed instrument (Bruker Daltonik GmbH, Leipzig, Germany) equipped with a 355-nm nitrogen laser for desorption and ionization. The ionization matrix, HCCA, was dissolved to 10 mg/mL in a standard solvent (50% MeCN, 47.5% MQ-water, and 2.5% TFA) by vigorous vortexing. Prior to the MALDI run, a main buffer (0.1 M Hepes, pH 7.5, 10 mM $CaCl_2$) was used to dilute TLN to stock concentrations of 1000, 100 and 50 nM, ES005 to 500 µM, and galardin to 10 µM. All stocks solutions were kept on ice. For the 0 min incubation, 1 µL TLN and 1 µL galardin were preincubated at 37 °C in 8 µL of the main buffer for 10 min before adding 1 µL of ES005. For all other time points, 1 µL of ES005 was added to tubes containing 8 µL buffer and 1 µL of TLN at different concentrations, followed by incubation at 37 °C. At each timepoint (0, 1, 3, 5, 10, 20, 30, 60, 120 min), 1 µL of galardin was added to stop enzyme activity. After the final incubation, 1 µL aliquots from each timepoint were mixed with an equal volume of HCCA and spotted onto a MALDI plate. Additional spots on the plate were assigned for the instrument Peptide Calibration Standard II (mass range from 700-3200 Da) and blank controls containing only buffer and HCCA. Samples were dried at room temperature before loading into the MALDI-TOF MS. Spectra were acquired at a laser power of 100 mV in reflector mode with a mass to charge (m/z) range of 500-1700. The resulting mass spectra were analysed using flexAnalysis software (v3.3, Bruker).

Molecular modelling

Molecular modelling was performed using the Schrödinger 2021-2 software suit (Schrödinger, Inc., New York, USA, 2021). The Desmond program [47]

(https://www.deshawresearch.com/publications/Desmond-GPU_Performance_April_2021.pdf) version 6.7 running on the graphics processing unit (GPU) of our workstations was used for MD simulations of ES001 with MMP-9 and MMP-14, and of ES005 with TLN.

Induced fit docking of H-1 and H-2. Induced fit docking of H-1 and H-2 (Fig 1) was performed within the active site cleft of MMP-9, MMP-14, TLN, ALN and PLN. The 2D structures of H-1 and H-2 were drawn and converted to 3D using the 3D builder tools of Maestro 12.8. Different enantiomeric states were generated and optimized with the LigPrep module using the OPLS4 force field [48]. The obtained low energy conformations were used for induced fit docking (IFD). The following X-ray crystal structures were selected for docking: MMP-9 (PDB ID: **2OVZ**), a Glu402Gln mutant complexed with a phosphinate inhibitor; MMP-14 (PDB ID: **1BQQ**), in complex with TIMP-2 (an X-ray structure of MMP-14 with a small molecular inhibitor is not available in the PDB-database); PLN (PDB ID: **3DBK**), in complex with the inhibitor phosphoramidone; TLN (PDB ID: **1TLP**), in complex with a phosphoramidate inhibitor; and ALN (PDB ID: **1BQB**), representing the enzyme without inhibitor (an ALN-inhibitor complex was not available in the PDB-database).

Gln402 in the MMP-9 structure was mutated back to Glu402, as in the wild type MMP-9. Enzyme structures were protonated at pH 7.4 $\pm$ 0.2 using the Epik tool, missing hydrogen atoms were added, and structures were optimized with the OPLS4 force field [48]. An atomic distance of 12 Å around the centre of the co-crystallized inhibitors was used to generate the 3D grid box for docking. For PDB structures lacking a small molecular inhibitor (MMP-14 and ALN), the catalytic zinc atom served as the centre of the 3D grid box. IFD was performed for the H-1 and H-2 using default parameters and standard sampling, retaining 20 poses of each ligand. The IFD scores were calculated to rank the different poses.

Construction of enzyme-substrate complexes for MD. The X-ray structure of an inactive MMP-9 mutant (Glu227Ala, corresponding to Glu402Ala in this manuscript) complexed with a substrate probe molecule (PDB ID: **4JIJ**), along with an X-ray structure of MMP-9 containing the prodomain, catalytic domain, and three FnII (fibronectin type II) modules (PDB ID: **1L6J**), were used as starting points to generate MMP-9/ES001, MMP-14/ES001, and TLN/ES005 complexes. The prodomain was deleted from the 1L6J structure, resulting in Phe107 as the new N-terminal residue, as observed in trypsin and MMP-3 activated MMP-9 [49].

The substrate ES001 has the same length as that of the substrate probe (Fig 2), with the only structural difference being the substitution of a *para* iodinated Phe in the probe [50] with a Leu in ES001. The iodinated Phe occupies the S_1' binding cavity of MMP-9 [50], indicating that the corresponding Leu in ES001 most likely resides the P_1' position (Fig 1). Crystallographic water molecules were removed from both structures, and an initial MMP-9 - ES001 complex was constructed from the MMP-9-substrate probe complex (PDB ID: **4JIJ**) by mutating the iodinated Phe in P_1' position to Leu. This MMP-9-ES001 complex was then superimposed onto a modified 1L6J structure (without the prodomain), allowing ES001 to be transferred into the catalytic site of the modified 1L6J structure. The 1L6J structure was chosen for MD simulations due to the influence of its three FnII domains on structural dynamics.

An initial MMP-14-ES001 complex was constructed by superimposing the X-ray structure of MMP-14 (PDB ID: **1BQQ**), with crystallographic water molecules removed, onto the generated MMP-9-ES001 complex (modified 1L6J). The ES001 structure was then transferred into the active site cleft of MMP-14 in a position similarly to the position in MMP-9.

Fig. 2. **Substrate structures**. Two dimensional (2D) illustrations of the substrate probe from the X-ray crystallographic complex with MMP-9 (PDB ID: **4JIJ**), as well as the substrates Mca-Pro-Leu-Gly-Leu-Dpa-Ala-Arg-NH_2 (ES001) and Mca-Arg-Pro-Pro-Gly-Phe-Ser-Ala-Phe-Lys(Dnp)-OH (ES005). ES001 was used in enzyme inhibition studies with MMP-9 and MMP-14, while ES005 was used in studies with TLN, PLN, and ALN. The P1 and P1' residues in MD simulations of ES001 with MMP-9 and MMP-14, and of ES005 with TLN, are indicated in the figure.

Constructing TLN complexes with ES005 was more challenging due to the greater length of ES005 compared to ES001 and the substrate probe (Fig 2). This made direct generation of the ES005 structure from the substrate probe problematic. The X-ray structure of MMP-9 with the substrate probe (PDB ID: **4JIJ**) was superimposed with the X-ray structure of TLN (PDB ID: **1TLP**), without crystallographic water molecules, and the substrate probe was transferred into the TLN structure. ES005 was then drawn, converted to 3D using the 3D Maestro 12.8 3D builder tools, and optimized with the LigPrep module using the OPLS4 force field [48]. MALDI-TOF MS studies revealed that TLN cleaves ES005 at three sites: between Gly and Phe, between Ala and Phe, and between Ser and Ala (Fig 2). Three complexes of ES005 with TLN were therefore constructed, by manually positioning ES005 on top of the substrate probe within the TLN structure. For each complex, the P_1'residue of the cleavage site (Phe, Phe and Ala, respectively) was positioned in the S_1' subpocket of TLN. In the two complexes were Phe occupied the S_1' subsite, it was superimposed onto the 4-iodo-phenylalanine of the substrate probe. In the third complex, were Ala occupied the S_1' subpocket, its backbone atoms and Cβ-atom of Ala were superimposed with the corresponding atoms of 4-iodo-Phe of the substrate probe. Manual adjustments of ES005 side chains were necessary to prevent structural clashes in all three complexes.

The enzyme structure complexes of ES001 with MMP-9 and MMP-14, as well as the three TLN-ES005 complexes, were protonated at pH 7 $\pm$ 2 using the Epik tool. Missing hydrogen atoms were added, and complexes were optimized using the OPLS4 force field [48].

Molecular dynamics simulations. The five energy optimized substrate-enzyme complexes were used as starting points for 200 ns of MD simulations. The OPLS4 force field

[48] was also used for MD simulations. Each molecular system was solvated in an orthorhombic box by using the TIP4P water model with a region of 10 Å between the surface of the enzyme-substrate complexes and the box walls. Chloride ions were added to neutralise the system. Equilibration of the molecular systems was performed using the default relaxation protocol, which includes 100 ps of Brownian dynamics at constant temperature (10 K) and pressure, with restraints applied to solute heavy atoms. This was followed by a gradual heating process from 0 to 300 K. A cutoff radius of 9 Å was applied for short range electrostatic and van der Waals interactions, while long range electrostatic interactions were treated using the smooth particle mesh Ewald method [51]. After convergence, MD simulations for 200 ns were performed under isothermal-isobaric ensemble (NPT) (temperature: 300 K, pressure: 1.01325 bar) with time steps of 2 fs. A Nose-Hoover thermostat and the Martyna-Tobias-Klein barostat were used to maintain constant temperature and pressure [52]. Coordinates were written every 100 ps, giving all together 2000 frames in each trajectory.

Results and discussion

Enzyme activity of the five MPs against ES001 and ES005

The enzymatic activity of TLN, ALN, PLN, MMP-9(MT), MMP-9(A) and MMP-14 in the cleavage of both ES001 and ES005 were studied. In several of our previous studies, ES001has been used as a substrate for human MMPs, while ES005 had served as a substrate for bacterial virulence factors [35, 38, 40]. However, we have not demonstrated that bacterial enzymes prefer ES005 over ES001 and that human MMPs favour ES001 over ES005. To compare the activity of these enzymes against ES001 and ES005 assays were conducted using the same assay condition for all enzymes. The differences from the testing of phosphinic compounds described in the Material and Methods section were that DMSO was omitted, and

the concentration of both substrates was 10 µM. A Perkin Elmer LS50 Luminescence Spectrometer was used to measure the activity. Reactions were monitor for one minute, and initial reaction velocities were determined from the activity curves. For each enzyme, the reaction with the presumed optimal substrate was performed first at an arbitrary enzyme concentration, followed immediately by the reaction with the alternate substrate using the same enzyme concentration.

Table 1. Enzymatic activity (ΔF/min*).* Enzymatic activity of bacterial (TLN, PLN and ALN) and human (MMP-9(A), MMP-9(MT), MMP-9(A) and MMP-14) enzymes against ES001 and ES005 (n=4 in all assays).

Enzyme	Substrate	
	ES001	ES005
TLN	27 ± 3	326 ± 36
PLN	9 ± 1	138 ± 4
ALN	65 ± 8	354 ± 61
MMP-9(A)	77 ± 5	5.1 ± 0.2
MMP-9(MT)	363 ± 22	18 ± 8
MMP-14	230 ± 19	8 ± 2

The results demonstrated that the three bacterial enzymes had higher activity against ES005 than against ES001 (Table 1). TLN showed approximately 13-fold higher activity for ES005, while PLN and ALN displayed approximately 15-fold and 5-fold higher activity, respectively. However, the human enzymes showed a preference for ES001 over ES005. MMP-9(A) had around 15-fold, MMP-9(MT) approximately 20-fold, and MMP-14 nearly 29-fold higher activity against ES001 than ES005. Based on these findings, we selected ES001 as the substrate for enzyme inhibition studies with MMP-9 and MMP-14, while ES005 was used for inhibition studies with TLN, PLN, and ALN.

MALDI-TOF MS analysis of ES005 cleavage sites

TLN is known to prefer hydrophobic amino acids within the S_1' subpocket [3], but the exact cleavage sites of ES005 (Fig 2) remain unidentified. To determine these sites, we performed a MALDI-TOF MS analysis. No TLN cleavage products were detected in the MALDI-TOF MS spectra immediately after incubating 50 µM ES005 (0 min incubation) with 10 nM TLN (Fig 3). The calculated m/z $[M+H]^+$ value of ES005 is 1388.59 and the peak was observed at 1388.62, verifying the molecular weight of ES005 to be 1387.61 Da. At time zero, several peaks with slightly lower molecular masses were also observed, likely corresponding to the loss of O, O_2, NO and NO_2 due to fragmentation of the 2,4-dinitrophenyl group (Dnp) of the substrate (S1 Table). Furthermore, a peak observed of around m/z 1221.4 $[M+H]^+$ is also likely a MALDI-induced fragment of ES005, caused by complete loss of Dnp from the substrate (Fig 3).

Fig. 3. **MALDI-TOF MS spectra.** MS spectra obtained after 0 and 5 minutes incubation of 50 µM (Mca-Arg-Pro-Pro-Gly-Phe-Ser-Ala-Phe-Pro-Lys(Dnp)-OH) ES005 with 10 nM thermolysin (TLN). The m/z values marked in green, blue and red represents N-terminal cleavage products of ES005 obtained after AF, SA, and GF cleavage, respectively.

Table 2. MALDI-TOF MS data obtained after TLN cleavage of ES005. The m/z values (representing $[M+H]^+$ ions) of ES005 and the three observed cleavage products are shown. The observed m/z values represent the average value from all measurements performed.

**Substrate/fragment sequence**	**Formula**	**Calculated m/z $[M+H]^+$**	**Observed m/z $[M+H]^+$**
Mca-Arg-Pro-Pro-Gly-Phe-Ser-Ala-Phe-Lys(Dnp)-OH (ES005)	C66H81N15O19	1388.59	1388.62[1]
Mca-Arg-Pro-Pro-Gly-Phe-Ser-Ala-OH (AF cleavage)	C45H58N10O13	947.43	947.42[2]
Mca-Arg-Pro-Pro-Gly-Phe-Ser-OH (SA cleavage)	C42H53N9O12	876.39	876.35
Mca-Arg-Pro-Pro-Gly-OH (GF cleavage)	C30H39O9N7	642.29	642.20[3]

[1] The $[M+Na]^+$ ion (m/z 1410.58) was also observed. [2] The $[M+Na]^+$ ion (m/z 969.39) was also observed. [3] The $[M+Na]^+$ ion (m/z 664.18) was also observed.

After 5 minutes of incubation with 10 nM TLN, three distinct cleavage products (m/z peaks representing $[M+H]^+$ ions) of ES005 were observed (Fig 3, Table 2). Based on the observation of N-terminal sequences, ES005 was cleaved at three sites: Gly-Phe (m/z 642), Ser-Ala (m/z 876), and Ala-Phe (m/z 947). The most dominant cleavage site was Ala-Phe,

followed closely by Gly-Phe, while Ser-Ala cleavage was less abundant (Fig 3). This cleavage pattern was observed at 1 minute with 5 nM TLN (S1 and S2 Figs) and remained up to 30 minutes with 5 nM TLN and 5 minutes with 10 nM TLN (Fig 3, S1 and S2 Figs). Longer incubation at both TLN concentrations gave increased cleavage at the Gly-Phe site relative to the Ala-Phe site. This effect was more pronounced when the full-length ES005 substrate was almost or completely digested (S1 and S2 Figs). These findings suggests that the Ala-Phe cleavage product (Mca-Arg-Pro-Pro-Gly-Phe-Ser-Ala) was further cleaved to yield the Gly-Phe cleavage product (Mca-Arg-Pro-Pro-Gly). One μM galardin was not sufficient to completely inhibit 100 nM of TLN. As a result, substrate cleavage was observed immediately (0 minutes) with a pattern identical to that seen at lower TLN concentrations (S4 Fig). After 30 minutes of incubation, the peak corresponding to full-length ES005 had disappeared. By 60 minutes, both the Ala-Phe cleavage fragment (Mca-Arg-Pro-Pro-Gly-Phe-Ser-Ala) and the Ser-Ala cleavage fragment (Mca-Arg-Pro-Pro-Gly-Phe-Ser) were no longer detectable. The only observed fragment was the Gly-Phe cleavage product (Mca-Arg-Pro-Pro-Gly) (S4 Fig). With increased incubation time, the peak corresponding to Mca-Arg-Pro-Pro-Gly peak (Gly-Phe cleavage) became more prominent compared to the two other product peaks. This suggests that the cleavage products Mca-Arg-Pro-Pro-Gly-Phe-Ser-Ala and Mca-Arg-Pro-Pro-Gly-Phe-Ser were further processed into Mca-Arg-Pro-Pro-Gly.

Molecular dynamics simulations of substrate-enzyme interactions

The binding modes of ES001 with MMP-9 and MMP-14, as well as ES005 with the bacterial virulence factors, are not known from structural studies. However, the binding mode of a substrate probe molecule to a mutated mini-variant of MMP-9 has been investigated using X-ray crystallography [50]. We utilized this X-ray complex as a starting point to obtain more information about the binding of ES001 to MMP-9 and MMP-14, as well as ES005 to TLN,

using MD simulations. The aims were to support our MALDI-TOF MS results, examine enzyme-substrate interactions in both bacterial and human zinc proteases, and identify key amino acids constituting the most important subpockets of these enzymes.

Interactions of ES001 with MMP-9 and MMP-14. The only structural difference between ES001 and the substrate probe molecule co-crystallized with MMP-9 [50] is the P_1' residue, which is a 4-iodo-Phe in the substrate probe and Leu in ES001 (Fig 2). The X-ray crystal structure indicated that the substrate probe binds MMP-9 in an elongated conformation, with the N-terminal Mca group in the unprimed direction and the C-terminal arginine in primed direction. ES001 was docked into MMP-9 in a similar starting orientation, where the Gly occupied the S_1 subpocket and the Leu occupied the S_1' subpocket (Fig 2). During 200 ns simulations both with MMP-9 and MMP-14, the substrate maintained its orientation and elongated conformation within the binding site cleft throughout the simulations. In both enzymes, the terminal parts of ES001 showed the greatest structural flexibility, with the N-terminal Mca and the Dpa in the C-terminal being most exposed to the solvent (Fig 4). The root means square deviations (RMSDs) of the complexes from the starting complexes during MD simulations indicate structurally stable complexes, and the substrate formed stable interactions with subpockets of the enzymes (S5 Fig). After approximately 25 ns, the RMSD for the substrate relative to the starting conformation stabilized at approximately 3 Å for MMP-9 and 4 Å for MMP-14 (S5 Fig, Lig fit Lig).

Fig. 4. Molecular interactions of ES001 with MMP-9 and MMP-14. Left: Close up view of the binding cleft of MMP-9 and MMP-14 with ES001 (green) at the catalytic site. Oxygen atoms of ES001 in red, nitrogen atoms in dark blue, while hydrogen atoms connected to nitrogen or oxygen atoms are in white. The surface of the binding cleft depicted in yellow, with the P_1' residue indicated. The Cα trace of the catalytic part is shown in light blue. MD frames after 161 ns of simulation with MMP-9 and after 125 ns with MMP-14 are used as representatives for the MD frames. The yellow backbone Cα trace of MMP-9 highlights the fibronectin repeats. Right: 2D illustration of the complexes with the most important ES001-enzyme interactions, where amino acids within 4 Å of the substrate are included. The percentages represent the frequency of these interactions observed in MD frames, with only those present in more than 30% of the frames included.

During 200 ns MD simulation with MMP-9, the backbone NH group of the Leu residue in the P_2 position of ES001 interacted with Ala191 (85% of frames), while the carbonyl oxygen

of Gly at the P_1 position interacted with the catalytic zinc ion (100% of frames). The NH group of Leu at the P_1' position interacted with the catalytic glutamic acid (Glu402) in 30% of frames. One of the negatively charged oxygen atoms and a nitrogen atom of Dpa in P_2' position interacted with the catalytic zinc for most of the simulation (Fig 4). Additionally, the backbone NH group and a side chain nitrogen of Dpa formed interactions with the backbone carbonyl group of Pro421. A water molecule was bridging between Leu188 and the backbone carbonyl oxygen in P_1'-position, while Tyr423 interacted with the backbone carbonyl oxygen in P_2'-position in more than 30% of the frames (Fig 4, S6 Fi[illegible] Overall, ES001 obtained a hydrogen bonding network with MMP-9 very similar to that of the substrate probe in the X-ray complex of MMP-9 [50[illegible]

During 200 ns MD with MMP-14, the interactions of the central regions of ES001 around the cleavages site were quite like those with MMP-9. ES001 maintained comparable interactions with the catalytic zinc ion and the catalytic glutamic acid (Glu240) as with MMP-9 (Fig 4, S6 F[illegible] but the interaction fraction with Glu240 during the MD was higher for MMP-14 (Fig 4, S6 Fig). However, the interactions around the P_2 and P_2' positions differed significantly from that in MMP-9 for most of the simulation frames (Fig 4).

Interactions of ES005 with TLN. MALDI-TOF MS studies showed that ES005 is cleaved at three sites (Table 2), and three MD simulations were therefore performed: 1. Gly as the P_1 residue and Phe as the P_1' residue (GF-MD). 2. Ala as the P_1 residue and Phe as the P_1' residue (AF-MD). 3. Ser as the P_1 residue and Ala as the P_1' residue (SA-MD). ES005 is larger than both ES001 and the substrate probe co-crystallized with MMP-9 [50]. As a result, docking ES005 into the catalytic site of TLN was more challenging than for ES001, making the starting complexes for MDs less certain compared to those of ES001.

During the GF-MD, ES005 retained an elongated conformation throughout the entire simulation, with the N-terminal Mca extending in the unprimed direction and the C-terminal

Dnp in the primed direction (Fig 5). This orientation was like that observed for ES001 in MMP-9 and MMP-14 (Fig 4) and in the X-ray structure of the substrate probe with MMP-9 [50]. The RMSD of ES005 relative to its starting conformation remained stable around 6 Å from the beginning of the 200 ns sampling period (S7 Fig, (Lig) fit on Lig), suggesting both internal structural stability and consistent interactions with TLN throughout the simulation. The catalytic zinc interacted with the backbone carbonyl group of both Gly in the P_1 position and Pro in the P_2 position. Asn112 interacted with the backbone NH group of both Phe in P_1’ and Ser in P_2’, while Arg203 interacted with the backbone CO in P_1’ position. Asn111 interacted with side chain of Ser in P_2’, while His142 was stacking with the side chain Phe in P_1’ position (Fig 5).

During the AF- MD, the RMSD of ES005 relative to its starting conformation stabilized around 7 Å (S7 Fig, (Lig) fit on Lig). After approximately 100 ns, ES005 obtained a very stable conformation and stayed in that conformation for the rest of the simulation. In the first 100 ns, the N-terminal region of ES005 did not have binding partners within the enzyme, and large portions of the substrate (especially in the N-terminal parts) were highly exposed to solvent. However, after 100 ns, the N-terminal Mca group folded back towards the C-terminal Dnp group, resulting in a folded and very stable conformation of the substrate. Notably, during the AF-MD, the unprimed amino acids of ES005 were more important for forming interactions with amino acids in TLN than in the GF-MD complex (Fig 5). The backbone carbonyl group of Gly (P_4 position) obtained hydrogen bonds with Asn116, while the backbone carbonyl of Pro in P_6 position formed an internal hydrogen bond with the amide group of Phe in P_3 position. The backbone NH of Ser in (P_2) interacted with Trp115. The side chain of Phe in (P_3) was stacking with Phe114. The backbone carbonyls of Ser (P_2) and Ala (P_1) interacted with the catalytic zinc ion. The backbone NH of Phe (P_1’) interacted with Asn112, while its side chain stacked with His142. Arg203 interacted with at the C-terminal end of ES005 (Fig 5).

Fig. 5. Molecular interactions of ES005 with TLN. Left: Close ups of the TLN binding cleft surface (yellow) during the three MD simulations: TLN/ES005-GF, TLN/ES005-AF and TLN/ES005-SA. The Cα-trace of TLN is shown in light blue. ES005 atoms are colour coded: carbon in green, oxygen in red, and nitrogen in dark blue. The representative coordinate sets were selected at 137 ns, 160 ns, and 200 ns for the TLN/ES005-GF, TLN/ES005-AF, and TLN/ES005-SA simulations, respectively. Right: 2D illustration of the TLN/ES005 complexes, highlighting the most significant interactions. Amino acids within 4 Å of the substrate are included, with interaction frequencies indicated as percentage of MD frames. Only interaction present in more than 30% of the frames are included.

The RMSD plots during the SA-MD indicate that the conformation of ES005 was more unstable relative to the starting conformation than in the AF-MD and GF-MD. The RMSD (S7 Fig, (Lig) fit on Lig) varies between approximately 3.4 Å and 6.8 Å, suggesting considerable internal structural fluctuations. Frame analysis revealed that the largest fluctuations occurred in the N-terminal unprimed region of the substrate. During the SA-MD, the carbonyl group of the amino acid in P_1 position interacted with the catalytic zinc (Fig 5), similarly to the AF- and GF-MD simulations. Additionally, the carbonyl group of the Ala in the P_1’ position and a negatively charged oxygen atom at the C-terminus also interacted with the catalytic zinc (Fig 5). Arg203 further stabilized the C-terminal region by interacting with the carbonyl group of Phe in the P_2’ position and a terminal oxygen of ES005 (Fig 5).

During catalytic cleavage, the incoming substrate is presumed to displace a water molecule coordinated to the catalytic zinc. This displacement allows the general base (Glu143 of the HEXXH motif) to accept a proton from the zinc polarized water molecule and transfer it to the leaving nitrogen of the scissile bond [53, 54]. Throughout the cleavage process, the side chain oxygen of Asn112 in TLN and the backbone carbonyl of Ala113 are suggested to form hydrogen bonds with the protonated nitrogen in the P_1’ position of the scissile bond. In both the AF-MD and GF-MD, Asn112 formed hydrogen bonds with the backbone NH group of the P_1’ residue, while the general base Glu143 remained close to the NH group of the P_1’ residue. As indicated by the residual interaction fraction, Asn112 played a crucial role in interacting with ES005 in both simulations, with interaction fractions of approximately 1.5 during AF-MD and 2.4 during GF-MD (S8 Fig). In contrast, during the SA-MD, Asn112 did not interact with the NH group of the P_1’ residue (Fig 5) and had only limited involvement in ES005 interactions,

with an interaction fraction of approximately 0.2 (S8 Fig). The residual interaction fractions show that 13 hydrogen bonds were present between TLN and ES005 during the AF-MD, compared to 10 in the GF-MD and 12 in the SA-MD. However, the residual interaction fractions were much higher for the AF- and GF-MDs than for the SA-MD (S8 Fig). Similarly, the fractions for ionic and hydrophobic interactions were generally higher in the AF- and GF-MDs, while the number of water bridges was relatively consistent across all three complexes.

The three MD simulations showed that ES005 fits better into the catalytic site and obtain more favourable interactions with TLN when the Ala or Gly occupy the S_1 subpocket and Phe the S_1' subpocket, compared to when Ser occupies the S_1 and Ala the S_1' subpocket. This aligns with the MALDI-TOF MS results. The SA-MD suggests that the side chain of Ala is too short to obtain a maximum of favourable contacts with amino acids within the S_1' subpocket. This finding is consistent with previous studies indicating that TLN preferentially cleaves at the N-terminal side of hydrophobic or bulky amino side chains, such as Leu, Phe, Ile, and Val. However, TLN may also cleave at the N-terminal side of residues like Met, His, Tyr, Ala, Asn, Ser, Thr, Gly, Lys, Glu, or Asp [3, 55].

Subpocket amino acids. The amino acids within the substrate subpockets during MD simulations of ES001 with MMP-9 and MMP-14 and ES005 with TLN (GF-MD) are listed in Table 3. Amino acids in corresponding subpockets of PLN and ALN were identified through sequence alignments of the catalytic domains of TLN, PLN, and ALN (S9 Fig). For an amino acid located at the boarder of two subpockets, their assignment may depend on the substrate structure and the dynamic behaviour of the substrate-enzyme complex. As a result, Table 3 is therefore slightly different from a corresponding table based on docking of inhibitors by Rahman et al. 2021 [35].

Table 3. Amino acids within different subpockets of MMP-9, MMP-14, TLN, PLN and ALN. Amino acids included are those located near substrate side chains during MD simulations with MMP-9, MMP-14, and TLN (GF-MD). Subpocket amino acids of PLN and TLN were identified based on amino acid sequence alignments with TLN (S9 Fig).

Subpocket	MMP-9	MMP-14	TLN	PLN	ALN
**S_1**	H190, A191, E402, H405	F198, A200, A202, E240, H243	A113, F114, Y157	A113, Y114, Y155	A116, A117, Y160
**S_1'**	Y393, S394, F396, L397, V398, H401, L418, Y420, P421, M422, Y423, R424, T426	L199, L235, N231, V236, V238, H239, F260, Y261, Q262, F263	N112, M120, S134, D138, V139, H142, G189, L202, R203	N112, M120, L132, D136, V137, H140, G187, L197, R198	N115, M123, S137, D141, V142, H145, G187, L200, R201
**S_2**	A191, P193	H201, Y203, F204	W115, E143, H146	W115, E141, H144	W118, E146, H149
**S_2'**	G186, L187, Y218, H411	H249, S250, S251, P259	N111, F130, L133, Y193	E111, Y130, L132, K191	N114, F133, L136, Y191

To be potential drug candidates for treating of *P. aeruginosa* or *S. aureus* infections, PLN and ALN inhibitors should not inhibit human zinc proteases. The amino acids forming the substrate subpockets are quite similar between the bacterial and human zinc proteases studied (Table 3). The major subpocket for substrate cleavage specificity is the S_1' subpocket, which is mainly hydrophobic in both bacterial and human enzymes. However, key differences in this subpocket may be important for designing inhibitors that selectively target the virulence factors over MMP-9 and MMP-14. The S_1' subpocket is both deeper and wider in the human enzymes, suggesting they can accommodate larger side chains compared to the bacterial enzymes. Notably, a positively charged Arg is present in the S_1' subpocket of all three bacterial enzymes (S9 Fig), while this residue is absent in MMP-9 and MMP-14 (Table 3). Although MMP-9 contains an Arg in the S_1' subpocket, it is located much deeper in the pocket than in the bacterial enzymes. Targeting the Arg in the bacterial enzymes could be a promising strategy for developing inhibitors with stronger binding affinity for bacterial virulence factors than for MMP-9 and MMP-14.

Enzyme inhibition and docking of the phosphinic compounds

K_m values of fluorescence quenched substrates. Under the experimental conditions used in the present work (1% DMSO in all assays), the K_m value of ES001 with recombinant MMP-9(MT) was 2.7 ± 0.5 µM. The K_m values for ES001 with MMP-9(A) and MMP-14, as well as for ES005 with TLN, PLN, and ALN, were previously determined under the same conditions [35]. To summarize, the K_m values for ES001 with MMP-9(A) and MMP-14 were 4 ± 1 µM and 4.9 ± 0.4 µM, respectively. For ES005, the K_m values with ALN, PLN, and TLN were 76 ± 7 µM, 24 ± 8 µM, and 6 ± 1 µM, respectively. Although the estimated K_m values for ALN and PLN were regarded less certain due to substrate quenching at concentration above 10 µM, the values were consistent with previous measurements conducted either without DMSO [38] or with 5% DMSO [37].

Quenching experiments. All compounds were tested for possible quenching of the fluorescence product. Experiments were performed with varying concentrations of the putative inhibitors (0-100 µM) against varying concentrations of the fluorescence product (McaPLG-OH) of ES001, as previously described for procaspase-activating compound 1(PAC-1) and isatin derivatives [37], and outlined in Materials and Methods. These experiments revealed that none of the phosphinic compounds quenched the fluorescence product. However, some compounds showed background fluorescence at the emission and excitation wavelengths used, though this did not interfere with the inhibitory assays, as the enzymatic reactions were followed continuously. These findings agree with our previous results for bisphosponate and catechol-containing compounds [35], as well as for dipicolylamine (DPA), tripicolylamine (TPA), tris pyridine ethylene diamine (TPED), and various pyridine and thiophene deriviatives [40]. In contrast, PAC-1 and isatin derivatives were found to significantly quench the fluorescence product [37].

Fig. 6. Inhibitory effects. The inhibitory effects of 100 µM of the H-compounds on the enzymatic activity of MMP-14, MMP-9(MT), MMP-9(A), TLN, PLN and ALN.

Inhibitory effects and binding modes of the phosphinic compounds. Except for H-3 against MMP-14, the compounds were not slow binders to any of the proteases (S10 Fig). For H-3, inhibition of MMP-14 appeared to stabilize after a 15 min pre-incubation. Among the tested compounds, only H-2 showed strong inhibition across all five proteases, with the most potent effects observed against MMP-9 and MMP-14 (Fig 6). In contrast, H-1 exhibited strong inhibition exclusively against the two human proteases, while H-3, H-4, and H-5 showed weak or no inhibition of the enzymes. K_i values were determined for the enzymes where 100 µM of a phosphinic compound resulted in ≥ 50% inhibition (Table 4). H-2 exhibited the strongest binding to the MMP-9 variants (MMP-9(MT) and MMP-9(A)), with K_i values around 0.5 µM. The compound bound with approximately equal affinity to MMP-14 and TLN, although with lower affinity than for MMP-9. A much weaker binding was observed for PLN and ALN, although the binding was stronger to the former enzyme.

Table 4. K_i values of the phosphinic compounds H-1 and H-2 for MMP-14, MMP-9(A), MMP-9(MT), ALN, PLN and TLN. The K_i ± s.d. values were determined for compounds that reduced enzymatic activity by 50% or more at a concentration 100 µM. The s.d. of the K_i values accounts for the combined s.d. from both the IC_{50} and K_m values. The contribution of the K_m s.d. is weighted relative to the [S]/K_m ratio in the calculation of K_i from IC_{50} (Eqn. 3).

Compound	K_i ± s.d. (µM)					
	ES001			ES005		
	MMP-14	MMP-9(A)	MMP-9(MT)	ALN	PLN	TLN
H-1	0.89 ± 0.04	19 ± 3	30 ± 4	*n.d.*[a]	*n.d.*	*n.d.*
H-2	3.0 ± 0.1	0.56 ± 0.07	0.53 ± 0.06	80 ± 4	40 ± 2	2.5 ± 0.2

[a]*n.d.*, not determined

Fig. 7. Induced fit docking of H-2 with MMP-9. A: The docked complex of H-2 (gold-yellow, RSS enantiomer) with MMP-9 (light blue Cα-trace) superimposed with the X-ray structure of the MMP-11 complex (PDB ID: **1HV5**) (yellow Cα-trace) with H-2 (green, SSS enantiomer). H-2 oxygen atoms are shown in red, nitrogen atoms in dark blue, the catalytic zinc in grey and the phosphate group in dark red. The RSS enantiomer was used in the enzyme kinetics studies. B: The same H-2-MMP9 complex as in A, with identical colour coding. The most important amino acids involved in binding are included.

H-2 was docked into the active site of all five MPs using induced fit docking. The highest scoring pose of H-2 with MMP-9 (gold-yellow in Fig 7) had a docking score of -16.3 kcal/mol. All enantiomers were initially docked, but the RSS enantiomer (Fig 7) had the highest

score and was also used in the enzyme inhibition kinetics studies. The X-ray structure of the SSS enantiomer bound to MMP-11 is available (PDB ID: **1HV5**) [42], and this complex was superimposed with the MMP-9/H-2 induced fit docking complex in Fig 7A. The structures showed large similarities: in both, H-2 interacts with both primed and unprimed subpockets, and the propylphenyl group occupies the S_1' subpocket and penetrates deeply into the pocket. The most striking difference was that the indole ring occupied the S_2' subpocket in the X-ray complex (SSS enantiomer), while in the best docked complex (RSS enantiomer), the ring extended more towards the S_3' subpocket (Fig 7B). Additionally, the positions of the other phenyl rings also differed between the two complexes.

Fig. 8. Induced fit docking of H-2 with MMP-14. The docked complex of H-2 (RSS enantiomer) with MMP-14. Colour coding as in Fig 7, and in addition, two calcium ions are shown in pink.

The position of H-2 (RSS) in MMP-14 (Fig 8) was like its position in MMP-9 (Fig7). However, in the highest scoring pose, the indole ring oriented towards the S_2' subpocket (Fig 8), resembling the X-ray complex of the SSS-enantiomer with MMP-11 (Fig 7). The docking score for H-2 in MMP-14 was - 14.9 kcal/mol.

In agreement with the obtained K_i values (Table 4), the induced fit docking studies indicated that H-2 binds weaker to TLN, PLN, and ALN than to MMP-9 and MMP-14. The docking scores for H-2 were as follows: PLN: -13.0 kcal/mol, ALN: -13.0 kcal/mol, TLN: -13.4 kcal/mol. The main differences between the bacterial MPs and human MMPs was the binding orientation of H-2. In the bacterial MPs (Figs 9-11), the phenyl ring closest to the phosphinyl group entered the S_1' subpocket, while in MMP-9 (Fig 7) and MMP-14 (Fig 8), the propylphenyl group occupied this pocket. In the bacterial enzymes, the propylphenyl group extended towards the unprimed sites, suggesting that the S_1' subpocket of the virulence factors is too small to accommodate the longer propyl chain. As a result, the shorter phenyl group entered the S_1' subpocket instead. This aligns with previous studies on hydroxamic acid-based compounds as ZBG, which showed that the S_1' subpockets of MMPs can occupy larger groups

compared to TLN, PLN, and ALN [36, 38, 39]. Notably, H-2 interacted with both prime and unprimed subsites in the bacterial MPs, consistent with its docking behaviour in MMP-9 and MMP-14. These findings indicate that H-2 and other phosphinyl compounds may mimic substrate transition states while interacting with bacterial zinc MPs and human MMPs.

Fig. 9. Induced fit docking of H-2 with PLN. The docked complex of H-2 (RSS enantiomer) with PLN. Colour coding as in Fig 7.

Fig. 10. Induced fit docking of H-2 with ALN. The docked complex of H-2 (RSS enantiomer) with ALN. Colour coding as in Fig 7.

Fig. 11. Induced fit docking of H-2 with TLN. The docked complex of H-2 (RSS enantiomer) with TLN. Colour coding as in Fig 7.

H-1 binds stronger to MMP-14 than to the two variants of MMP-9, with K_i values of 0.89 μM, 19 μM and 30 μM, respectively (Table 4) [31]. Induced fit docking of H-1 into MMP-9 and MMP-14 was performed with both the RSS and SSS enantiomer, which differ at the chiral centre closest to the phosphinyl group (Fig 1). The X-ray structure of the SSS enantiomer in complex with MMP-12 is available (PDB ID: **5CZM**) [31], showing that the large isoxazolyl side chain of H-1 (Fig 1) penetrates deeply into the S_1' subpocket of MMP-12. Docking studies revealed that this side chain similarly enters the S_1' subpocket of MMP-9 (Fig 12) and MMP-14 (Fig 13). Both the SSS and RSS enantiomer could be docked into the active site of MMP-9 and MMP-14, but the SSS enantiomer scored better in both cases, with docking scores of -13.2 kcal/mol for MMP-9 and -9.2 kcal/mol for MMP-14. In contrast, the scoring of the RSS enantiomer was-7.2 kcal/mol for MMP-9 and -6.3 kcal/mol for MMP-14. These docking scores indicated that H-1 binds stronger to MMP-9 than MMP-14, which contradicts the experimentally determined K_i values (Table 4). In the bacterial enzymes, the isoxazolyl side chain appeared too bulky to fit into the S_1' subpocket, and no clear interactions with any of the enzyme subpockets were observed. Most likely, this explains why H-1 failed to bind PLN, TLN, and ALN.

Fig. 12. Induced fit docking of H-1 with MMP-9. The docked complex of H-1 (yellow SSS enantiomer, green RSS enantiomer) with MMP-9. Colour coding of the enzyme as in Figure 7, with H-1 atom colours matching the scheme used for H-2 in the same figure.

Fig. 13. Induced fit docking of H-1 with MMP-14. The SSS enantiomer of H-1 docked into MMP-14. The enzyme is colour coded as in Fig 7, With H-1 atom colours matching those of H-2 in the same figure.

Previously, H-1 (RXP470) (Fig 1) was tested as an inhibitor of various metalloproteases (MMP-1,-2,-3,-7,-8,-9,-10,-11,-12,-13,-14), angiotensin-converting enzyme (ACE), neprilysin (NEP), and tumour necrosis factor-α-converting enzyme (ADAM17 or TACE) [41, 56]. Consistent with our results, they found that H-1 binds stronger to MMP-14 than MMP-9, with reported K_i values of 0.14 and 1.3 μM, respectively [41]. Notably, these previously obtained K_i values were lower than those obtained in the present study (Table 4). H-2 ($RXPO_3$) was also tested against various matrix metalloproteases (MMP-1,-2,-7,-8,-9,-11,-14) [34]. In agreement with our findings, H-2 bound stronger to MMP-9 than to MMP-14, with K_i values of 10 nM and 40 nM, respectively. Another study showed that that the RSS enantiomer of H-2 bound stronger to MMP-14 than the RRS enantiomer, with K_i values of 90 and 386 nM, respectively [33]. In both studies, the reported K_i values were lower than those obtained in the present study for the RSS enantiomer of H-2. Differences in the experimental setup between the current and previous studies of H-1 and H-2 [33, 34, 41] may explain these discrepancies. Factors such as buffer compositions, pH and assay reaction temperature likely influenced the results. Previous studies showed that the binding of H-1 (RXP470) to MMP-12 is pH dependent, with the K_i value increasing approximately ten times for each unit increase in buffer pH [31, 32]. Additionally, assay temperature can impact binding affinity, as seen in *Drosophila lebanonensis* alcohol dehydrogenase (ADH), where the dissociation constant ($K_{EO,I}$) of pyrazole from the enzyme-NAD^+-pyrazole complex increased with rising temperature in the pH range of 7-10 [57]. In the present study, both the buffer pH and assay temperature were higher than in previous studies, which may at least partially explain the higher K_i values observed for H-1 and H-2 with MMP-9 and MMP-14.

The phosphinic di- and tri-peptide compounds H-3 to H-5, featuring either a carboxylic acid or a carboxylic ethyl ester at the C-terminal P_1' position (Fig 1), were previously studied as potential carboxy-exopeptidase inhibitors. H-4, for instance, was among several phosphinic di- and tri-peptide compounds designed and tested against angiotensin-converting enzyme 2 (ACE2), carboxypeptidase A, and ACE [44]. Given their design and prior evaluation, it was unsurprising that H-3 to H-5 exhibited weak or no inhibition of the five proteases investigated in the present work (Fig 6).

Conclusions

The MALDI-TOF MS results showed that the substrate ES005 (Mca-Arg-Pro-Pro-Gly-Phe-Ser-Ala-Phe-Lys(Dnp)-OH) was cleaved by TLN at three distinct sites: Ala-Phe, Gly-Phe, and Ser-Ala. The fraction of cleavage products at short incubation times followed the order: Ala-Phe > Gly-Phe >> Ser-Ala. Based on these results, three ES005-TLN complexes were constructed through molecular modelling, with the cleavage site amino acids within the S_1- and S_1'-subpockets of TLN. MD simulations revealed that the structure of ES005 remained significantly more stable relative to TLN when the Ala-Phe and Gly-Phe pairs occupied the S_1- and S_1' subpockets, compared to the Ser-Ala pair. The interaction fractions of amino acids participating in non-bonded interactions with ES005 were also much higher for the Ala-Phe and Gly-Phe pairs than for Ser-Ala. These findings agree with the previously suggested substrate specificity of TLN.

Compound H-1 inhibited MMP-14 and two variants of MMP-9, with inhibition constants ranging from 0.89 to 30 μM, but showed no inhibition of the bacterial enzymes. Induced fit docking indicated that the bulky isoxazyl side chain was too large to fit the S_1' subpocket of the bacterial enzymes, and no clear interactions with the enzyme subpockets were observed, which

likely explains the lack of binding to PLN, TLN and ALN. In contrast, compound H-2 inhibited the human MMPs with inhibition constants ranging from 0.53 μM (MMP-9) to 3.0 μM (MMP-14), and the bacterial enzymes with inhibition constants ranging from 2.5 μM (TLN) to 80 μM (ALN). Induced fit docking indicated that the main difference in binding mode was that, in the bacterial MPs, the phenyl ring closest to the phosphinyl group of compound H-2 entered the S_1' subpocket, whereas in the human MMPs, the larger propylphenyl group occupied this pocket.

Supporting information

S1 Table. MALDI-TOF MS analysis. MS data observed at time zero after incubation of 50 μM Mca-Arg-Pro-Pro-Gly-Phe-Ser-Ala-Phe-Lys(Dnp)-OH (ES005) with 10 nM thermolysin (TLN).

S1 Fig. MALDI-TOF MS analysis. MS spectra at different time points after incubation of 50 μM Mca-Arg-Pro-Pro-Gly-Phe-Ser-Ala-Phe-Lys(Dnp)-OH (ES005) with 5 or 10 nM thermolysin (TLN). The m/z values marked in red, blue and green represent N-terminal cleavage products after Gly-Phe, Ser-Ala, and Ala-Phe cleavage, respectively.

S2 Fig. MALDI-TOF MS analysis. Relative intensities in percent of substrate Mca-Arg-Pro-Pro-Gly-Phe-Ser-Ala-Phe-Lys(Dnp)-OH (ES005) and cleavage products at 1, 3, 5, 10, 20 and 60 minutes of incubation of 50 μM ES005 with 5 and 10 nM thermolysin (TLN). The m/z value of 1388 represents the substrate ES005, whereas the m/z values of 947, 876 and 642 represent the N-terminal cleavage products after Ala-Phe, Ser-Ala, and Gly-Phe cleavage, respectively.

S3 Fig. MALDI-TOF MS analysis. MS spectra at different time points after incubation of 50 μM Mca-Arg-Pro-Pro-Gly-Phe-Ser-Ala-Phe-Lys(Dnp)-OH (ES005) with 100 nM thermolysin

(TLN).The m/z values marked in red, blue and green, represents N-terminal cleavage products after Gly-Phe, Ser-Ala and Ala-Phe cleavage, respectively.

S4 Fig. Root means square deviations (RMSDs) during MDs. RMSDs of enzyme and ligand (Mca-Pro-Leu-Gly-Leu(Dpa)-Ala-Arg-NH_2, ES001) from the starting enzyme-ES001 complexes during 200 ns of molecular dynamics (MD) simulations. Above: ES001 with MMP-9. Below: ES001 with MMP-14.

S5 Fig. ES001-enzyme interactions. Interaction fractions of amino acids of MMP-9 (above) and MMP-14 (below) with ES001 during 200 ns of MD simulation. Interaction fractions higher than one for the same subtype of interaction indicate that the amino acid makes multiple contacts with the substrate.

S6 Fig. Root means square deviations (RMSDs) during MDs. RMSDs of thermolysin (TLN) and ligand (Mca-Arg-Pro-Pro-Gly-Phe-Ser-Ala-Phe-Lys(Dnp)-OH, ES005) from starting TLN-ES005 complexes during 200 ns of MD simulations.

S7 Fig. ES005-TLN interactions. Interaction fraction of amino acids in TLN with the substrate Mca-Arg-Pro-Pro-Gly-Phe-Ser-Ala-Phe-Lys(Dnp)-OH (ES005) during 200 ns of MD simulations with Gly-Phe, Ala-Phe, and Ser-Ala as P_1 and P_1' residues. Interaction fractions higher than one for the same subtype of interaction indicate that the amino acid makes multiple contacts with the substrate.

S8 Fig. Amino acid sequence alignments. Amino acid sequence alignments of the catalytic domain of pseudolysin (PLN), thermolysin (TLN), and aureolysin (ALN)**.** SWISS-PROT accession codes; PLN: P14756, TLN: P00800, ALN: P81177. The alignments were generated using Clustal Omega at the online services of EMBL-EBI (http://www.ebi.ac.uk/services). The zinc binding motif and the catalytic glutamic acid are highlighted in bold.

S9 Fig. Time dependent inhibition. Time dependent inhibitory effects of 100 µM of the compounds (H1-H5) on the activity of aureolysin (ALN), pseudolysin (PLN), thermolysin (TLN), MMP-14, MMP-9 (A) and MMP-9 (MT). The experiments were performed as described in the material and methods section using a concentration of 4 µM of the substrate Mca-Pro-Leu-Gly(Dpa)-Ala-Arg-NH_2 (ES001) for MMP-9 (A), MMP-9 (T) and MMP-14, and of the substrate Mca-Arg-Pro-Pro-Gly-Phe-Ser-Ala-Phe-Lys(Dnp)-OH (ES005) for TLN, ALN and PLN. Concentrations of the enzymes were as follows: 0.05 nM MMP-9(A), 0.05 nM MMP-9(MT), 1.0 nM MMP-14, 1.4 nM ALN, 0.5 nM PLN and 0.21 nM TLN. The v_i/v_0 (mean ± s.d.) were based on 3-7 experiments.

S10 Fig. **Induced fit docking of H-2 with MMP-9.** 2D illustrations of the interactions. Amino acids within 4 Å of H-2 are included.

S11 Fig. **Induced fit docking of H-2 with MMP-14**. 2D illustrations of the interactions. Amino acids within 4 Å of H-2 are included.

S12 Fig. **Induced fit docking of H-2 with PLN**. 2D illustrations of the interactions. Amino acids within 4 Å of H-2 are included.

S13 Fig. Induced fit docking of H2 with ALN. 2D illustrations of the interactions. Amino acids within 4 Å of H-2 are included.

S14 Fig. Induced fit docking of H-2 with TLN. 2D illustrations of the interactions. Amino acids within 4 Å of H-2 are included.

S15 Fig. Induced fit docking of H-1 with MMP-9. 2D illustrations of the interactions of the SSS-enantiomer of H-1 with amino acids in MMP-9. Amino acids within 4 Å of H-1 are included.

S16 Fig. Induced fit docking of H-1 with MMP-14. 2D illustrations of the interactions of the SSS-enantiomer of H-1 with amino acids in MMP-14. Amino acids within 4 Å of H-1 are included.

Acknowledgements

The phosphinic compounds (Fig 1) were a kind gift from professor Athanasio Yiotakis, University of Athens, Greece.

Author contribution

Conceptualization: Tor Haug, Klara Stensvåg, Jan-Olof Winberg, Ingebrigt Sylte.

Data Curation: Fatema Amatur Rahman, Imin Wushur, Ida K. Ø. Hansen, Bibek Chaulagain, Tra-Mi Nguyen, Olayiwola A. Adekoya, Nabin Malla.

Formal analysis: Fatema Amatur Rahman, Imin Wushur, Ida K. Ø. Hansen, Bibek Chaulagain, Tra-Mi Nguyen, Olayiwola A. Adekoya, Nabin Malla.

Funding acquisition: Tor Haug, Klara Stensvåg, Jan-Olof Winberg, Ingebrigt Sylte.

Methodology: Fatema Amatur Rahman, Imin Wushur, Ida K. Ø. Hansen, Tor Haug, Klara Stensvåg, Jan-Olof Winberg, Ingebrigt Sylte.

Project administration: Tor Haug, Klara Stensvåg, Jan-Olof Winberg, Ingebrigt Sylte.

Software: Imin Wushur, Bibek Chaulagain.

Supervision: Tor Haug, Klara Stensvåg, Jan-Olof Winberg, Ingebrigt Sylte.

Validation: Fatema Amatur Rahman, Imin Wushur, Ida K. Ø. Hansen.

Visualization: Fatema Amatur Rahman, Imin Wushur, Ida K. Ø. Hansen, Tor Haug, Jan-Olof Winberg, Ingebrigt Sylte.

Writing – original draft: Fatema Amatur Rahman, Imin Wushur, Ida K.Ø. Hansen, Jan-Olof Winberg, Ingebrigt Sylte.

Writing – review & editing: Ida K.Ø. Hansen, Tor Haug, Klara Stensvåg, Jan-Olof Winberg, Ingebrigt Sylte.

References

1. Rawlings ND, Barrett AJ, Thomas PD, Huang X, Bateman A, Finn RD. The MEROPS database of proteolytic enzymes, their substrates and inhibitors in 2017 and a comparison with peptidases in the PANTHER database. Nucleic Acids Res. 2018;46(D1):D624-D32. Epub 2017/11/18. doi: 10.1093/nar/gkx1134. PubMed PMID: 29145643; PubMed Central PMCID: PMCPMC5753285.

2. Hadler-Olsen E, Fadnes B, Sylte I, Uhlin-Hansen L, Winberg JO. Regulation of matrix metalloproteinase activity in health and disease. FEBS J. 2011;278(1):28-45. Epub 2010/11/23. doi: 10.1111/j.1742-4658.2010.07920.x. PubMed PMID: 21087458.

3. Adekoya OA, Sylte I. The thermolysin family (M4) of enzymes: therapeutic and biotechnological potential. Chem Biol Drug Des. 2009;73(1):7-16. Epub 2009/01/21. doi: 10.1111/j.1747-0285.2008.00757.x. PubMed PMID: 19152630.

4. Ugalde AP, Ordonez GR, Quiros PM, Puente XS, Lopez-Otin C. Metalloproteases and the degradome. Methods Mol Biol. 2010;622:3-29. doi: 10.1007/978-1-60327-299-5_1. PubMed PMID: 20135273.

5. Cui N, Hu M, Khalil RA. Biochemical and biological attributes of matrix metalloproteinases. Prog Mol Biol Transl Sci. 2017;147:1-73. Epub 20170322. doi: 10.1016/bs.pmbts.2017.02.005. PubMed PMID: 28413025; PubMed Central PMCID: PMCPMC5430303.

6. Kessenbrock K, Plaks V, Werb Z. Matrix metalloproteinases: regulators of the tumor microenvironment. Cell. 2010;141(1):52-67. doi: 10.1016/j.cell.2010.03.015. PubMed PMID: 20371345; PubMed Central PMCID: PMCPMC2862057.

7. Tokito A, Jougasaki M. Matrix Metalloproteinases in non-neoplastic disorders. Int J Mol Sci. 2016;17(7). Epub 20160721. doi: 10.3390/ijms17071178. PubMed PMID: 27455234; PubMed Central PMCID: PMCPMC4964549.

8. Zhang X, Huang S, Guo J, Zhou L, You L, Zhang T, et al. Insights into the distinct roles of MMP-11 in tumor biology and future therapeutics (Review). Int J Oncol. 2016;48(5):1783-93. Epub 20160218. doi: 10.3892/ijo.2016.3400. PubMed PMID: 26892540.

9. Biasone A, Tortorella P, Campestre C, Agamennone M, Preziuso S, Chiappini M, et al. alpha-Biphenylsulfonylamino 2-methylpropyl phosphonates: enantioselective synthesis and selective inhibition of MMPs. Bioorg Med Chem. 2007;15(2):791-9. Epub 20061025. doi: 10.1016/j.bmc.2006.10.047. PubMed PMID: 17088065.

10. Campestre C, Agamennone M, Tortorella P, Preziuso S, Biasone A, Gavuzzo E, et al. N-Hydroxyurea as zinc binding group in matrix metalloproteinase inhibition: mode of binding in a complex with MMP-8. Bioorg Med Chem Lett. 2006;16(1):20-4. Epub 20051018. doi: 10.1016/j.bmcl.2005.09.057. PubMed PMID: 16242329.

11. Campestre C, Tortorella P, Agamennone M, Preziuso S, Biasone A, Nuti E, et al. Peptidyl 3-substituted 1-hydroxyureas as isosteric analogues of succinylhydroxamate MMP inhibitors. Eur J Med Chem. 2008;43(5):1008-14. Epub 20070715. doi: 10.1016/j.ejmech.2007.07.002. PubMed PMID: 17719700.

12. Cuniasse P, Devel L, Makaritis A, Beau F, Georgiadis D, Matziari M, et al. Future challenges facing the development of specific active-site-directed synthetic inhibitors of MMPs. Biochimie. 2005;87(3-4):393-402. doi: 10.1016/j.biochi.2004.09.025. PubMed PMID: 15781327.

13. Devel L, Garcia S, Czarny B, Beau F, LaJeunesse E, Vera L, et al. Insights from selective non-phosphinic inhibitors of MMP-12 tailored to fit with an S1' loop canonical conformation. J Biol Chem. 2010;285(46):35900-9. Epub 20100903. doi: 10.1074/jbc.M110.139634. PubMed PMID: 20817735; PubMed Central PMCID: PMCPMC2975213.

14. Dive V, Georgiadis D, Matziari M, Makaritis A, Beau F, Cuniasse P, et al. Phosphinic peptides as zinc metalloproteinase inhibitors. Cell Mol Life Sci. 2004;61(16):2010-9. doi: 10.1007/s00018-004-4050-y. PubMed PMID: 15316651.

15. Dive V, Lucet-Levannier K, Georgiadis D, Cotton J, Vassiliou S, Cuniasse P, et al. Phosphinic peptide inhibitors as tools in the study of the function of zinc metallopeptidases. Biochem Soc Trans. 2000;28(4):455-60. PubMed PMID: 10961939.

16. Georgiadis D, Yiotakis A. Specific targeting of metzincin family members with small-molecule inhibitors: progress toward a multifarious challenge. Bioorg Med Chem. 2008;16(19):8781-94. Epub 2008/09/16. doi: 10.1016/j.bmc.2008.08.058. PubMed PMID: 18790648.

17. Jacobsen JA, Major Jourden JL, Miller MT, Cohen SM. To bind zinc or not to bind zinc: an examination of innovative approaches to improved metalloproteinase inhibition. Biochim Biophys Acta. 2010;1803(1):72-94. Epub 20090825. doi: 10.1016/j.bbamcr.2009.08.006. PubMed PMID: 19712708.

18. Rubino MT, Agamennone M, Campestre C, Fracchiolla G, Laghezza A, Loiodice F, et al. Synthesis, SAR, and biological evaluation of alpha-sulfonylphosphonic acids as selective matrix metalloproteinase inhibitors. ChemMedChem. 2009;4(3):352-62. doi: 10.1002/cmdc.200800324. PubMed PMID: 19204958.

19. Fields GB. The rebirth of matrix metalloproteinase inhibitors: moving beyond the dogma. Cells. 2019;8(9). Epub 2019/08/30. doi: 10.3390/cells8090984. PubMed PMID: 31461880; PubMed Central PMCID: PMCPMC6769477.

20. Fields GB. Mechanisms of action of novel drugs targeting angiogenesis-promoting matrix metalloproteinases. Front Immunol. 2019;10:1278. Epub 2019/06/20. doi: 10.3389/fimmu.2019.01278. PubMed PMID: 31214203; PubMed Central PMCID: PMCPMC6558196.

21. Dickey SW, Cheung GYC, Otto M. Different drugs for bad bugs: antivirulence strategies in the age of antibiotic resistance. Nat Rev Drug Discov. 2017;16(7):457-71. Epub 2017/03/25. doi: 10.1038/nrd.2017.23. PubMed PMID: 28337021.

22. Kany AM, Sikandar A, Yahiaoui S, Haupenthal J, Walter I, Empting M, et al. Tackling *Pseudomonas aeruginosa* virulence by a hydroxamic acid-based lasB inhibitor. ACS Chem Biol. 2018;13(9):2449-55. Epub 2018/08/09. doi: 10.1021/acschembio.8b00257. PubMed PMID: 30088919.

23. Munguia J, Nizet V. Pharmacological targeting of the host-pathogen interaction: alternatives to classical antibiotics to combat drug-resistant superbugs. Trends Pharmacol Sci. 2017;38(5):473-88. Epub 2017/03/12. doi: 10.1016/j.tips.2017.02.003. PubMed PMID: 28283200; PubMed Central PMCID: PMCPMC5750058.

24. Jurado-Martin I, Sainz-Mejias M, McClean S. *Pseudomonas aeruginosa*: an audacious pathogen with an adaptable arsenal of virulence factors. Int J Mol Sci. 2021;22(6). Epub 20210318. doi: 10.3390/ijms22063128. PubMed PMID: 33803907; PubMed Central PMCID: PMCPMC8003266.

25. Gellatly SL, Hancock RE. *Pseudomonas aeruginosa*: new insights into pathogenesis and host defenses. Pathog Dis. 2013;67(3):159-73. Epub 20130315. doi: 10.1111/2049-632X.12033. PubMed PMID: 23620179.

26. Tacconelli E, Carrara E, Savoldi A, Harbarth S, Mendelson M, Monnet DL, et al. Discovery, research, and development of new antibiotics: the WHO priority list of antibiotic-resistant bacteria and tuberculosis. Lancet Infect Dis. 2018;18(3):318-27. Epub 20171221. doi: 10.1016/S1473-3099(17)30753-3. PubMed PMID: 29276051.

27. Jaradat ZW, Ababneh QO, Sha'aban ST, Alkofahi AA, Assaleh D, Al Shara A. Methicillin resistant *Staphylococcus aureus* and public fomites: a review. Pathog Glob Health. 2020;114(8):426-50. Epub 20201028. doi: 10.1080/20477724.2020.1824112. PubMed PMID: 33115375; PubMed Central PMCID: PMCPMC7759291.

28. Lowy FD. *Staphylococcus aureus* infections. N Engl J Med. 1998;339(8):520-32. doi: 10.1056/NEJM199808203390806. PubMed PMID: 9709046.

29. Roberts S, Chambers S. Diagnosis and management of *Staphylococcus aureus* infections of the skin and soft tissue. Intern Med J. 2005;35 Suppl 2:S97-105. doi: 10.1111/j.1444-0903.2005.00983.x. PubMed PMID: 16271065.

30. Konstantinovic J, Yahiaoui S, Alhayek A, Haupenthal J, Schonauer E, Andreas A, et al. *N*-Aryl-3-mercaptosuccinimides as antivirulence agents targeting *Pseudomonas aeruginosa* elastase and *Clostridium* collagenases. J Med Chem. 2020;63(15):8359-68. Epub 2020/05/30. doi: 10.1021/acs.jmedchem.0c00584. PubMed PMID: 32470298; PubMed Central PMCID: PMCPMC7429951.

31. Rouanet-Mehouas C, Czarny B, Beau F, Cassar-Lajeunesse E, Stura EA, Dive V, et al. Zinc-metalloproteinase inhibitors: evaluation of the complex role played by the zinc-binding group on potency and selectivity. J Med Chem. 2017;60(1):403-14. Epub 2016/12/21. doi: 10.1021/acs.jmedchem.6b01420. PubMed PMID: 27996256.

32. Czarny B, Stura EA, Devel L, Vera L, Cassar-Lajeunesse E, Beau F, et al. Molecular determinants of a selective matrix metalloprotease-12 inhibitor: insights from crystallography and thermodynamic studies. J Med Chem. 2013;56(3):1149-59. Epub 20130205. doi: 10.1021/jm301574d. PubMed PMID: 23343195.

33. Makaritis A, Georgiadis D, Dive V, Yiotakis A. Diastereoselective solution and multipin-based combinatorial array synthesis of a novel class of potent phosphinic metalloprotease inhibitors. Chemistry. 2003;9(9):2079-94. doi: 10.1002/chem.200204456. PubMed PMID: 12740857.

34. Vassiliou S, Mucha A, Cuniasse P, Georgiadis D, Lucet-Levannier K, Beau F, et al. Phosphinic pseudo-tripeptides as potent inhibitors of matrix metalloproteinases: a structure-activity study. J Med Chem. 1999;42(14):2610-20. doi: 10.1021/jm9900164. PubMed PMID: 10411481.

35. Rahman F, Nguyen TM, Adekoya OA, Campestre C, Tortorella P, Sylte I, et al. Inhibition of bacterial and human zinc-metalloproteases by bisphosphonate- and catechol-containing compounds. J Enzyme Inhib Med Chem. 2021;36(1):819-30. Epub 2021/03/25. doi: 10.1080/14756366.2021.1901088. PubMed PMID: 33757387; PubMed Central PMCID: PMCPMC7993378.

36. Sjoli S, Nuti E, Camodeca C, Bilto I, Rossello A, Winberg JO, et al. Synthesis, experimental evaluation and molecular modelling of hydroxamate derivatives as zinc metalloproteinase inhibitors. Eur J Med Chem. 2016;108:141-53. Epub 2015/12/08. doi: 10.1016/j.ejmech.2015.11.019. PubMed PMID: 26638045.

37. Sjoli S, Solli AI, Akselsen O, Jiang Y, Berg E, Hansen TV, et al. PAC-1 and isatin derivatives are weak matrix metalloproteinase inhibitors. Biochim Biophys Acta. 2014;1840(10):3162-9. Epub 2014/07/22. doi: 10.1016/j.bbagen.2014.07.011. PubMed PMID: 25046380.

38. Sylte I, Dawadi R, Malla N, von Hofsten S, Nguyen TM, Solli AI, et al. The selectivity of galardin and an azasugar-based hydroxamate compound for human matrix metalloproteases and bacterial metalloproteases. PLoS One. 2018;13(8):e0200237. Epub 2018/08/04. doi: 10.1371/journal.pone.0200237. PubMed PMID: 30075004; PubMed Central PMCID: PMCPMC6075749.

39. Adekoya OA, Sjoli S, Wuxiuer Y, Bilto I, Marques SM, Santos MA, et al. Inhibition of pseudolysin and thermolysin by hydroxamate-based MMP inhibitors. Eur J Med Chem. 2015;89:340-8. Epub 2014/12/03. doi: 10.1016/j.ejmech.2014.10.009. PubMed PMID: 25462250.

40. Rahman F, Wushur I, Malla N, Astrand OAH, Rongved P, Winberg JO, et al. Zinc-chelating compounds as inhibitors of human and bacterial zinc metalloproteases. Molecules. 2021;27(1). Epub 20211222. doi: 10.3390/molecules27010056. PubMed PMID: 35011288; PubMed Central PMCID: PMCPMC8746695.

41. Devel L, Rogakos V, David A, Makaritis A, Beau F, Cuniasse P, et al. Development of selective inhibitors and substrate of matrix metalloproteinase-12. J Biol Chem. 2006;281(16):11152-60. Epub 20060215. doi: 10.1074/jbc.M600222200. PubMed PMID: 16481329.

42. Gall AL, Ruff M, Kannan R, Cuniasse P, Yiotakis A, Dive V, et al. Crystal structure of the stromelysin-3 (MMP-11) catalytic domain complexed with a phosphinic inhibitor mimicking the transition-state. J Mol Biol. 2001;307(2):577-86. doi: 10.1006/jmbi.2001.4493. PubMed PMID: 11254383.

43. Matziari M, Dellis D, Dive V, Yiotakis A, Samios J. Conformational and solvation studies via computer simulation of the novel large scale diastereoselectively synthesized phosphinic MMP inhibitor RXP03 diluted in selected solvents. J Phys Chem B. 2010;114(1):421-8. doi: 10.1021/jp903830v. PubMed PMID: 20014753.

44. Mores A, Matziari M, Beau F, Cuniasse P, Yiotakis A, Dive V. Development of potent and selective phosphinic peptide inhibitors of angiotensin-converting enzyme 2. J Med Chem. 2008;51(7):2216-26. Epub 20080307. doi: 10.1021/jm701275z. PubMed PMID: 18324760.

45. Dawadi R, Malla N, Hegge B, Wushur I, Berg E, Svineng G, et al. Molecular interactions stabilizing the promatrix metalloprotease-9.serglycin heteromer. Int J Mol Sci. 2020;21(12). Epub 20200612. doi: 10.3390/ijms21124205. PubMed PMID: 32545641; PubMed Central PMCID: PMCPMC7352350.

46. Murphy G, Crabbe T. Gelatinases A and B. Methods Enzymol. 1995;248:470-84. doi: 10.1016/0076-6879(95)48030-7. PubMed PMID: 7674939.

47. K. J. Bowers EC, H. Xu, R. O. Dror, M. P. Eastwood, B. A. Gregersen, J. L. Klepeis, I. Kolossvary, M. A. Moraes, F. D. Sacerdoti, J. K. Salmon, Y. Shan, and D. E. Shaw, editor Scalable algorithms for molecular dynamics simulations on commodity clusters. SC '06: International conference for high performance computing, networking, storage and analysis; 2006; Tampa, Florida, USA.

48. Lu C, Wu C, Ghoreishi D, Chen W, Wang L, Damm W, et al. OPLS4: Improving force field accuracy on challenging regimes of chemical space. J Chem Theory Comput. 2021;17(7):4291-300. Epub 20210607. doi: 10.1021/acs.jctc.1c00302. PubMed PMID: 34096718.

49. Woessner J, Nagase, H. Matrix metalloproteases and TIMPs. Sherterline P, editor. Oxford: Oxford University Press; 2000.

50. Tranchant I, Vera L, Czarny B, Amoura M, Cassar E, Beau F, et al. Halogen bonding controls selectivity of FRET substrate probes for MMP-9. Chem Biol. 2014;21(3):408-13. Epub 20140227. doi: 10.1016/j.chembiol.2014.01.008. PubMed PMID: 24583051.

51. Piana S, Lindorff-Larsen K, Dirks RM, Salmon JK, Dror RO, Shaw DE. Evaluating the effects of cutoffs and treatment of long-range electrostatics in protein folding simulations. PLoS One. 2012;7(6):e39918. Epub 20120629. doi: 10.1371/journal.pone.0039918. PubMed PMID: 22768169; PubMed Central PMCID: PMCPMC3386949.

52. Martyna GK, ML. Tuckerman,. Nosé–Hoover chains: The canonical ensemble via continuous dynamics. J Chem Phys. 1992;97:2635-43. doi: https://aip.scitation.org/doi/10.1063/1.463940.

53. Hangauer DG, Monzingo AF, Matthews BW. An interactive computer graphics study of thermolysin-catalyzed peptide cleavage and inhibition by N-carboxymethyl dipeptides. Biochemistry-Us. 1984;23(24):5730-41. doi: 10.1021/bi00319a011. PubMed PMID: 6525336.

54. Holden HM, Matthews BW. The binding of L-valyl-L-tryptophan to crystalline thermolysin illustrates the mode of interaction of a product of peptide hydrolysis. J Biol Chem. 1988;263(7):3256-60. doi: 10.2210/pdb3tmn/pdb. PubMed PMID: 3343246.

55. Ligne T, Pauthe E, Monti JP, Gacel G, Larreta-Garde V. Additional data about thermolysin specificity in buffer- and glycerol-containing media. Biochim Biophys Acta. 1997;1337(1):143-8. doi: 10.1016/s0167-4838(96)00142-2. PubMed PMID: 9003446.

56. Devel L, Beau F, Amoura M, Vera L, Cassar-Lajeunesse E, Garcia S, et al. Simple pseudo-dipeptides with a P2' glutamate: a novel inhibitor family of matrix metalloproteases and

other metzincins. J Biol Chem. 2012;287(32):26647-56. Epub 20120611. doi: 10.1074/jbc.M112.380782. PubMed PMID: 22689580; PubMed Central PMCID: PMCPMC3411004.

57. Winberg JO, Brendskag MK, Sylte I, Lindstad RI, McKinley-McKee JS. The catalytic triad in *Drosophila* alcohol dehydrogenase: pH, temperature and molecular modelling studies. J Mol Biol. 1999;294(2):601-16. doi: 10.1006/jmbi.1999.3235. PubMed PMID: 10610783.

Figure 1

Click here to access/download;Figure;Fig.1.eps

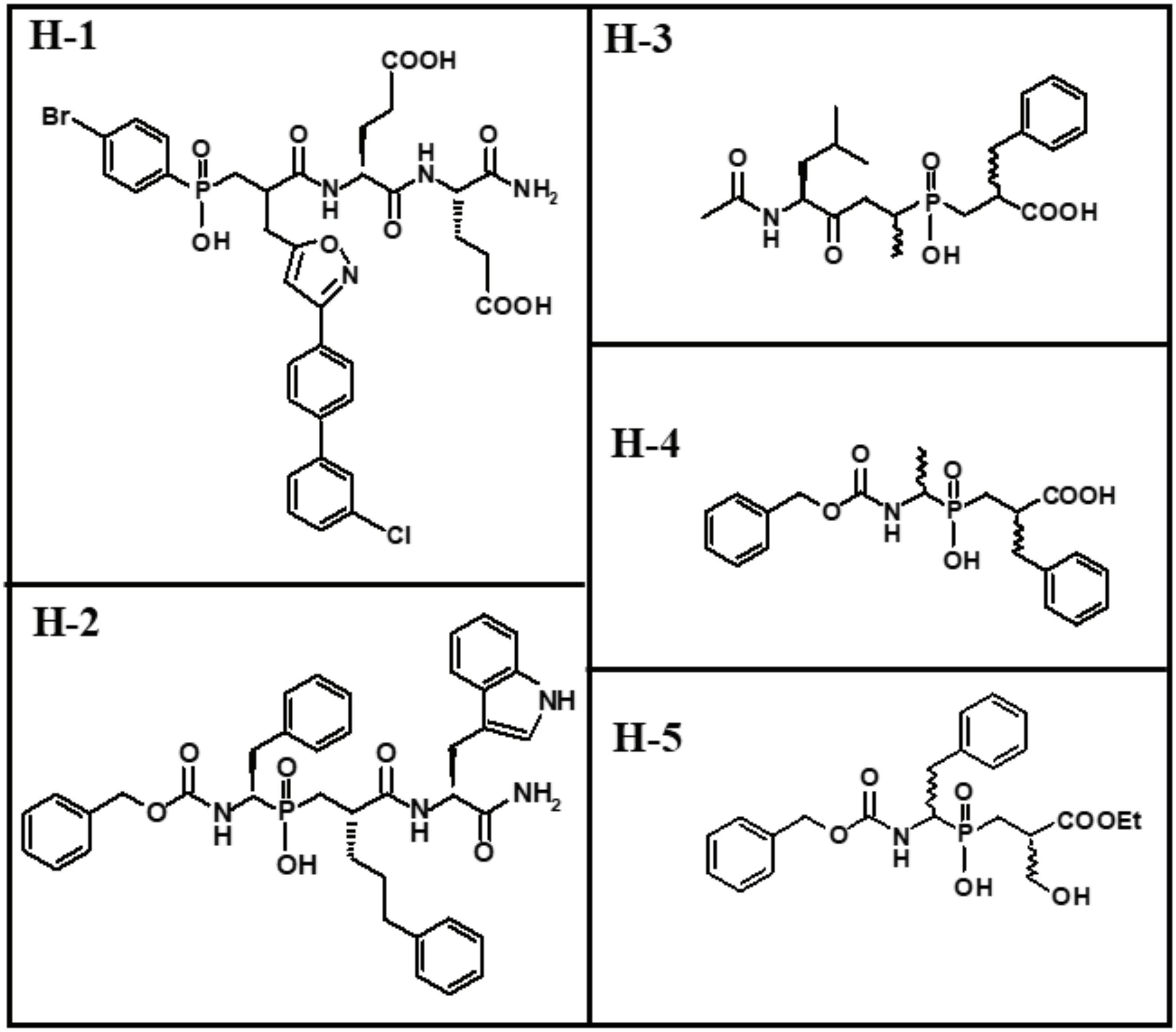

Figure 2 MMP-9 substrate probe

Click here to access/download;Figure;Fig.2.eps

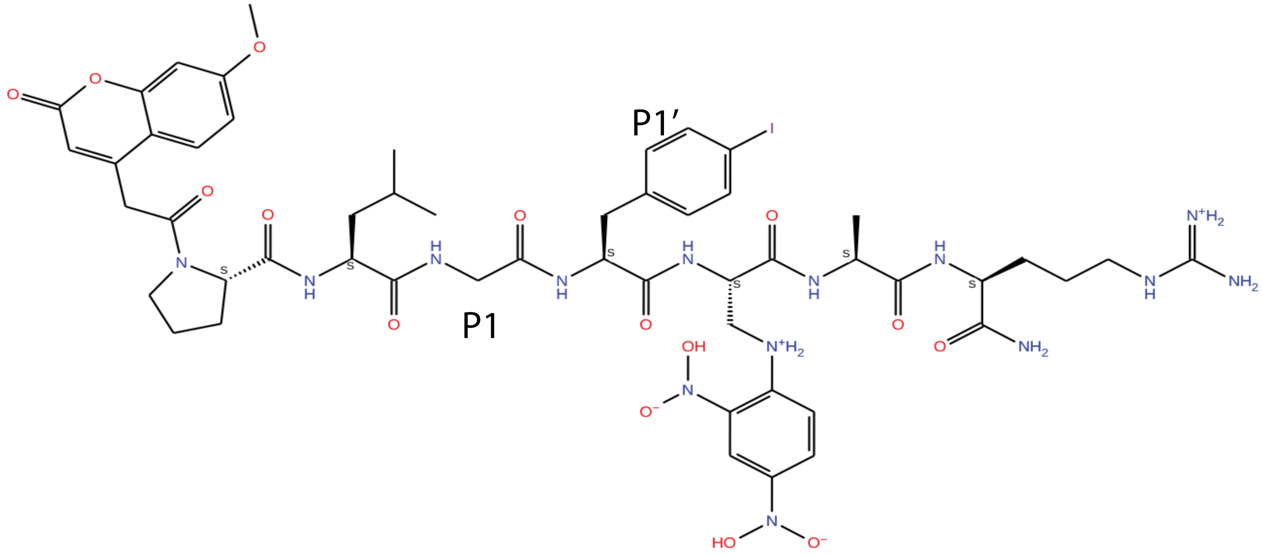

ES001

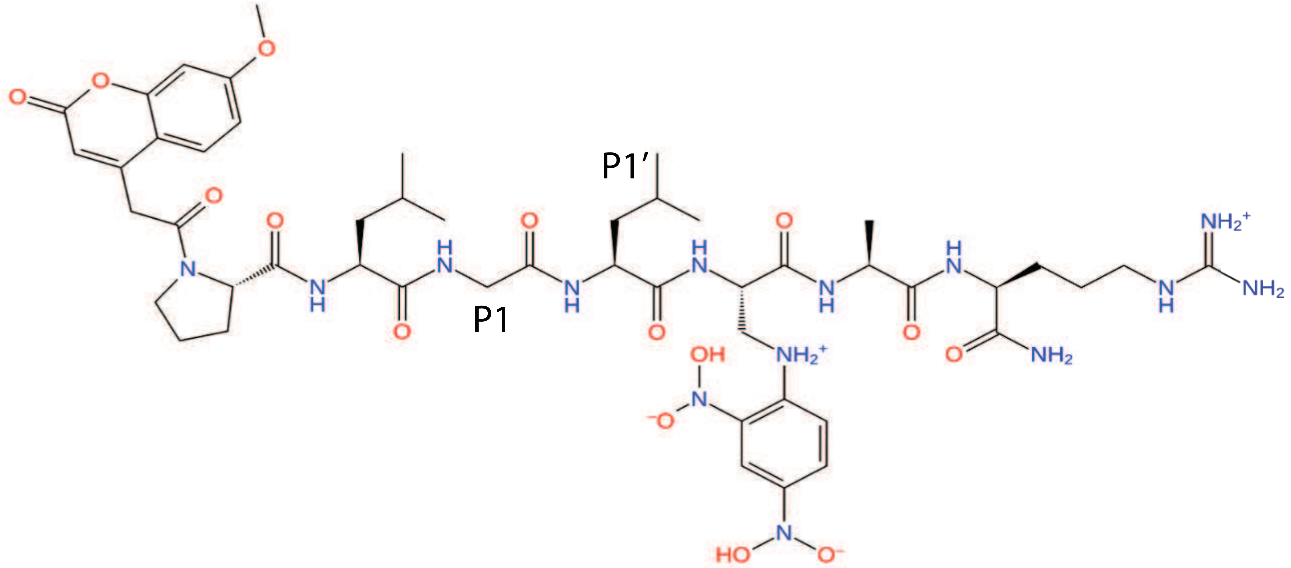

ES005

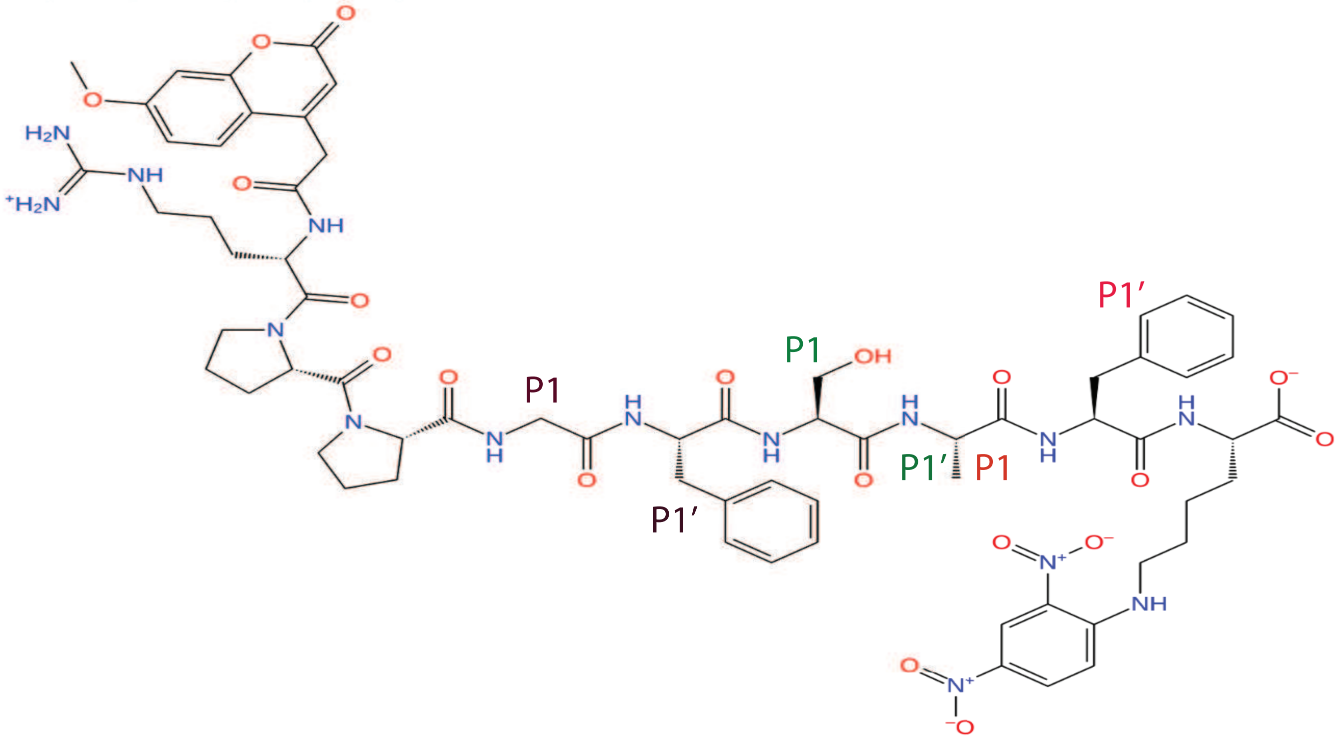

Figure 3

Click here to access/download;Figure;Fig.3.eps

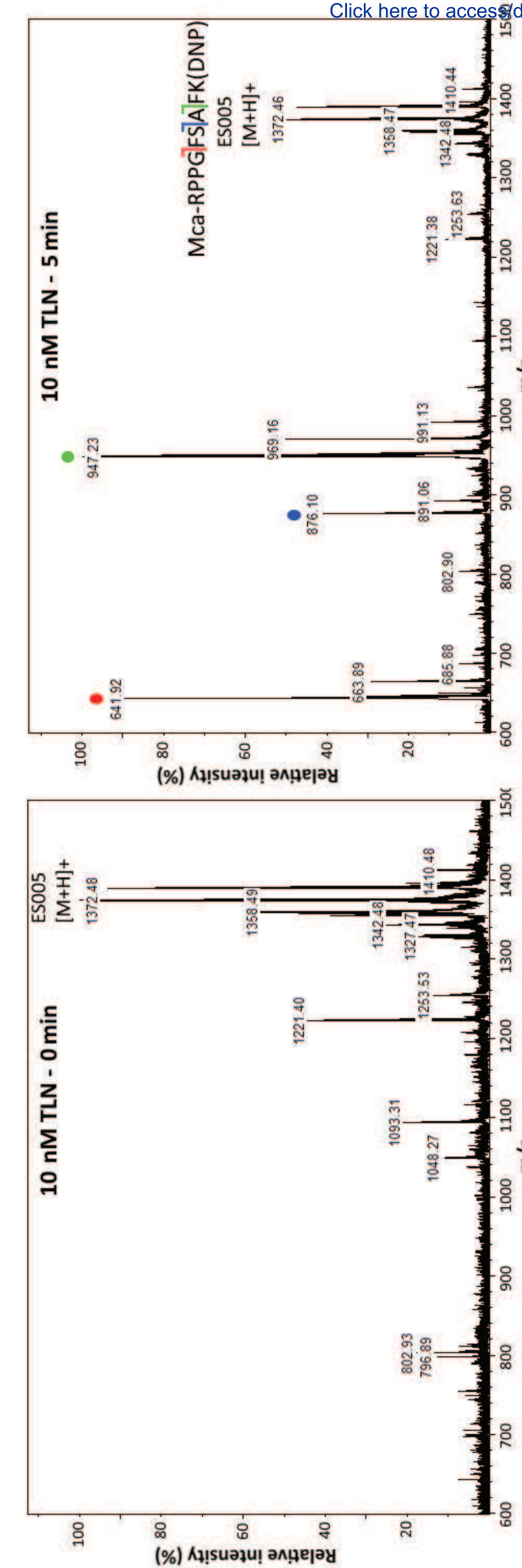

Click here to access/download;Figure;Fig.4.eps

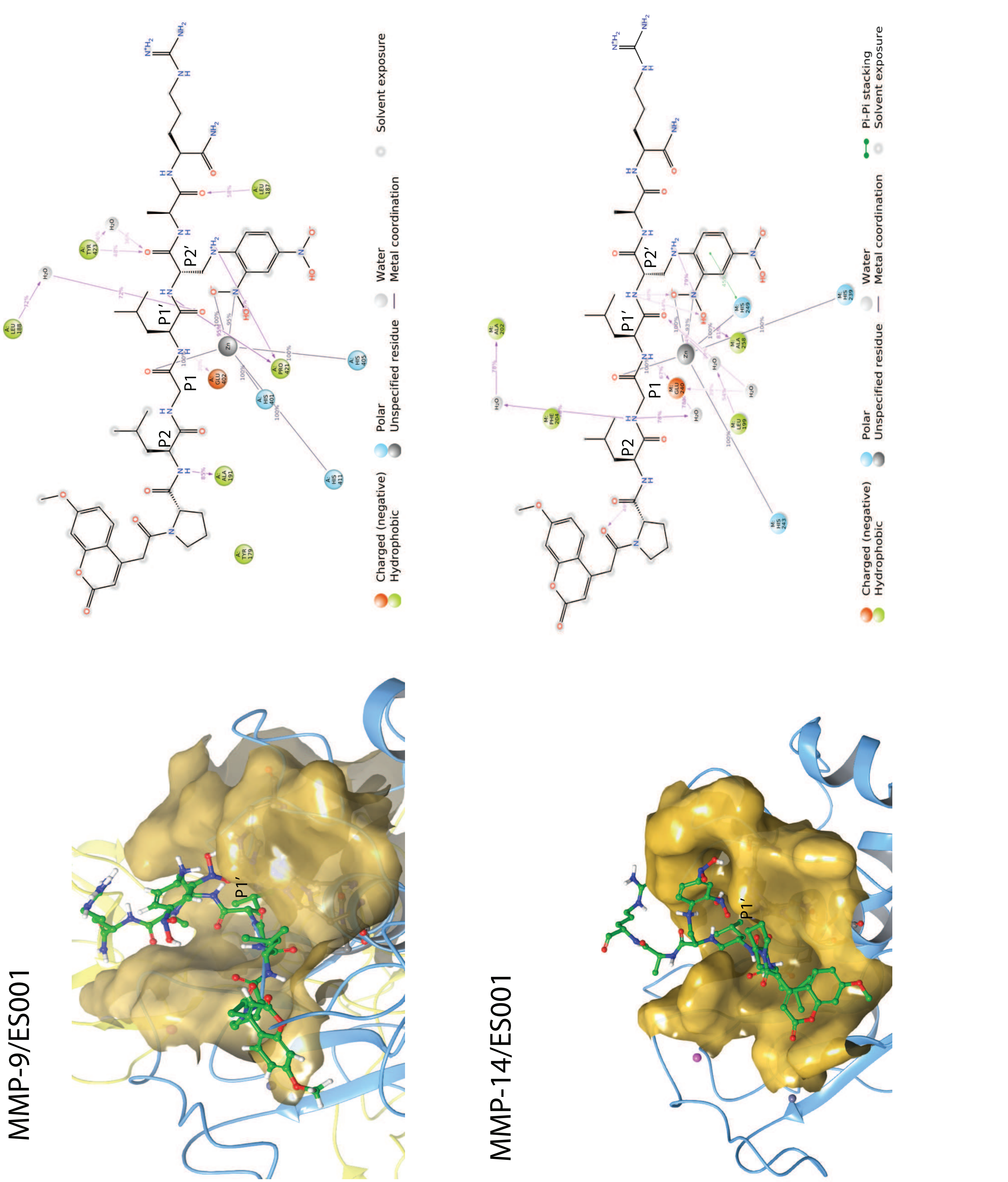

Figure 5

Click here to access/download;Figure;Fig.5.eps

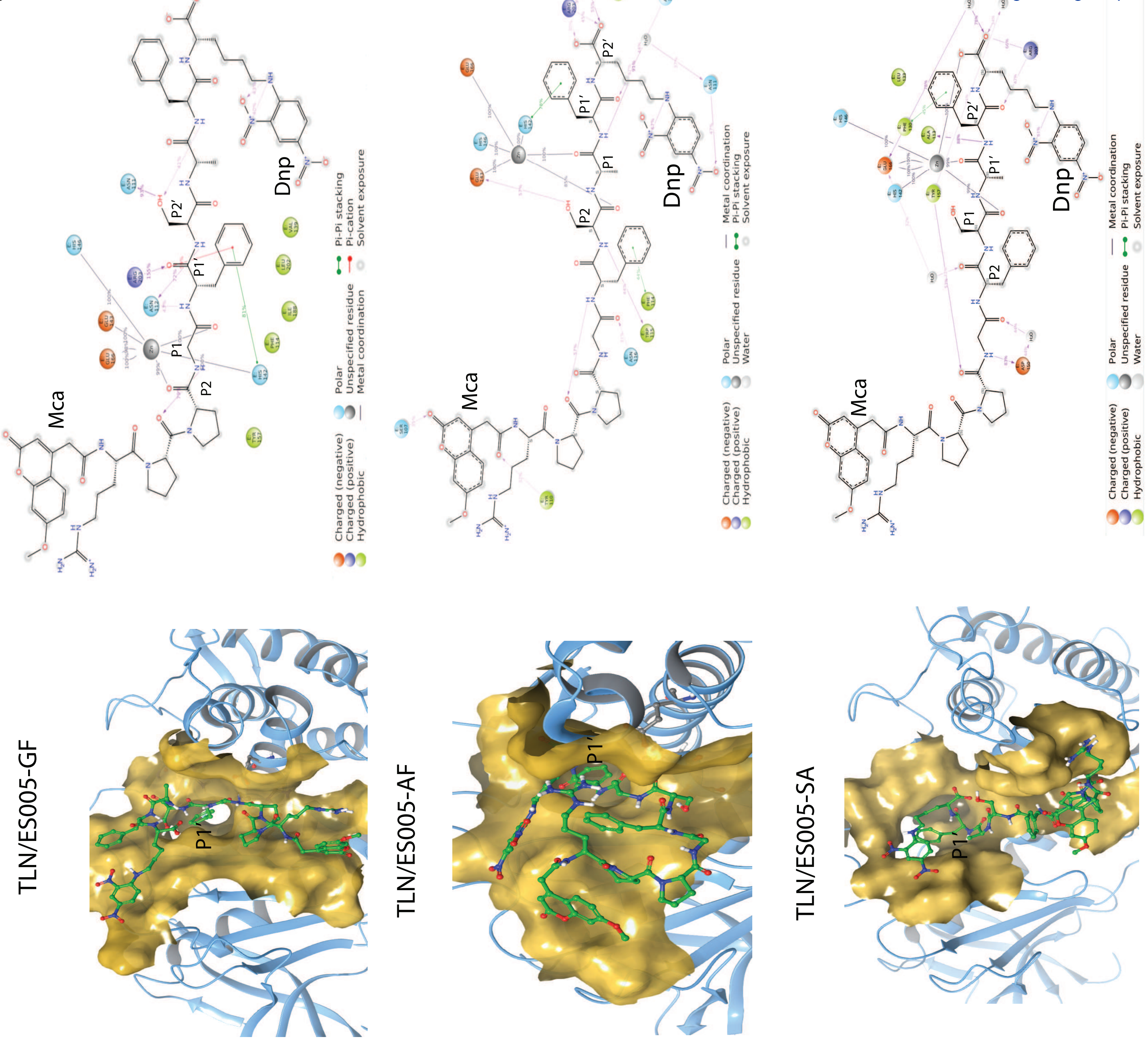

Figure 6 Click here to access/download;Figure;Fig.6.tif

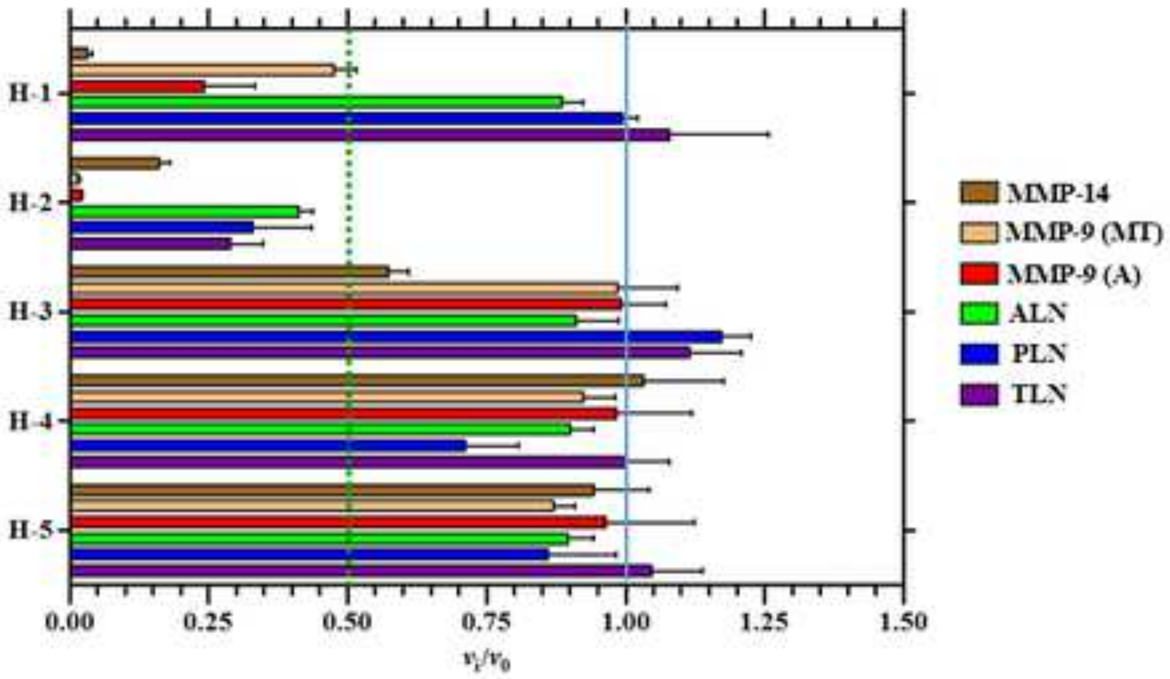

Figure 7

Click here to access/download;Figure;Fig.7.eps

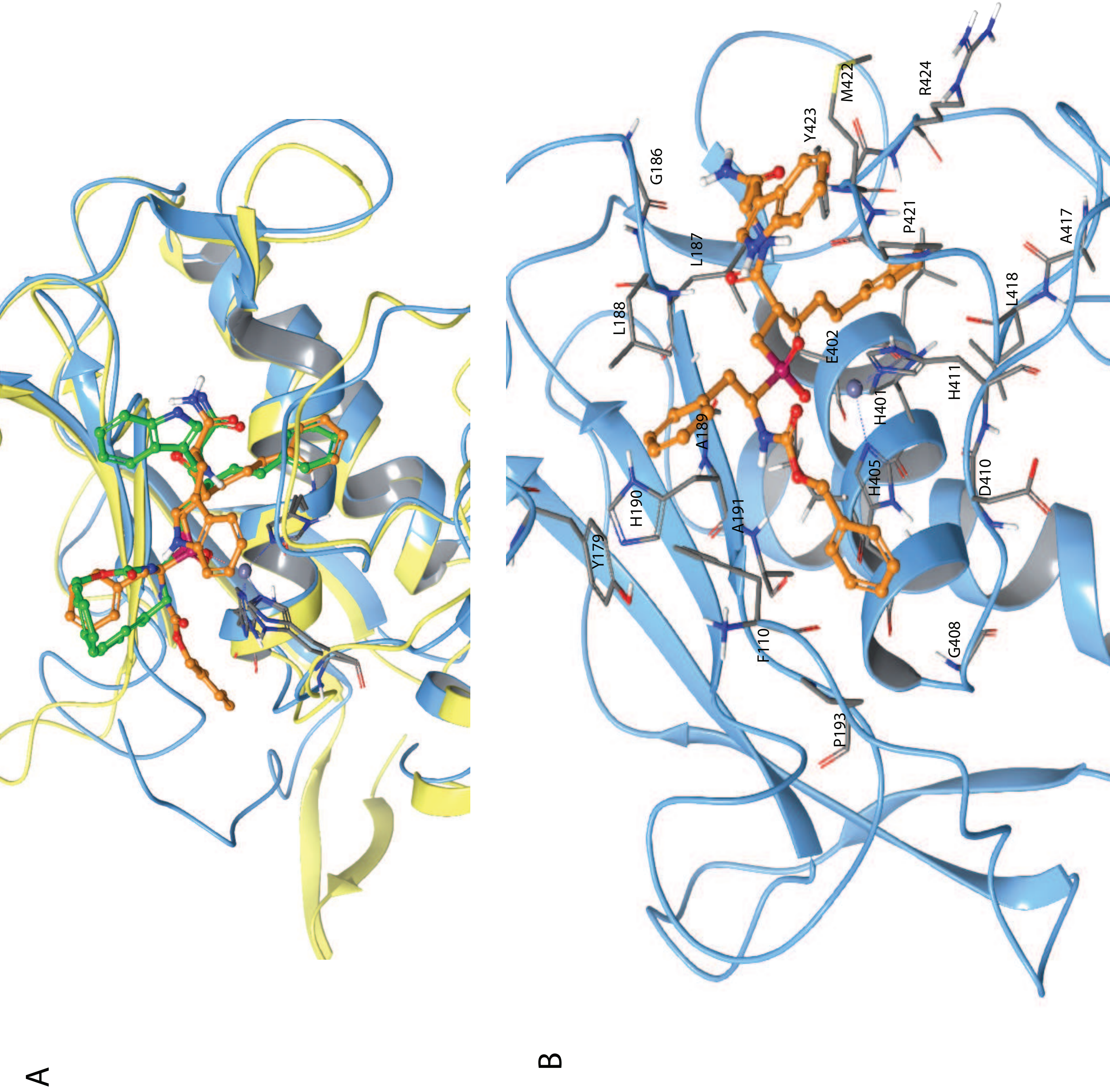

Figure 8

Click here to access/download;Figure;Fig.8.eps

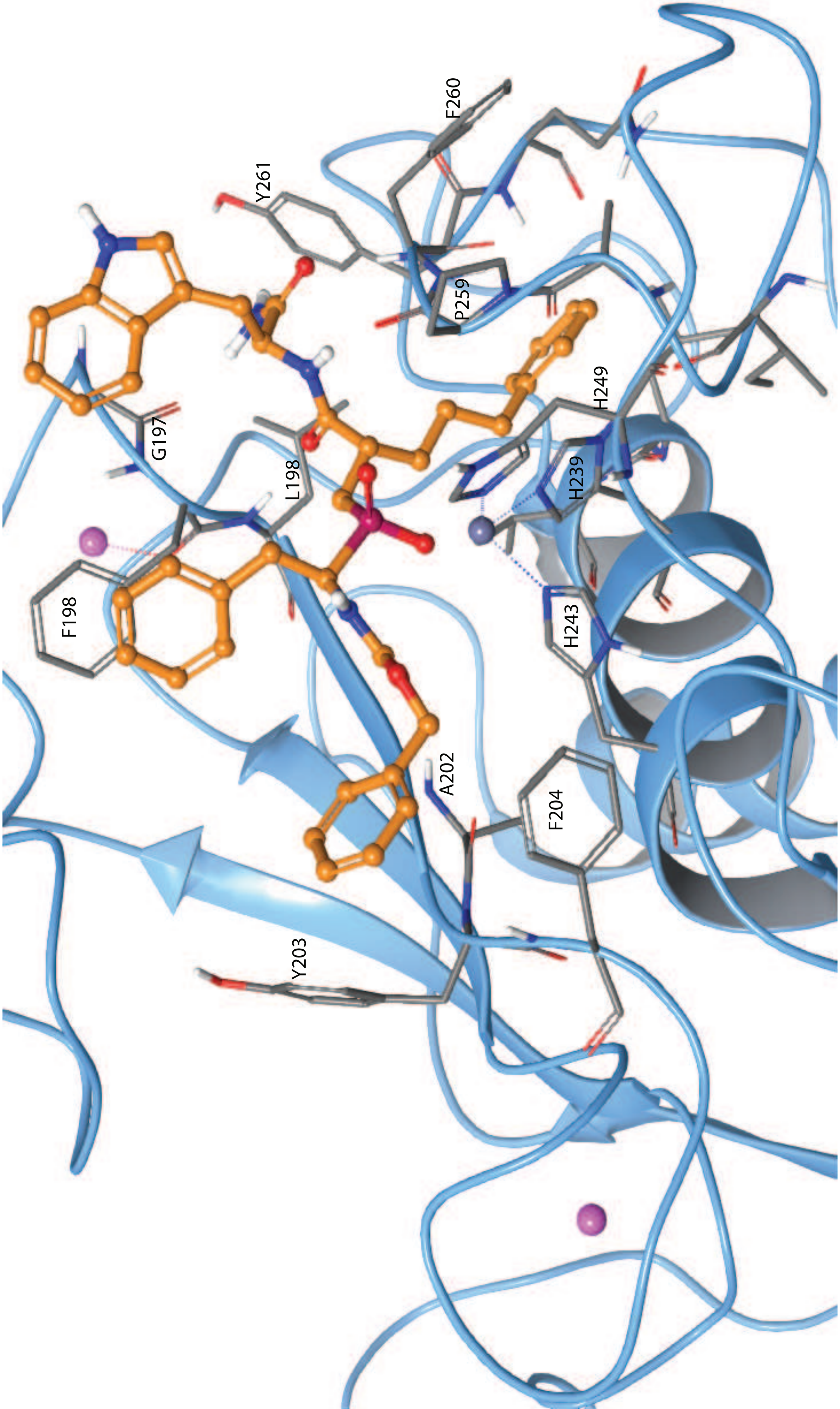

Figure 9

Click here to access/download;Figure;Fig.9.eps

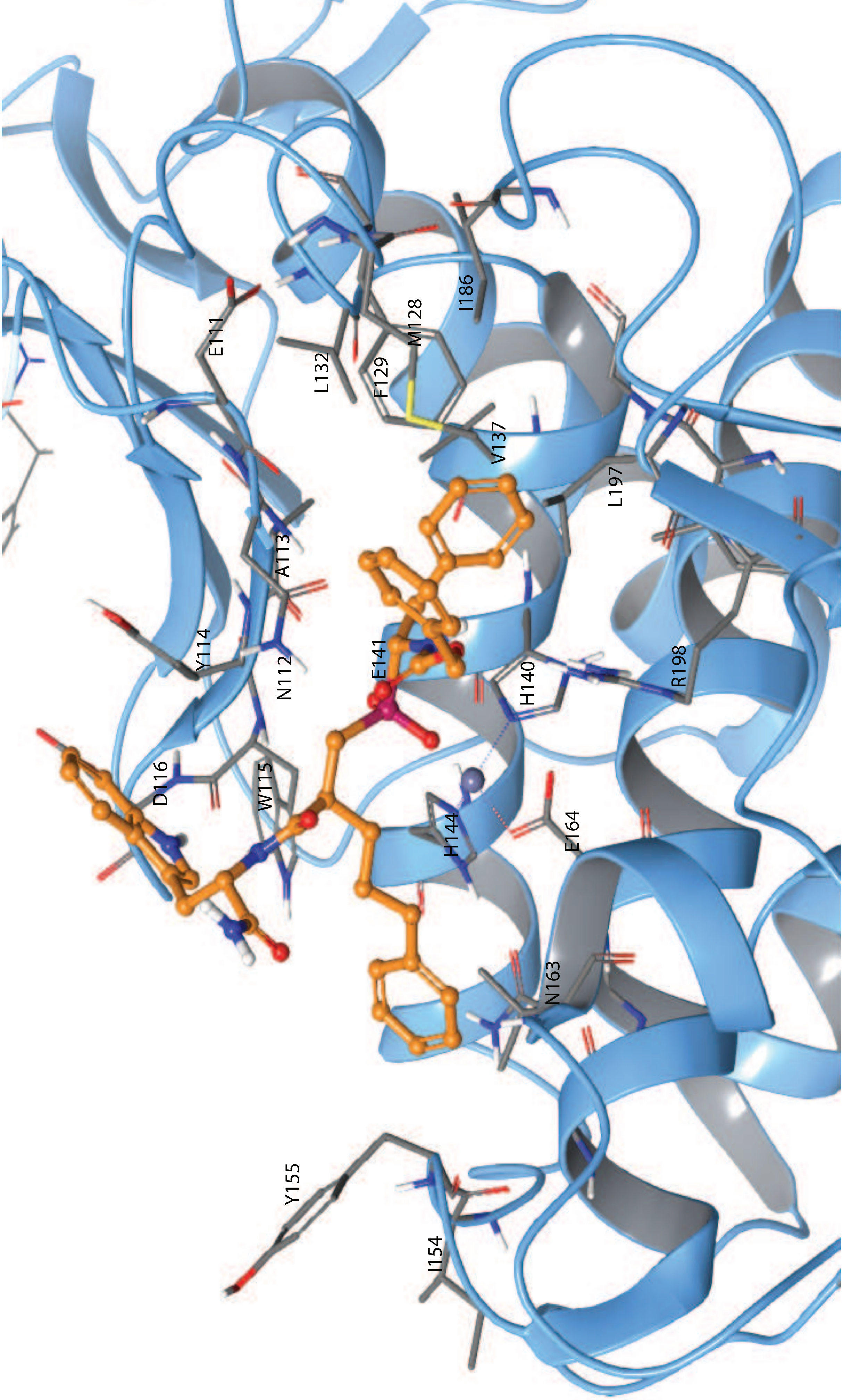

Figure 10

Click here to access/download;Figure;Fig.10.eps

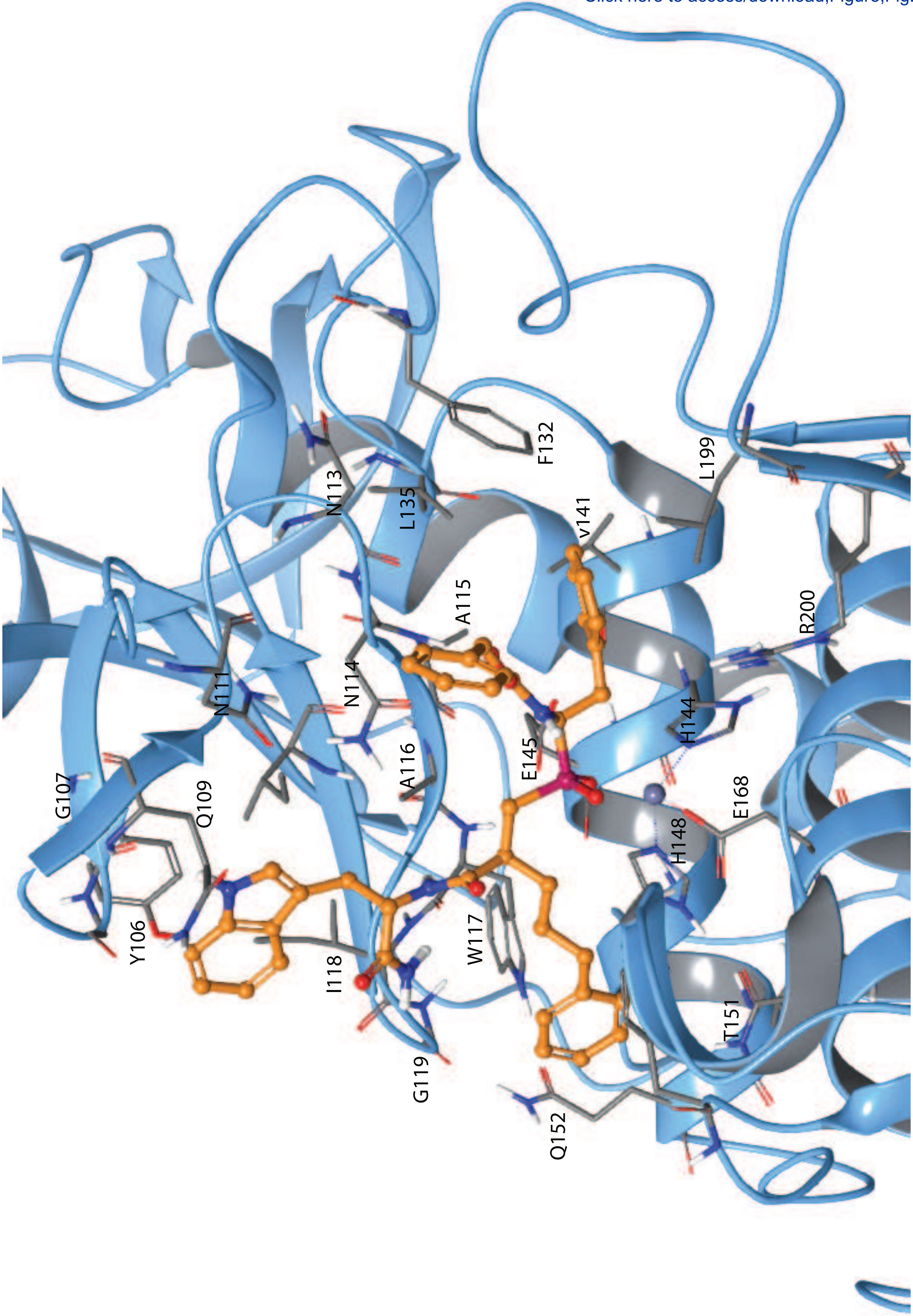

Figure 11

Click here to access/download;Figure;Fig.11.eps

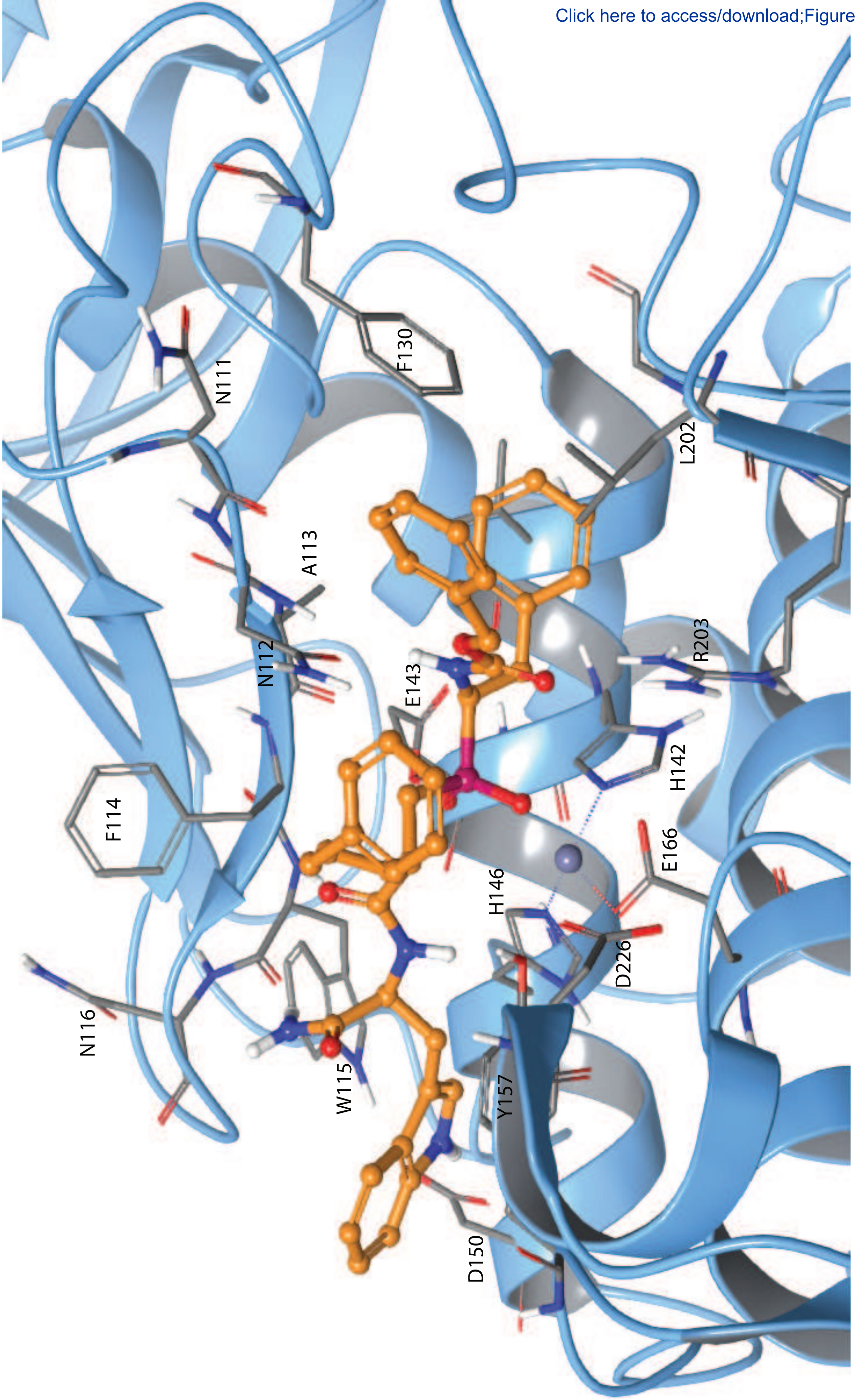

Click here to access/download;Figure;Fig.12.eps

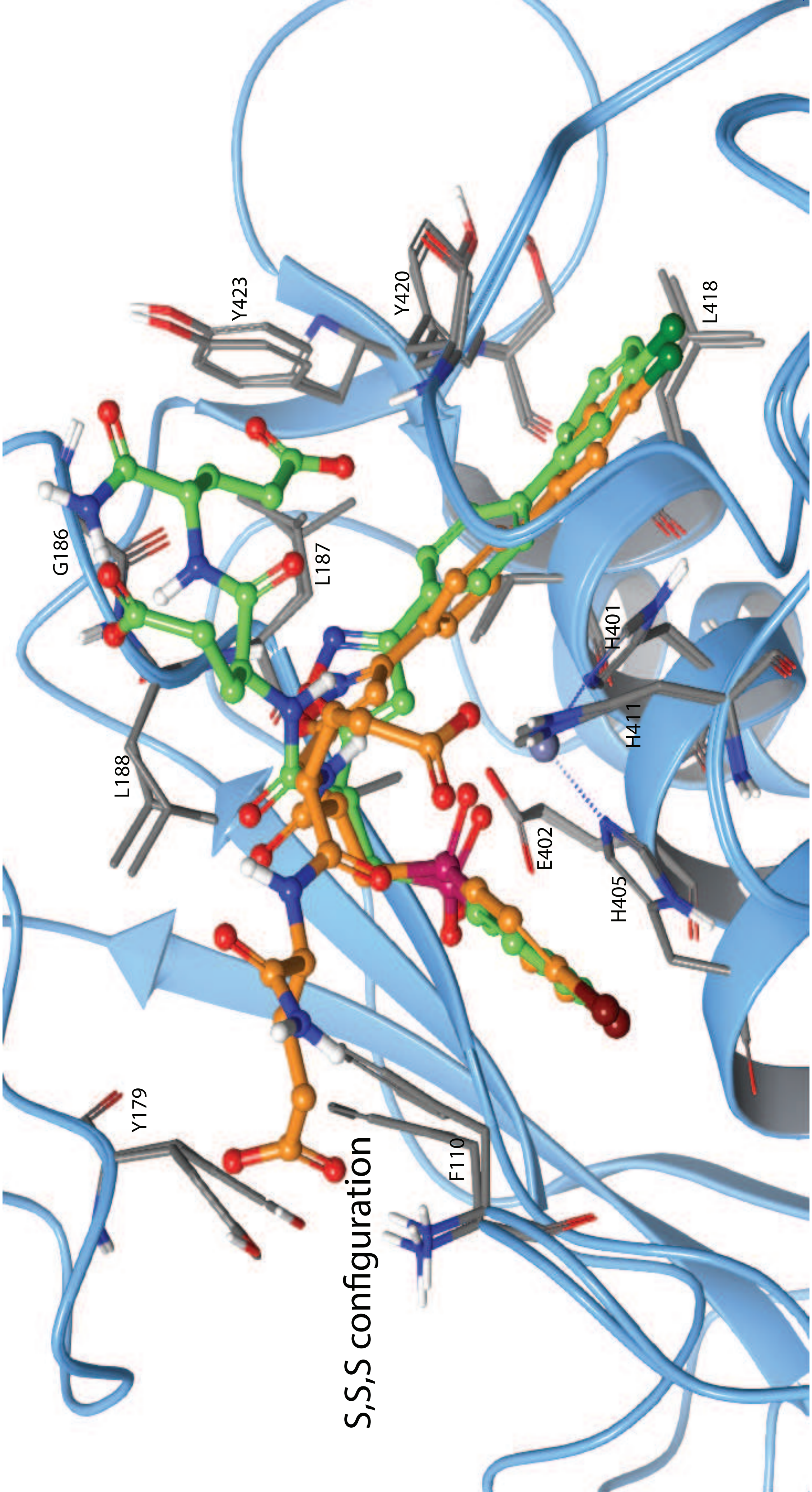

Figure 13

Click here to access/download;Figure;Fig.13.eps

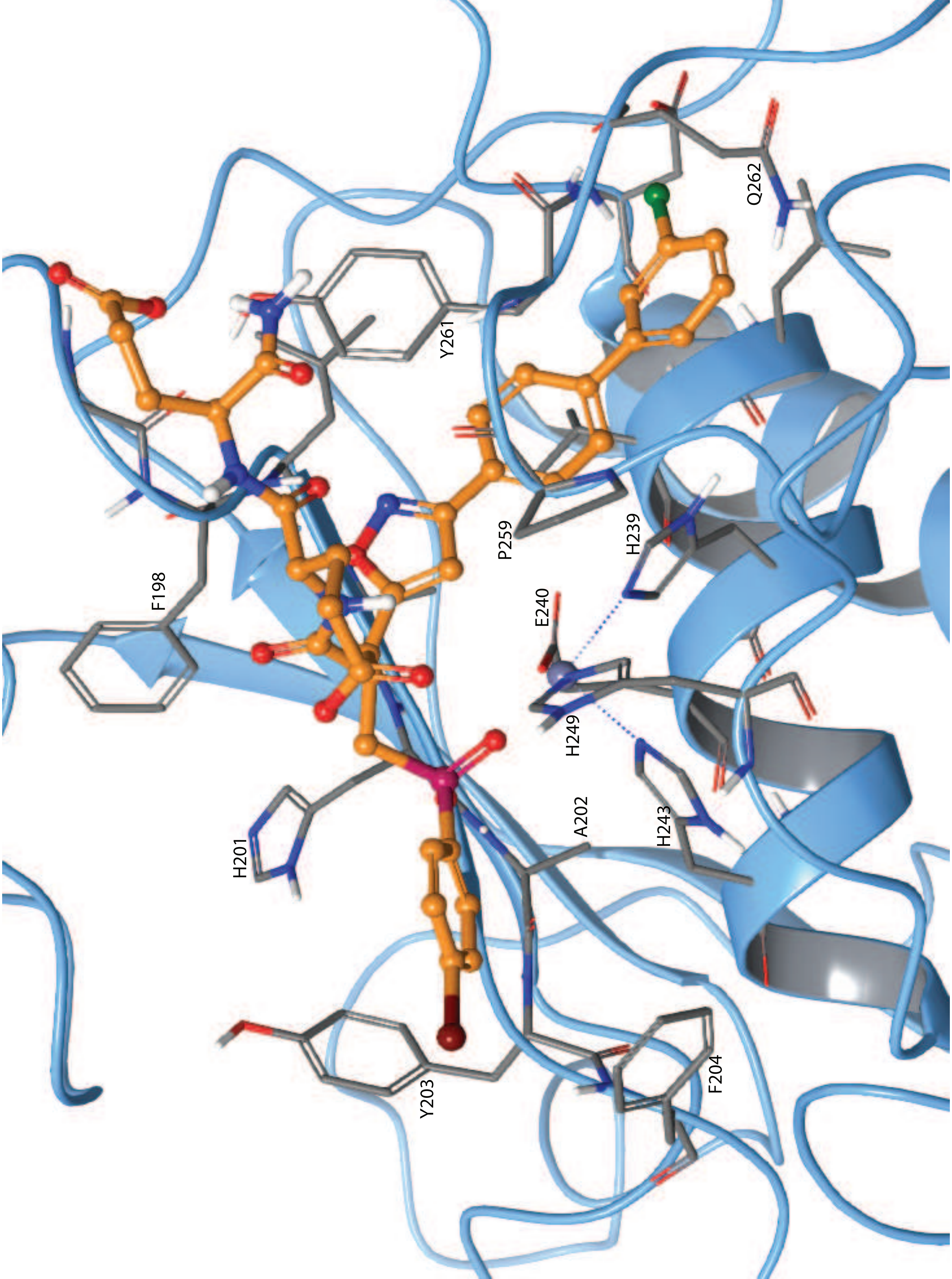

Click here to access/download
Supporting Information
Supporting Information.pdf
